# New Lead Discovery of Herbicide Safener for Metolachlor Based on a Scaffold-Hopping Strategy

**DOI:** 10.3390/molecules25214986

**Published:** 2020-10-28

**Authors:** Xile Deng, Wenna Zheng, Qingcai Zhan, Yanan Deng, Yong Zhou, Lianyang Bai

**Affiliations:** Hunan Provincial Key Laboratory for Biology and Control of Weeds, Biotechnology Research Institute, Hunan Academy of Agricultural Sciences, Changsha 410125, China; dengxile@hunaas.cn (X.D.); zhengwenna1994@163.com (W.Z.); zhanqc@hotmail.com (Q.Z.); dengyanan007@hunaas.cn (Y.D.); qimiaobuchugan@126.com (Y.Z.)

**Keywords:** sanshools, synthesis, *N*-alkylamides, natural products, herbicide safener

## Abstract

The use of herbicide safeners can significantly alleviate herbicide injury to protect crop plants and expand the application scope of the existing herbicides in the field. Sanshools, which are well known as spices, are *N*-alkyl substituted compounds extracted from the *Zanthoxylum* species and have several essential physiological and pharmacological functions. Sanshools display excellent safener activity for the herbicide metolachlor in rice seedlings. However, the high cost of sanshools extraction and difficulties in the synthesis of their complicated chemical structures limit their utilization in agricultural fields. Thus, the present study designed and synthesized various *N*-alkyl amide derivatives via the scaffold-hopping strategy to solve the challenge of complicated structures and find novel potential safeners for the herbicide metolachlor. In total, 33 *N*-alkyl amide derivatives (**2a**–**k**, **3a**–**k**, and **4a**–**k**) were synthesized using amines and saturated and unsaturated fatty acids as starting materials through acylation and condensation. The identity of all the target compounds was well confirmed by ^1^H-NMR, ^13^C-NMR, and high-resolution mass spectrometry (HRMS). The primary evaluation of safener activities for the compounds by the agar method indicated that most of the target compounds could protect rice seedlings from injury caused by metolachlor. Notably, compounds **2k** and **4k** displayed excellent herbicide safener activities on plant height and demonstrated relatively similar activities to the commercialized compound dichlormid. Moreover, we showed that compounds **2k** and **4k** had higher glutathione *S*-transferase (GST), superoxide dismutase (SOD), catalase (CAT), peroxidase (POD), and polyphenol oxidase (PPO) activities in rice seedlings, compared to the metolachlor treatment. In particular, **2k** and **4k** are safer for aquatic organisms than dichlormid. Results from the current work exhibit that compounds **2k** and **4k** have excellent crop safener activities toward rice and can, thus, be promising candidates for further structural optimization in rice protection.

## 1. Introduction

Weeds compete with crop plants for various resources, such as nutrients, water, and sunlight, which significantly affect crop productivity. Besides, weeds also increase crop protection costs because they harbor pests and bacterial diseases [1,2,3]. Herbicide application has been a prominent weed-control measure in recent decades under field conditions [4,5]. For example, metolachlor, one of the most widely used chloroacetanilide herbicides, is applied to crops (e.g., rice, corn, and soybean) to control pre-emergent and early post-emergent broadleaf and grass weeds [6,7,8,9,10,11]. However, the use of herbicides as metolachlor for weed controls is phytotoxic to crop plants [12,13,14]. 

Herbicide phytotoxicity is mainly solved through several means: (i) developing selective herbicides that are relatively safe to the crops; (ii) cultivating herbicide-resistance crop plants; (iii) using herbicide safeners [15,16,17]. Among all these methods, the application of herbicide safeners is considered the most cost-effective and widely used method [18,19,20,21]. Herbicide safeners are chemicals that reduce the phytotoxicity of herbicides to crop plants without affecting the weed-control efficacy [21]. The first commercial herbicide safener, 1,8-naphthalic anhydride (NA), was discovered by Gulf Oil Company in 1970 and can efficiently reduce the injury caused by thiocarbamate herbicides on corn [22]. Since then, nearly twenty commercial safeners have been launched on the market, such as dichlormid, benoxacor, fenclorim, cloquintocet-mexyl, and flurozle. These safeners are used to protect major cash crops against pre-plant-incorporated or pre-emergence-applied herbicides, such as chloroacetanilides and thiocarbamates [23,24]. 

The primary mechanism for safener action is the enhancement of herbicide detoxification in safened plants [21]. The ability of safeners to enhance glutathione *S*-transferase (GST) activity has been particularly well described in rice, maize, and wheat, among other crops [25,26,27]. In these cases, safener treatments may induce transcriptional activation of specific GST genes and enhance the glutathione *S*-transferase (GST) expression of the respective enzymes that catalyze the conjugation of herbicides with glutathione (GSH) in crop plants to detoxify them [28]. Besides, detoxifying enzymes, including superoxide dismutase (SOD) and peroxidase (POD), are also involved in the detoxification of some herbicides [18,29]. 

In the application of herbicide safeners in nearly 40 years, it is noted that herbicide has shown its biological activity to protect crops plants as “inert ingredients”, suggesting that they do not act on the weeds directly, and their environmental toxicity environment is not confirmed [30]. However, commercial safeners inevitably pose potential environmental pollution risks following widespread and long-term use. For instance, it has been shown that some commercial herbicide safeners are toxic to aquatic organisms, such as fish [31]. Meanwhile, the extensive application of herbicides alongside herbicides has caused the emergence of weed resistance, which could increase phytotoxicity to crop plants [9]. This fact suggests that the practice can lead to major practical problems in agricultural production, such as dramatic crop productivity losses. Therefore, the identification and development of novel eco-friendly safeners with high-efficacy is of great significance and urgency.

**Figure 1:**
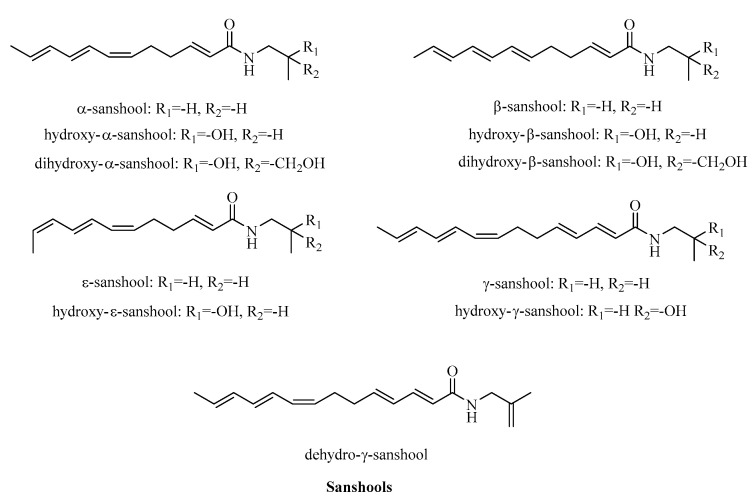
Chemical structures of sanshools extracted from Szechuan pepper fruits.

Sanshools are *N*-alkyl substituted compounds extracted from the *Zanthoxylum* species. Sanshools are well-known as spices and have several essential physiological and pharmacological functions. Sanshools and hydroxyl-sanshools are TRPV1 (a thermosensitive transient receptor potential ion channel vanilloid) agonists in sensory neurons, exhibiting pungent quality [32,33]. TRPV1 also shows an inhibition effect on an NF1- and p53-deficient mouse glioma cell line, indicating their potential antitumor activity [34]. Especially, hydroxy-α-sanshool can enhance the activity of the cholinergic system and increase the CREB/BDNF signaling pathway to attenuate scopolamine-induced cognitive impairments [35]. Besides, hydroxy-α-sanshool may also block KCNK9 channels to induce colonic motor activity in the rat proximal colon [36]. Naturally occurring products provide an excellent basis for the discovery of novel pesticides, including insecticides, fungicides, herbicides, and even herbicide safeners [18,25,37,38]. In our previous study, a mixture of sanshools consisting of hydroxy-α/β/ε-sanshool, α/β/ε-sanshool, γ-sanshool, hydroxyl-γ-sanshool, dehydro-γ-sanshool, and dihydroxy-α/β-sanshool (Figure 1) was extracted from Szechuan pepper fruits by the supercritical fluid extraction (SFE) method. Interestingly, the mixture demonstrated good herbicide safener activity for metolachlor in rice seedlings. However, the use of SFE to extract large quantities of sanshools is not cost-effective. Meanwhile, the reported procedures for organic synthesis of some of these sanshools are relatively complicated, mainly because of the construction of unsaturated long alkyl carbon chains with specific double-bond configurations in the carbon terminal of the amide bonds [39,40,41,42,43]. These factors have further limited the application of sanshools as herbicide safeners, indicating that their structures need modification (including simplification of the structures).

**Scheme 1:**
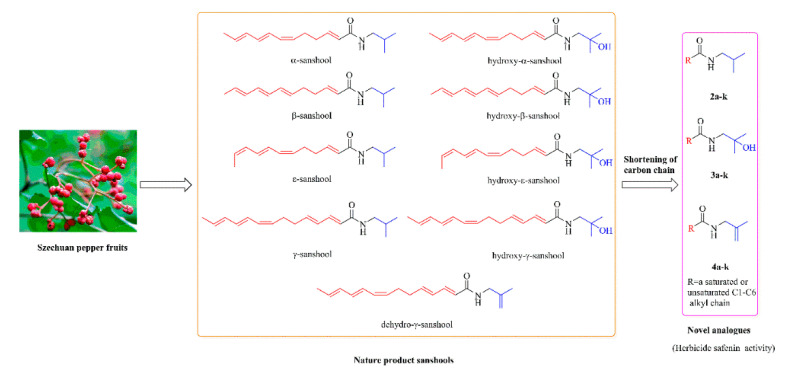
The design strategy of target compounds **2a**–**k**, **3a**–**k,** and **4a–k**.

Scaffold hopping is a useful tool for screening new pesticides used in pharmaceutical chemistry [44]. Among all scaffold hopping approaches, chain shortening is feasible for obtaining derivatives/analogs with improved potency by shortening or elongating flexible aliphatic chains (usually between two rings in the pesticide molecules). It is supposed that hydroxy-α/β/ε-sanshool, α/β/ε-sanshool, γ-sanshool, hydroxyl-γ-sanshool, and dehydro-γ-sanshool could be considered as the main compounds to design a series of sanshool derivatives (*N*-alkyl substituted analogs). The objective of the study was to simplify the structures of sanshools by replacing the unsaturated long alkyl carbon chains with saturated and unsaturated alkyl carbon chains with one to six carbon atoms, using the chain shortening method (Scheme 1). These compounds were synthesized via the one-spot method, and their structures were characterized by proton nuclear magnetic resonance (^1^H NMR), carbon 13 nuclear magnetic resonance (^13^C NMR), and high-performance liquid chromatography spectrum (HRMS). Subsequently, we evaluated their safener activities that alleviate metolachlor injury on rice seedlings, the action mechanism, and acute toxicity (96h) in zebrafish (*Danio rerio*) embryos, using dichlormid, an amide-type commercial herbicide safener, as the control. The results of this research could guide the design of new herbicide safeners with high efficacy from natural products and lay the foundation for developing safeners that are eco-friendly to aquatic organisms in rice fields.

## 2. Results and Discussion

### 2.1. Synthesis and Characterization of Target Compounds ***2a**–**k***, ***3a**–**k***, and ***4a**–**k***

**Scheme 2:**
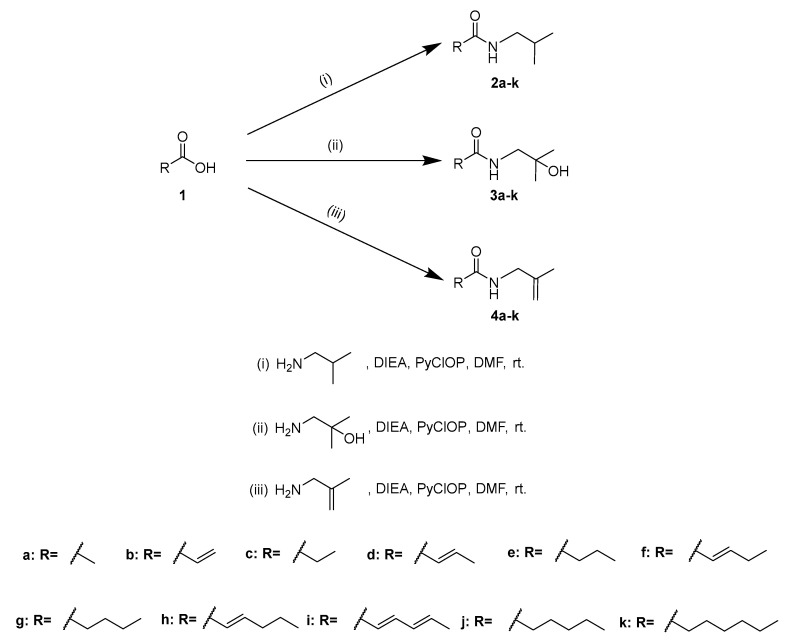
The synthetic procedures of target compounds **1a**–**k**, **2a**–**k**, and **3a**–**k**.

The one-spot condensation reaction was employed to obtain 43–89% yields of the target compounds **2a**–**k**, **3a**–**k**, and **4a**–**k**, using substituted acids and amines as starting materials and chlotripyrrolidinophosphonium hexafluophosphate (PyClOP) as a catalyst (Scheme 2) [45].

The structures of all the target compounds were well characterized by ^1^H NMR, ^13^C NMR, and HRMS spectroscopies. The ^1^H NMR spectrum of all the target compounds exhibited the hydrogen protons of amide bonds as singlets at 5.72–7.64 ppm and protons of hydroxyl moieties of **3a**–**k** as singlets at 2.59–4.44 ppm. Besides, the ^13^C NMR spectrum of all the target compounds showed the resonance of amide bonds (carbonyl moiety) at 162.2–173.8 ppm. What’s more, HRMS data of all the target compounds concurred with the calculated data based on chemical formula.

### 2.2. The Phytotoxicity of Target Compounds ***2a**–**k***, ***3a**–**k***, ***4a**–**k***, and Dichlormid on Rice Seedlings

Results on the phytotoxicities of 8 mg/L of **2a**–**k**, **3a**–**k**, **4a**–**k,** and dichlormid on the growth of rice seedlings (plant height, root length, and fresh weight) are shown in Table 1. The experimental results on phytotoxicity were described as plant height, root length, and fresh weight. Compared to non-treated controls, the growth indices of the target compounds and the positive control dichlormid ranged from 81.7–99.4% for plant height, 78.2–99.2% for root length, and 84.0–97.4% for fresh weight. The phytotoxicity testing results on rice plants revealed that the target compounds and dichlormid had very low inhibitory effects on the growth of rice seedlings. 

### 2.3. Safening Effects and Structure-Activity Relationship of ***2a**–**k***, ***3a**–**k***, and ***4a**–**k*** on Rice Seedlings

As shown in Table 2, the herbicide safener activities of target compounds **2a**–**k**, **3a**–**k**, and **4a**–**k** protected rice seedlings from metolachlor injury. Metolachlor (**Mcl**) significantly inhibited the growth of 7-day-old rice seedlings; the plant height, root length, and fresh weight were inhibited by 43.6%, 56.1%, and 75.0%, respectively. Treatments with target compounds **2a**–**k**, **3a**–**k**, and **4a**–**k** alleviated rice seedlings injury caused by metolachlor to different extents, ranging from 42.8–84.3%, 56.4–83.0%, and 76.8–87.9% for plant height, root length, and fresh weight, respectively. Unfortunately, the safener activities of **2a**–**k**, **3a**–**k**, and **4a**–**k** were less than those of dichlormid. However, the safener activities of **2k** and **4k** were very similar to that of dichlormid, with plant height relative values of 84.3% and 81.0%, respectively. Further, the root length of compound **3h** (83.0%) was very close to that of dichlormid. On the other hand, **3h** exhibited the best safener activity on fresh weight (87.9%).

A brief structure-activity relationship (SAR) of **2a**–**k**, **3a**–**k**, and **4a**–**k** was concluded as follows. When the R groups of all the compounds were the same, most of the compounds **2a**–**k** with *N*-substituted isobutyl group were generally more active (plant height activity) than **3a**–**k** with 2-hydroxy-2-methyl propyl groups and **4a**–**k** with 2-methyl allyl groups. In particular, with an increase in the length of the carbon chain, the biological activity generally showed an upward trend compared to **2a**–**k** and **4a**–**k**, when R groups represent saturated carbon chains. **2k** and **4k** with the longest carbon chain (six carbons) substitution showed the best safener activity on plant height. On the contrary, the activity of **3a**–**k** on plant height was not affected by the length of the carbon chain. Similar to the activity on plant length, compounds bearing the longest carbon chain (six carbons) moieties also exhibited the best activity on root length, compared to **2a**–**k** and **4a**–**k**. However, **3b** with (1*E*)-1-pentenyl (two carbons) was more active compared to **3a**–**k**. It should, however, be mentioned that when the number of carbon atoms in the main carbon chain is the same as in the R group, the saturation of the carbon chain affects the herbicide safener activity. On plant height, for instance, *n*-amyl substitution showed better or similar activity, compared to the (1*E*)-1-pentenyl and (1*E*, 3*E*)-1,3-pentadienyl substitutions. However, on root length and fresh weight, (1*E*)-1-pentenyl substitution showed less or very similar activity to that of *n*-amyl and (1*E*, 3*E*)-1,3-pentadienyl substitutions.

It was apparent that the herbicide safener activity was influenced by various attributes of the substitution groups, particularly the carbon length and degrees of saturation. Generally, compounds bearing a long carbon length moiety showed higher safener activities. In all, among all the target compounds, **2k** and **4k**, both with *n*-hexyl moieties and a different *N*-substitution (isobutyl moiety and 2-methyl allyl moiety, respectively), showed the best activity on plant height and excellent activity on root length and fresh weight. These two compounds (**2k** and **4k**) could, therefore, be considered the most potential candidates.

### 2.4. Safening Effect of Compounds ***2k***, ***4k***, and Dichlormid in Rice at Lower Concentrations

**Figure 2:**
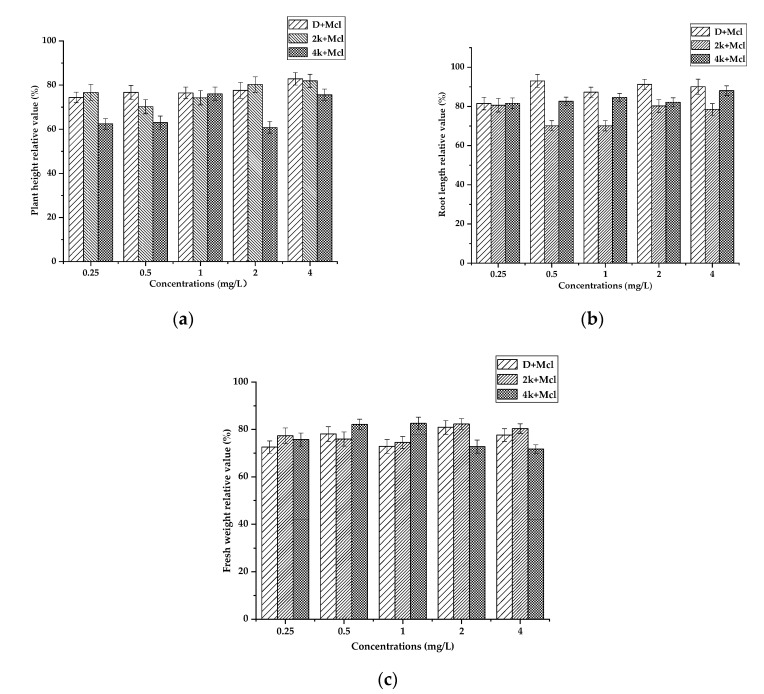
Plant height (**a**), root length (**b**), and fresh weight (**c**) relative values of 0.25 μM metolachlor (**Mcl**)-treated rice plants treated with compounds **2k**, **4k**, and dichlormid (**D**) at 0.25 mg/L, 0.5 mg/L, 1 mg/L, 2 mg/L, and 4 mg/L, respectively. The values present the means of three replicates ± SE of each mean.

To verify the safening effects of **2k** and **4k**, their herbicide safener activities were further tested at lower concentrations. As shown in Figure 2, there was not an explicit relationship between the concentrations and the safening effects of **2k**, **4k**, and dichlormid on plant height, root length, and fresh weight. Although dichlormid showed better activity than both **2k** and **4k** on both plant length and root length at most concentrations, the activities of **2k** and **4k** were still substantial at some concentrations, for example, 1 mg/L and 4 mg/L for plant height and 0.25 mg/L, 2 mg/L, and 4 mg/L for root length (Figure 2a,b). For fresh weight, **2k** was more active than **4k** and dichlormid at 0.25 mg/L, 2 mg/L, and 4 mg/L, while **4k** showed the best safener activities at 0.5 mg/L and 1 mg/L (Figure 2c). These results also indicated that **2k** and **4k** showed moderate to good safener activities, even at lower concentrations.

### 2.5. Enzyme Activity Assay

**Figure 3:**
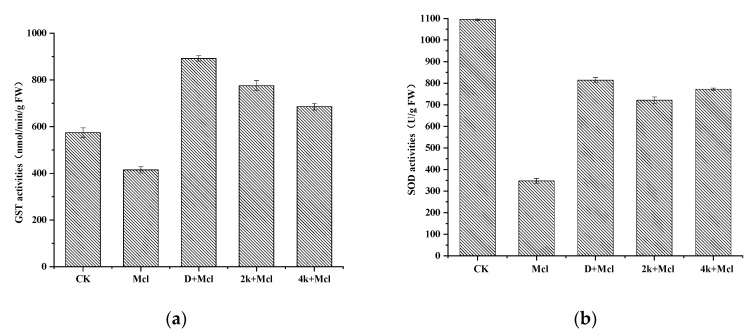

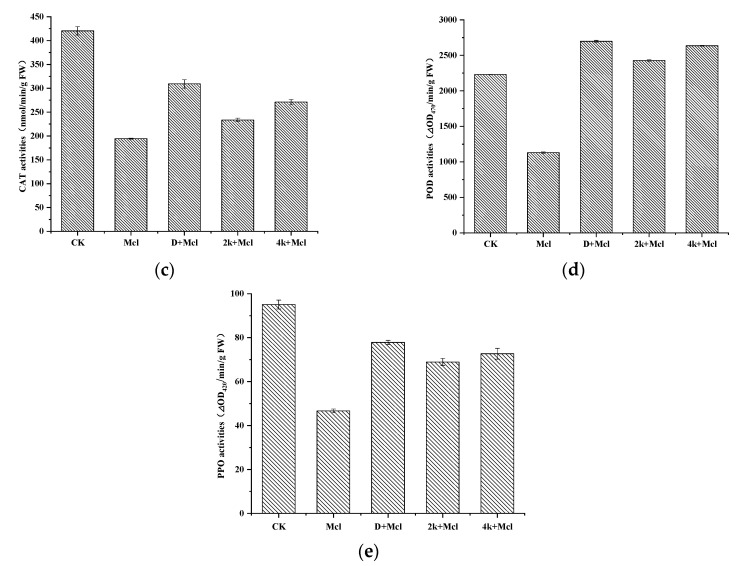
Compounds **2k, 4k,** and dichlormid on enzyme activities of rice seedlings. (**a**) GST, (**b**) SOD, (**c**) CAT, (**d**) POD, (**e**) PPO. The values present the means of three replicates ± SE of each mean. GST, glutathione S-transferase; SOD, superoxide dismutase; CAT, catalase; POD, peroxidase; PPO, polyphenol oxidases.

GST can catalyze the conjugation of GSH and herbicide molecules in crop plants, which subsequently improves the tolerance of crop plants to herbicides [46]. As shown in Figure 3a, GST activity decreased significantly after the treatment of metolachlor on rice seedlings. However, the results of the present study demonstrated that the GST activities of rice seedlings treated with a combination of compounds **2k** and **4k** with metolachlor were greater than that of rice seedlings treated with metolachlor alone and even higher than that of control check (CK). Except for dichlormid, rice treated with compound **2k** exhibited the best GST activity, which was almost 187% of the metolachlor-treated group and 1.16 times of CK.

To minimize toxicity caused by herbicides, crop plants have evolved their own protective mechanisms, for instance, the antioxidant system that is comprised of a series of enzymes, such as superoxide dismutase (SOD), peroxidase (POD), and catalase (CAT) [47,48,49]. These antioxidant enzyme activities have also been reported to be associated with herbicide tolerance in crop plants [28]. When rice seedlings were only treated by metolachlor, SOD, POD, and CAT activities decreased significantly, compared to that of CK (Figure 3b–d). After the addition of compounds **2k** and **4k**, the SOD, POD, and CAT activities increased dramatically. For **2k** treatments, the SOD, POD, and CAT activities increased by 208%, 120%, and 215%, respectively; for **4k** treatments, the SOD, POD, and CAT activities increased by 223%, 140%, and 233%, respectively. These results indicated that **2k** and **4k** could induce antioxidant enzyme activities that were inhibited by metolachlor.

Polyphenol oxidases (PPOs) are peroxidases involved in the biosynthesis of black/brown pigment and have played protective roles in crop plants against fungus, environmental stress, and herbicide stress [50,51]. The results showed that metolachlor inhibited the PPO activity of rice seedlings, which was extremely unfavorable for rice growth (Figure 3e). In rice seedlings treated with compounds **2k** and **4k**, the PPO activities increased by 147% and 155%, respectively, compared to metolachlor treatments. Thus, these target compounds **2k** and **4k** effectively alleviated the inhibition of PPO activity by herbicide metolachlor.

### 2.6. Acute Toxicity of Compounds ***2k***, ***4k***, and Dichlormid on Zebrafish Embryos

As dichlormid has been reported to pose potential risks to aquatic organisms, such as fish, we further evaluated the acute toxicity (96 h) of **2k**, **4k**, and dichlormid on zebrafish embryo; a model frequently used to evaluate the toxicity of various compounds to aquatic organisms [52,53]. Dichlormid displayed moderate acute toxicity (7.86 mg/L) to zebrafish embryos, while **2k** and **4k** displayed very low acute toxicities, with LC_50_ (Lethal Concentration 50%) values of 38.29 mg/L and 39.28 mg/L, respectively (Table 3). These results indicated that **2k** and **4k** were safer for aquatic organisms relative to dichlormid. Besides, we would test the effects of **2k** and **4k** on other additional environmental factors, such as soil factor, under real farm conditions in future work.

## 3. Materials and Methods

### 3.1. Chemicals

Metolachlor (emulsifiable concentrate, 960 g/L), substituted fatty acids (purity 97–99%), isobutylamine (purity 99%), 2-methyl allylamine (purity 97%), 1-amino-2-methyl propan-2-ol (purity 98%), chlorotripyrrolidinophosphonium hexafluorophosphate (PyClOP, purity 98%), and *N*,*N*-diisopropylethylamine (DIEA, 99%) were purchased from Jilin Chinese Academy of Sciences-Yanshen Technology Co., Ltd. (Jilin, Changchun, China). All solvents were bought from Sinopharm Chemical Reagent Co., Ltd. (Beijing, China) and further distilled before use. Thin-layer chromatography (TLC) was performed with Silicagel GF254 (Merck KGaA, Darmstadt, Germany). The isolation and purification were carried out via silica gel column chromatography (200 mesh). Melting points (Mp) were measured by a Hanon MP100 automatic melting point apparatus (Jinan Hanon Instruments Co., Ltd., Jinan, China) and autocorrected. ^1^H NMR and ^13^C NMR were recorded on a Bruker Avance-300 spectrometer operating at 300 MHz (^1^H) and 75 MHz (^13^C), respectively, using tetramethylsilane (TMS) as an internal standard (solvent CDCl_3_ or DMSO-*d*_6_). Chemical shifts (*δ*) were reported in parts per million (ppm), and coupling constants (*J*) were in Hz. HRMS was analyzed on a Varian 7.0 T FTICR-MS instrument (Varian IonSpec, Lake Forest, CA, USA). 

### 3.2. General Procedures for the Synthesis of N-Alkyl Amides ***2a**–**k***, ***3a**–**k***, and ***4a**–**k***

*N*-alkyl amides **2a**–**k**, **3a**–**k**, and **4a**–**k** were synthesized using the method reported in the previous reference. The PyClOP (10 mmol) was added to a solution of carboxylic acid **1a**–**k** (10 mmol), amine (butylamine, 2-methyl allylamine, or 1-amino-2-methyl propan-2-ol, 10 mmol), and DIEA (20 mmol) in 30 mL of dimethylformamide (DMF) at 0 °C. Next, the mixture solution was continuously stirred for 12 h at room temperature and then diluted with 20 mL water and 20 mL ethyl acetate. The organic layer was washed with 100 mL brine twice, 100 mL dilute HCl (1 mol/L) twice, 100 mL water twice, 100 mL 5% NaHCO_3_ twice, and 100 mL brine twice. The resulting mixture solution was concentrated under vacuum and further purified by flash chromatography on a silica gel column (ethyl acetate: petroleum ether, 1:6) to afford the *N*-alkyl amides **2a**–**k**, **3a**–**k,** and **4a**–**k** (yields 34–84%) as colorless oils, yellow oils, white solids, or yellow solids. ^1^H NMR and ^13^C NMR spectrums of target compounds **2a**–**k**, **3a**–**k**, and **4a**–**k** see the Supplementary Materials.

*N*-isobutylacetamide (**2a**). Yellow liquid; Yield 58%; ^1^H NMR (300 MHz, CDCl_3_): *δ* 5.73 (s, 1H, NH), 3.03–3.07 (m, 2H, CH_2_), 1.97 (s, 3H, CH_3_), 1.68–1.81 (m, 1H, CH), 0.89 (d, *J* = 6.0 Hz, 6H, CH_3_); ^13^C NMR (75 MHz, CDCl_3_): *δ* 170.3, 46.9, 28.3, 23.1, 19.9, 19.7; HRMS calcd. for C_6_H_13_NO ([M + H]^+^), 116.1070; found, 116.1071.

*N*-isobutylacrylamide (**2b**). Yellow liquid; Yield 53%; ^1^H NMR (300 MHz, CDCl_3_): *δ* 6.89 (s, 1H, NH), 6.19 (d, *J* = 1.6 Hz, 1H, CH), 6.17 (s, 1H, CH), 5.52 (dd, *J* = 6.8, 5.2 Hz, 1H, CH), 3.05 (d, *J* = 13.0 Hz, 2H, CH_2_), 1.74 (dp, *J* = 13.4, 6.7 Hz, 1H, CH), 0.84 (d, *J* = 6.7 Hz, 6H, CH_3_); ^13^C NMR (75 MHz, CDCl_3_): *δ* 165.7, 131.0, 125.8, 46.9, 28.4, 20.0; HRMS calcd. for C_7_H_13_NO ([M + H]^+^), 128.1070; found, 128.1069.

*N*-isobutylpropionamide (**2c**). White liquid; Yield 34%; ^1^H NMR (300 MHz, CDCl_3_): *δ* 6.04 (s, 1H, NH), 3.00 (t, *J* = 6.4 Hz, 2H, CH_2_), 2.16 (q, *J* = 7.6 Hz, 2H, CH_2_), 1.71 (dq, *J* = 13.5, 6.7 Hz, 1H, CH), 1.09 (t, *J* = 7.6 Hz, 3H, CH_3_), 0.84 (dd, *J* = 6.7 Hz, 6H, CH_3_); ^13^C NMR (75 MHz, CDCl_3_): *δ* 172.7, 45.9, 28.4, 28.0, 19.9, 9.9; HRMS calcd. for C_7_H_15_NO ([M + H]^+^), 130.1226; found, 130.1225. 

(*E*)*-N*-isobutylbut-2-enamide (**2d**). White solid; Mp 67.5–69.5 °C; Yield 51%; ^1^H NMR (300 MHz, CDCl_3_): *δ* 6.83 (dq, *J* = 15.1, 6.9 Hz, 1H, CH), 5.79 (dq, *J* = 15.2, 1.7 Hz, 1H, CH), 5.48 (s, 1H, NH), 3.14 (dd, *J* = 6.8, 6.1 Hz, 2H, CH_2_), 1.84 (dd, *J* = 6.9, 1.7 Hz, 3H, CH_3_), 1.76 (dt, *J* = 13.4, 6.7 Hz, 1H, CH), 0.91 (d, *J* = 6.7 Hz, 6H, CH_3_); ^13^C NMR (75 MHz, CDCl_3_): *δ* 166.1, 139.2, 125.2, 46.7, 28.5, 20.0, 17.5; HRMS calcd. for C_8_H_15_NO ([M + H]^+^), 142.1226; found, 142.1225.

*N*-isobutylbutyramide (**2e**). White liquid; Yield 57%; ^1^H NMR (300 MHz, CDCl_3_): *δ* 5.78 (s, 1H, NH), 3.04–3.12 (m, 2H, CH_2_), 2.17 (dd, *J* = 8.2, 6.8 Hz, 2H, CH_2_), 1.61–1.81 (m, 3H, CH + CH_2_), 0.88–0.98 (m, 9H, CH_3_); ^13^C NMR (75 MHz, CDCl_3_): *δ* 171.7, 45.8, 37.3, 28.0, 19.9, 18.7, 13.4; HRMS calcd. for C_8_H_17_NO ([M + H]^+^), 144.1383; found, 144.1381.

(*E*)*-N*-isobutylpent-2-enamide (**2f**). Yellow solid; Mp 41.8–42.5 °C; Yield 72%; ^1^H NMR (300 MHz, CDCl_3_): *δ* 6.87 (m, *J* = 15.3, 6.4 Hz, 1H, CH), 5.82 (m, *J* = 1.7 Hz, 1H, CH), 5.77 (t, *J* = 1.7 Hz, 1H, NH), 3.14 (dd, *J* = 6.0, 6.1 Hz, 2H, CH_2_), 2.19 (ddd, *J* = 7.5, 6.4, 1.7 Hz, 2H, CH_2_), 1.72–1.90 (m, 1H, CH), 1.05 (t, *J* = 7.4 Hz, 3H, CH_3_), 0.92 (d, *J* = 6.7 Hz, 6H, CH_3_); ^13^C NMR (75 MHz, CDCl_3_): *δ* 166.3, 145.6, 122.8, 46.8, 28.5, 25.0, 20.1, 12.4; HRMS calcd. for C_9_H_17_NO ([M + H]^+^), 156.1383; found, 156.1382.

*N*-isobutylpentanamide (**2g**). White liquid; Yield 80%; ^1^H NMR (300 MHz, CDCl_3_): *δ* 5.73 (s, 1H, NH), 3.05–3.10 (m, 2H, CH_2_), 2.16–2.21 (m, 2H, CH_2_), 1.72–1.81 (m, 1H, CH), 1.57–1.67 (m, 2H, CH_2_), 1.35 (dd, *J* = 15.0, 7.4 Hz, 2H, CH_2_), 0.90–0.94 (m, 9H, CH_3_); ^13^C NMR (75 MHz, CDCl_3_): *δ* 171.9, 45.8, 35.0, 28.0, 27.5, 21.7, 20.0, 13.6; HRMS calcd. for C_9_H_19_NO ([M + H]^+^), 158.1539; found, 158.1538.

(*E*)*-N*-isobutylhex-2-enamide (**2h**). White liquid; Yield 83%; ^1^H NMR (300 MHz, CDCl_3_): *δ* 6.79 (dt, *J* = 15.2, 7.0 Hz, 1H, CH_2_), 5.94 (s, 1H, NH), 5.79 (dt, *J* = 15.3, 1.5 Hz, 1H, CH), 3.11 (dd, *J* = 6.8, 6.2 Hz, 2H, CH_2_), 2.16–2.06 (m, 2H, CH_2_), 1.76 (dq, *J* = 13.4, 6.7 Hz, 1H, CH_2_), 1.44 (h, *J* = 7.3 Hz, 2H, CH_2_), 0.95–0.80 (m, 9H, CH_3_); ^13^C NMR (75 MHz, CDCl_3_): *δ* 166.2, 144.2, 123.8, 46.7, 34.0, 28.5, 21.4, 20.1, 13.6; HRMS calcd. for C_10_H_19_NO ([M + H]^+^), 170.1539; found, 170.1538.

(*2E*, *4E*)*-N*-isobutylhexa-2, 4-dienamide (**2i**). White solid; Mp 106.5–107.0 °C; Yield 81%; ^1^H NMR (300 MHz, CDCl_3_): *δ* 7.18 (dd, *J* = 14.9, 9.9 Hz, 1H, CH), 6.01–6.19 (m, 2H, CH), 5.73 (d, *J* = 15.0 Hz, 1H, CH), 5.46 (s, 1H, NH), 3.14–3.18 (m, 2H, CH_2_), 1.82–1.84 (m, 4H, CH + CH_3_), 0.92 (d, *J* = 6.7 Hz, 6H, CH_3_); ^13^C NMR (75 MHz, CDCl_3_): *δ* 166.5, 140.7, 137.2, 129.7, 121.9, 46.9, 28.5, 20.1, 18.4; HRMS calcd. for C_10_H_17_NO ([M + H]^+^), 168.1383; found, 168.1381.

*N*-isobutylhexanamide (**2j**). White liquid; Yield 84%; ^1^H NMR (300 MHz, CDCl_3_): *δ* 5.88 (s, 1H, NH), 3.07 (dd, *J* = 6.9, 6.0 Hz, 2H, CH_2_), 2.16–2.21 (m, 2H, CH_2_), 1.78 (dq, *J* = 13.4, 6.7 Hz, 1H, CH), 1.62–1.69 (m, 2H, CH_2_), 1.26–1.35 (m, 4H, CH_2_), 0.86–0.96 (m, 9H, CH_3_); ^13^C NMR (75 MHz, CDCl_3_): *δ* 171.9, 45.8, 35.3, 30.8, 28.2, 28.0, 25.0, 21.8, 20.0, 13.8; HRMS calcd. for C_10_H_21_NO ([M + H]^+^), 172.1696; found, 172.1694.

*N*-isobutylheptanamide (**2k**). White liquid; Yield 63%; ^1^H NMR (300 MHz, CDCl_3_): *δ* 5.83 (s, 1H, NH), 3.06 (t, *J* = 6.4 Hz, 2H, CH_2_), 2.13–2.22 (m, 2H, CH_2_), 1.76 (tq, *J* = 13.4, 6.7 Hz, 1H, CH), 1.57–1.67 (m, 2H, CH_2_), 1.28–1.33 (m, 6H, CH_2_), 0.85–0.92 (m, 9H, CH_3_); ^13^C NMR (75 MHz, DMSO-*d*_6_) *δ* 171.9, 45.8, 35.3, 31.0, 28.3, 28.0, 25.3, 21.9, 20.0, 13.8; HRMS calcd. for C_11_H_23_NO ([M + H]^+^), 186.1852; found, 186.1842.

*N*-(2-hydroxy-2-methylpropyl)acetamide (**3a**). Colorless liquid; Yield 57%; ^1^H NMR (300 MHz, CDCl_3_): *δ* 6.29 (s, 1H, NH), 3.25 (d, *J* = 6.0 Hz, 2H, CH_2_), 2.98 (s, 1H, OH), 2.03 (s, 3H, CH_3_), 1.22 (s, 6H, CH_3_); ^13^C NMR (75 MHz, CDCl_3_): *δ* 171.1, 70.7, 50.4, 27.1, 23.0; HRMS calcd. for C_6_H_13_NO_2_ ([M + H]^+^), 132.1019; found, 132.1018.

*N*-(2-hydroxy-2-methylpropyl)acrylamide (**3b**). Colorless liquid; Mp 67.5–68.2 °C; Yield 54%; ^1^H NMR (300 MHz, CDCl_3_): *δ* 6.50 (s, 1H, NH), 6.12–6.33 (m, 2H, CH_2_), 5.66 (dd, *J* = 9.9, 1.8 Hz, 1H, CH), 3.34 (d, *J* = 6.1 Hz, 2H, CH_2_), 3.09 (s, 1H, OH), 1.23 (s, 6H, CH_3_); ^13^C NMR (75 MHz, CDCl_3_): *δ* 166.6, 130.6, 126.7, 70.8, 50.4, 27.2; HRMS calcd. for C_7_H_13_NO_2_ ([M + H]^+^), 144.1019; found, 144.1018.

*N*-(2-hydroxy-2-methylpropyl)propionamide (**3c**). Colorless liquid; Yield 61%; ^1^H NMR (300 MHz, CDCl_3_): *δ* 7.61 (s, 1H, NH), 4.44 (s, 1H, OH), 3.00 (d, *J* = 6.1 Hz, 2H, CH_2_), 2.11 (q, *J* = 7.6 Hz, 2H, CH_2_), 1.02 (s, 6H, CH_3_), 0.98 (t, *J* = 7.6 Hz, 3H, CH_3_); ^13^C NMR (75 MHz, CDCl_3_): *δ* 173.8, 70.0, 50.1, 29.0, 27.7, 10.7; HRMS calcd. for C_7_H_15_NO_2_ ([M + H]^+^), 146.1176; found, 146.1173.

(*E*)*-N*-(2-hydroxy-2-methylpropyl)but-2-enamide (**3d**). Colorless liquid; Yield 47%; ^1^H NMR (300 MHz, DMSO-*d*_6_): *δ* 6.86 (dq, *J* = 15.2, 6.9 Hz, 1H, CH), 5.94 (s, 1H, NH), 5.81–5.88 (m, 1H, CH), 3.32 (d, *J* = 6.1 Hz, 2H, CH_2_), 2.59 (s, 1H, OH), 1.86 (dd, *J* = 6.9, 1.7 Hz, 3H, CH_3_), 1.23 (s, 6H, CH_3_); ^13^C NMR (75 MHz, CDCl_3_): *δ* 167.1, 140.2, 124.8, 70.8, 50.4, 27.1, 17.6; HRMS calcd. for C_8_H_15_NO_2_ ([M + H]^+^), 158.1176; found, 158.1175.

*N*-(2-hydroxy-2-methylpropyl)butyramide (**3e**). White solid; Mp 38.9–39.2 °C; Yield 56%; ^1^H NMR (300 MHz, DMSO-*d*_6_): *δ* 7.64 (s, 1H, NH), 4.44 (s, 1H, OH), 3.01 (d, *J* = 6.1 Hz, 2H, CH_2_), 2.08 (t, *J* = 6.0 Hz, 2H, CH_2_), 1.50 (h, *J* = 7.3 Hz, 2H, CH_2_), 1.03 (s, 6H, CH_3_), 0.84 (t, *J* = 7.4 Hz, 3H, CH_3_); ^13^C NMR (75 MHz, CDCl_3_): *δ* 172.9, 100.0, 70.0, 50.1, 27.7, 19.3, 14.2; HRMS calcd. for C_8_H_17_NO_2_ ([M + H]^+^), 160.1332; found, 160.1331.

(*E*)*-N*-(2-hydroxy-2-methylpropyl)pent-2-enamide (**3f**). Yellow liquid; Yield 61%; ^1^H NMR (300 MHz, CDCl_3_): *δ* 6.88–6.93 (m, 1H, CH), 6.09 (s, 1H, NH), 5.81 (d, *J* = 15.3 Hz, 1H, CH), 3.32 (d, *J* = 6.1 Hz, 2H, CH_2_), 2.85 (s, 1H, OH), 2.18–2.23 (m, 2H, CH_2_), 1.22 (s, 6H, CH_3_), 1.05 (t, *J* = 7.4 Hz, 3H, CH_3_); ^13^C NMR (75 MHz, CDCl_3_): *δ* 162.2, 141.3, 117.2, 65.5, 45.3, 21.9, 19.8, 7.1; HRMS calcd. for C_9_H_17_NO_2_ ([M + H]^+^), 172.1332; found, 172.1330.

*N*-(2-hydroxy-2-methylpropyl)pentanamide (**3g**). White solid; Mp 64.2–65.2 °C; Yield 43%; ^1^H NMR (300 MHz, DMSO-*d*_6_): *δ* 7.64 (s, 1H, NH), 4.43 (s, 1H, OH), 3.01 (d, *J* = 6.0 Hz, 2H, CH_2_), 2.11 (t, *J* = 7.4 Hz, 2H, CH_2_), 1.47 (p, *J* = 7.4 Hz, 2H, CH_2_), 1.22–1.29 (m, 2H, CH_2_), 1.02 (s, 6H, CH_3_), 0.85 (t, *J* = 7.4 Hz, 3H, CH_3_); ^13^C NMR (75 MHz, CDCl_3_): *δ* 173.0, 70.0, 50.1, 35.6, 28.1, 27.7, 22.3, 14.3; HRMS calcd. for C_9_H_19_NO_2_ ([M + H]^+^), 174.1489; found, 174.1487.

(*E*)-*N*-(2-hydroxy-2-methylpropyl)hex-2-enamide (**3h**). White solid; Mp 41.3–42.0 °C; Yield 43%; ^1^H NMR (300 MHz, CDCl_3_): *δ* 6.79–6.89 (dt, *J* = 15.3, 7.0 Hz, 1H, CH), 6.41 (s, 1H, NH), 5.82–5.88 (d, *J* = 15.3 Hz, 1H, CH), 3.33 (s, 2H, CH_2_), 3.30 (s, 1H, OH), 2.15 (q, *J* = 7.3 Hz, 2H, CH_2_), 1.40–1.52 (m, 2H, CH_2_), 1.22 (s, 6H, CH_3_), 0.90 (t, *J* = 7.4 Hz, 3H, CH_3_); ^13^C NMR (75 MHz, CDCl_3_): δ 167.2, 145.0, 123.3, 70.8, 50.4, 34.0, 27.1, 21.4, 13.6; HRMS calcd. for C_10_H_19_NO_2_ ([M + H]^+^), 186.1489; found, 186.1488.

(*2E*,*4E*)-*N*-(2-hydroxy-2-methylpropyl)hexa-2,4-dienamide (**3i**). White solid; Mp 142.2–142.7 °C; Yield 41%; ^1^H NMR (300 MHz, CDCl_3_): *δ* 7.20 (dd, *J* = 15.0, 9.8 Hz, 1H, CH), 6.03–6.20 (m, 3H, CH+NH), 5.77 (d, *J* = 15.0 Hz, 1H, CH), 3.34 (d, *J* = 6.1 Hz, 2H, CH_2_), 2.82 (s, 1H, OH), 1.83 (d, *J* = 5.7 Hz, 3H, CH_3_), 1.23 (s, 6H, CH_3_); ^13^C NMR (75 MHz, CDCl_3_): *δ* 167.6, 141.5, 138.0, 129.6, 121.2, 70.8, 50.5, 27.1, 18.5; HRMS calcd. for C_10_H_17_NO_2_ ([M + H]^+^), 184.1332; found, 184.1331.

*N*-(2-hydroxy-2-methylpropyl)hexanamide (**3j**). White solid; Mp 53.8–54.3 °C; Yield 52%; ^1^H NMR (300 MHz, DMSO-*d*_6_): *δ* 7.64 (m, 1H, NH), 4.43 (s, 1H, OH), 3.00 (d, *J* = 6.1 Hz, 2H, CH_2_), 2.10 (t, *J* = 7.4 Hz, 2H, CH_2_), 1.49 (p, *J* = 7.4 Hz, 2H, CH_2_), 1.18–1.29 (m, 4H, CH_2_), 1.02 (s, 6H, CH_3_), 0.85 (t, *J* = 7.1 Hz, 3H, CH_3_); ^13^C NMR (75 MHz, DMSO-*d*_6_): *δ* 173.0, 70.0, 50.1, 35.8, 31.4, 27.7, 25.6, 22.4, 14.4; HRMS calcd. for C_10_H_21_NO_2_ ([M + H]^+^), 188.1645; found, 188.1643.

*N*-(2-hydroxy-2-methylpropyl)heptanamide (**3k**). White liquid; Yield 43%; ^1^H NMR (300 MHz, DMSO-*d*_6_): δ 7.64 (s, 1H, NH), 4.43 (s, 1H, OH), 3.37 (s, 2H, CH_2_), 3.00 (d, *J* = 6.1 Hz, 2H, CH_2_), 2.10 (t, *J* = 6.0 Hz, 2H, CH_2_), 1.49 (p, *J* = 7.4 Hz, 2H, CH_2_), 1.24 (dqd, *J* = 22.7, 7.9, 7.4, 2.0 Hz, 4H, CH_2_), 1.02 (s, 6H, CH_3_), 0.84 (t, *J* = 7.1 Hz, 3H, CH_3_); ^13^C NMR (75 MHz, DMSO-*d*_6_): *δ* 173.0, 70.0, 50.1, 35.8, 31.4, 27.7, 25.6, 22.4, 14.4; HRMS calcd. for C_11_H_23_NO_2_ ([M + H]^+^), 202.1802; found, 202.1800.

*N*-(2-methylallyl)acetamide (**4a**). Yellow liquid; Yield 56%; ^1^H NMR (300 MHz, CDCl_3_): *δ* 5.80 (s, 1H, NH), 4.82–4.84 (s, 2H, CH_2_), 3.80 (d, *J* = 6.0 Hz, 2H, CH_2_), 2.02 (s, 3H, CH_3_), 1.73 (m, 3H, CH_3_); ^13^C NMR (75 MHz, CDCl_3_): *δ* 170.2, 141.7, 110.4, 44.8, 22.8, 20.1; HRMS calcd. for C_6_H_11_NO ([M+H]^+^), 114.0913; found, 114.0915.

*N*-(2-methylallyl)acrylamide (**4b**). White liquid; Yield 64%; ^1^H NMR (300 MHz, CDCl_3_): *δ* 6.31 (dd, *J* = 17.0, 1.7 Hz, 1H, CH), 6.15 (dd, *J* = 17.0, 10.1 Hz, 1H, CH_2_), 5.94 (s, 1H, NH), 5.66 (dd, *J* = 10.0, 1.7 Hz, 1H, CH), 4.85 (h, *J* = 1.1 Hz, 2H, CH_2_), 3.89 (d, *J* = 6.1 Hz, 2H, CH_2_), 1.75 (s, 3H, CH_3_); ^13^C NMR (75 MHz, CDCl_3_): *δ* 165.8, 141.6, 130.9, 126.1, 110.9, 45.0, 20.3; HRMS calcd. for C_7_H_11_NO ([M + H]^+^), 126.0913; found, 126.0912.

*N*-(2-methylallyl)propionamide (**4c**). Yellow liquid; Yield 69%; ^1^H NMR (300 MHz, CDCl_3_): *δ* 5.57 (s, 1H, NH), 4.81–4.83 (s. 2H, CH), 3.91 (d, *J* = 6.0 Hz, 2H, CH_2_), 2.24 (q, *J* = 7.6 Hz, 2H, CH_2_), 1.73 (s, 3H, CH_3_), 1.18 (t, *J* = 7.6 Hz, 3H, CH_3_); ^13^C NMR (75 MHz, CDCl_3_): *δ* 165.8, 141.6, 130.9, 126.1, 110.9, 45.0, 20.2; HRMS calcd. for C_7_H_13_NO ([M + H]^+^), 128.1070; found, 128.1070.

(*E*)*-N-*(*2-methylallyl*)*but-2-enamide* (**4d**). Yellow liquid; Yield 59%; ^1^H NMR (300 MHz, CDCl_3_): *δ* 6.87 (dq, *J* = 15.1, 6.9 Hz, 1H, CH), 5.84 (dq, *J* = 15.2, 1.7 Hz, 1H, CH), 5.62 (s, 1H, NH), 4.84 (p, *J* = 1.2 Hz, 2H, CH_2_), 3.88 (d, *J* = 6.1 Hz, 2H, CH_2_), 1.87 (dd, *J* = 6.9, 1.7 Hz, 3H, CH_3_), 1.75 (d, *J* = 1.1 Hz, 3H, CH_3_); ^13^C NMR (75 MHz, CDCl_3_): *δ* 166.0, 141.9, 139.7, 124.9, 110.7, 44.8, 20.2, 17.6; HRMS calcd. for C_8_H_13_NO ([M + H]^+^), 140.1070; found, 140.1068.

*N-*(*2-methylallyl*)*butyramide* (**4e**). Colorless liquid; Yield 54%; ^1^H NMR (300 MHz, CDCl_3_): *δ* 6.85–6.93 (m, 1H, CH), 5.79 (dt, *J* = 15.3, 1.7 Hz, 1H, CH), 5.62 (s, 1H, NH), 4.83 (p, *J* = 1.1 Hz, 2H, CH_2_), 3.88 (d, *J* = 6.1 Hz, 2H, CH_2_), 2.21 (ddd, *J* = 7.7, 6.4, 1.5 Hz, 2H, CH_2_), 1.74 (s, 3H, CH_3_), 1.06 (s, 3H, CH_3_); ^13^C NMR (75 MHz, CDCl_3_): *δ* 172.9, 141.9, 110.4, 44.7, 38.3, 20.2, 19.1, 13.6; HRMS calcd. for C_8_H_15_NO ([M + H]^+^), 142.1226; found, 142.1225. 

(*E*)*-N*-(2-methylallyl)pent-2-enamide (**4f**). Colorless liquid; Yield 53%; ^1^H NMR (300 MHz, CDCl_3_): *δ* 6.85–69.5 (m, 1H, CH), 5.62–5.82 (dt, *J* = 15.3, 1.7 Hz, 1H, CH), 5.62 (s, 1H, NH), 4.83 (q, *J* = 1.1 Hz, 2H, CH_2_), 3.88 (d, *J* = 6.1 Hz, 2H, CH_2_), 2.21 (ddd, *J* = 7.7, 6.4, 1.5 Hz, 2H, CH_2_), 1.04 (s, 3H, CH_3_), 1.06 (t, *J* = 7.4 Hz, 3H, CH_3_); ^13^C NMR (75 MHz, CDCl_3_): *δ* 166.2, 146.0, 141.9, 122.6, 110.7, 44.9, 25.0, 20.3, 12.4; HRMS calcd. for C_9_H_15_NO ([M + H]^+^), 154.1226; found, 154.1224.

*N*-(2-methylallyl)pentanamide (**4g**). Yellow liquid; Yield 61%; ^1^H NMR (300 MHz, CDCl_3_): *δ* 5.85 (s, 1H, NH), 4.92–4.78 (m, 2H, CH_2_), 3.81 (d, *J* = 6.0 Hz, 2H, CH_2_), 2.19–2.28 (m, 2H, CH_2_), 1.74 (d, *J* = 2.9 Hz, 3H, CH_3_), 1.57–1.70 (m, 2H, CH_2_), 1.30–1.44 (m, 2H, CH_2_), 0.93 (t, *J* = 7.3 Hz, 3H, CH_3_); ^13^C NMR (75 MHz, CDCl_3_): *δ* 173.2, 141.9, 110.3, 44.6, 36.2, 27.8, 22.2, 20.1, 13.6; HRMS calcd. for C_9_H_17_NO ([M + H]^+^), 156.1383; found, 156.1381.

(*E*)*-N*-(2-methylallyl)hex-2-enamide (**4h**). White solid; Mp 37.0–38.8 °C; Yield 57%; ^1^H NMR (300 MHz, CDCl_3_): *δ* 6.85 (dt, 1H, *J* = 15.3, 7.0 Hz, CH), 5.80 (dt, 1H, *J* = 15.3, 1.5 Hz, CH), 5.66 (s, 1H, NH), 4.83 (q, *J* = 1.2 Hz, 2H, CH_2_), 3.87 (d, *J* = 6.1 Hz, 2H, CH_2_), 2.15 (qd, *J* = 7.1, 1.5 Hz, 2H, CH_2_), 1.73–1.75 (m, 3H, CH_3_), 1.47 (h, *J* = 7.3 Hz, 2H, CH_2_), 0.92 (t, *J* = 7.4 Hz, 3H, CH_3_); ^13^C NMR (75 MHz, CDCl_3_): *δ* 166.0, 144.7, 142.0, 123.5, 110.8, 44.9, 34.0, 21.4, 20.3, 13.6; HRMS calcd. for C_10_H_17_NO ([M + H]^+^), 168.1383; found, 168.1381.

(*2E*, *4E*)*-N*-(2-methylallyl)hexa-2,4-dienamide (**4i**). Yellow solid; Mp 66.3–67.1 °C; Yield 49%; ^1^H NMR (300 MHz, CDCl_3_): *δ* 7.17–7.25 (m, 1H, CH), 6.03–6.21 (m, 2H, CH), 5.77 (d, *J* = 14.9 Hz, 1H, CH), 5.58 (s, 1H, NH), 4.84 (q, *J* = 1.2 Hz, 2H, CH_2_), 3.89 (d, *J* = 6.1 Hz, 2H, CH_2_), 1.83 (d, *J* = 5.8 Hz, 3H, CH_3_), 1.75 (s, 3H, CH_3_); ^13^C NMR (75 MHz, CDCl_3_): *δ* 166.5, 141.9, 141.2, 137.6, 129.6, 121.4, 110.8, 45.0, 20.3, 18.4; HRMS calcd. for C_10_H_15_NO ([M + H]^+^), 166.1226; found, 166.1225.

*N-*(2-methylallyl)hexanamide (**4j**). Yellow liquid; Yield 61%; ^1^H NMR (300 MHz, CDCl_3_): *δ* 5.72 (s, 1H, NH), 4.84 (dt, *J* = 2.6, 1.3 Hz, 2H, CH_2_), 3.82 (dd, *J* = 6.0, 1.4 Hz, 2H, CH_2_), 2.20–2.25 (m, 2H, CH_2_), 1.74–1.75 (m, 3H, CH_3_), 1.62–1.69 (m, 2H, CH_2_), 1.29–1.38 (m, 4H, CH_2_), 0.89–0.93 (m, 3H, CH_3_); ^13^C NMR (75 MHz, CDCl_3_): *δ* 173.1, 141.9, 110.4, 44.7, 36.4, 31.3, 25.4, 22.2, 20.1, 13.7; HRMS calcd. for C_10_H_19_NO ([M + H]^+^), 170.1539; found, 170.1538.

*N*-(2-methylallyl)heptanamide (**4k**). Colorless liquid; Yield 67%; ^1^H NMR (300 MHz, CDCl_3_): *δ* 5.87 (s, 1H, NH), 4.82 (p, *J* = 1.4 Hz, 2H, CH_2_), 3.81 (d, *J* = 6.0 Hz, 2H, CH_2_), 2.19–2.24 (m, 2H, CH_2_), 1.73 (s, 3H, CH_3_), 1.62–1.67 (m, 2H, CH_2_), 1.26–1.35 (m, 6H, CH_2_), 0.86–0.90 (m, 3H, CH_3_); ^13^C NMR (75 MHz, CDCl_3_): *δ* 173.2, 141.9, 110.3, 77.4, 77.0, 76.6, 44.6, 36.4, 31.4, 28.8, 25.7, 22.3, 20.1, 13.8; HRMS calcd. for C_11_H_21_NO ([M + H]^+^), 184.1696; found, 184.1694.

### 3.3. Phytotoxicity and Safening Effects of Compounds ***2a**–**k***, ***3a**–**k***, and ***4a**–**k*** on Rice Seedlings

Full rice seeds (*Oryza sativa*) of uniform sizes were germinated via the reported method [18]. Briefly, the seeds were sterilized by 5% sodium hypochlorite solution and then washed in deionized water. Then, sterile seeds were soaked in deionized water for 24 h and germinated for another 36 h in a plant growth cabinet (28 ± 1 °C) in the dark. 

The phytotoxicity and safener activities of target compounds **2a**–**k**, **3a**–**k**, and **4a**–**k** on rice seedlings were evaluated under greenhouse conditions according to the agar culture method we reported previously [54,55]. First, a 12 g agar strip was added to 3 L deionized water at 100 °C and constantly stirred over heat until the complete dissolution of the agar strip. Second, the mixture was diluted to 4 L with deionized water to obtain a 0.3% agar medium solution. Then, a standard working solution of metolachlor (**Mcl**, 960 g/L) was obtained by diluting 0.1 mL emulsifiable concentrate (960 g L^−1^) in 99.9 mL deionized water. A 600 mL aliquot of the agar medium was cooled to 45 °C and added to three 250 mL plastic boxes, then cooled further until solidification (each box contained 200 mL solution). An 88.75 μL volume of the **Mcl** working solution was mixed with 1.2 L 0.3% agar medium solution to obtain an agar medium with 0.25 μM **Mcl**. Three volumes of 200 mL 0.25 μM **Mcl** agar medium were added to three 250 mL plastic boxes at 45 °C and cooled until solidification. Using the same method, solidified agar media (without metolachlor) containing 8 mg/L of the target compounds (**2a**–**k**, **3a**–**k**, and **4a**–**k**) were prepared to test the phytotoxicity of target compounds on rice seedlings. 

Agar media containing 0.25 μM **Mcl**, **2a**–**k**, **3a**–**k**, **4a**–**k,** and the positive control dichlormid (**D**) was also prepared. **2a**–**k**, **3a**–**k**, **4a**–**k**, and **D** (each 2.4 g) were dissolved in separate 1 mL volumes of acetone, and then each was diluted to 12.5 mL using deionized water containing 0.5% Tween 80 to obtain 192 μg/μL standard solutions. A 40 µL volume of each standard solution was added to separate volumes of 1.2 L 0.25 μM **Mcl** agar medium to obtain an agar media containing 8 mg/L of each compound. Three 200 mL volumes of each agar medium were poured and set in three 250 mL plastic boxes, respectively.

Before shoot emergence, uniformly germinated rice seedlings were planted in different 0.3% agar mediums: 0.25 μM **Mcl**; 8 mg/L of compounds **2a**–**k**, **3a**–**k**, and **4a**–**k**; 8 mg/L compounds **2a**–**k**, **3a**–**k**, **4a**–**k**, and **D** with 0.25 μM **Mcl** and no additional compounds (non-treatment, control check). Fifty seeds were added to each plastic box and incubated for 14 h at 30 °C under a grow light (intensity 110–130 μE/m^2^/s), followed by a 10 h dark photoperiod at 25 °C. The various growth indexes related to herbicide safener activity (plant height, root length, and fresh weight) were measured after 7-day treatment. The phytotoxicity indexes of compounds **2a**–**k**, **3a**–**k**, and **4a**–**k** on rice seedlings and their herbicide safening indexes were further used for the analysis of structure-activity relationship (SAR) according to the following equations:

Plant height relative value = (plant height under each treatment)/(the average height of non-treatment) × 100%(1)

Root length relative value = (root length under each treatment)/(the average root length of non-treatment) × 100%(2)

Fresh weight relative value = (fresh weight under each treatment)/(the average fresh weight of non-treatment) × 100%(3)

Compounds deemed to have high herbicide safener activities based on plant height were screened further for herbicide safener activities in subsequent experiments at lower concentrations (0.25 mg/L, 0.5 mg/L, 1 mg/L, 2 mg/L, and 4 mg/L). All experiments on herbicide safener activity were carried out in three replications.

### 3.4. Enzyme Activity Assays

Rice seedling samples were collected after the 7-day treatments with 0.25 μM metolachlor, the combination of 8 mg L^−1^ compounds **2k**, **4k**, and dichlormid with 0.25 μM metolachlor, and control check (CK, no-treatment). 0.1 g of rice seedling tissues from the above treatments were first homogenized via a high-speed electric homogenizer, then filtered, and a 1 mL enzyme extraction solution was added. After centrifugation (12,000 r/min for 10 min), the supernatant was collected for use in enzyme activity assay.

Glutathione *S*-transferase (GST: EC 2.5.1.18) activity was examined by calculating the quantity of glutathione conjugate (composed of glutathione (GSH) and chlorodinitrobenzene (CDNB)) at 340 nm [56]. The activity was expressed as the enzyme unit nmol/min/g FW. Superoxide dismutase (SOD: EC 1.15.1.1) activity was measured through the method reported previously by calculating reduction inhibition of WST-1 (5-(2,4-disulfophenyl)-2-(4-iodophenyl)-3-(4-nitrophenyl)-2*H*-tetrazolium inner salt sodium salt (1:1)) at 450 nm [57], and the activity was articulated as the unit U/g FW. Catalase (CAT: EC 1.11.1.6) activity was evaluated at 510 nm by estimating the breakdown of H_2_O_2_, and the CAT activity was expressed as the unit nmol/min/g FW [58]. Peroxidase (POD: EC 1.15.1.1) activity was measured at 470 nm by calculating the oxidation of O-dianisidine in the presence of H_2_O_2_, and the enzyme activity was articulated as ΔOD_470_/min/g FW [59]. Polyphenol oxidase (PPO: EC 1.10.3.1) activity was measured at 420 nm and expressed as the unit ΔOD_420_/min/g FW, based on the oxidation of nicotinamide adenine dinucleotide phosphate (NADPH) [60].

### 3.5. Acute Toxicities of ***2k***, ***4k***, and Dichlormid to Zebrafish Embryos

The acute toxicities (96h) of **2k**, **4k**, and dichlormid to zebrafish embryos were evaluated via the method reported by Chen et al. [61]. Zebrafish embryos (2 h post-fertilization) were randomly transferred into 2 mL of test solutions with varying concentrations of **2k**, **4k**, and dichlormid in 24-well plates. Experiments were carried out to test the acute toxicity (median lethal concentration, LC_50_ value) of **2k**, **4k,** and dichlormid at 96 h. The concentrations of **2k** and **4k** were 5, 10, 20, 40, and 80 mg/L, while that of dichlormid were 2.5, 5, 10, 20, 40, and 80 mg/L, respectively. For each concentration, 60 embryos were used, and all experiments were done in three replications. Experimental plates were covered with transparent lids to prevent evaporation and transferred to an incubator maintained at 27 ± 1 °C with a 14:10 h light/dark photoperiod. The number of dead individuals and the state of embryonic development were examined daily. Reconstituted water containing 0.75 mmol/L Na^+^, 0.5 mmol/L Mg^2+^, 0.074 mmol/L K^+^, and 2 mmol/L Ca^2+^ was used to prepare all test solutions and a control solvent of 0.01% acetone (control check, *v*/*v*). The water solubility of **2k** and **4k** was evaluated by the reported method [62,63], and the solubility of **2k** and **4k** was 1.23 g/L and 3.13 g/L at 25 °C, respectively.

### 3.6. Statistical Analysis

All data were analyzed by one-way analysis of variance (ANOVA; general linear models on Origin 8.0 procedure). The Bonferroni’s test was used for mean separation if the ANOVA test indicated significance. A value of *p* < 0.05 was considered statistically significant.

## 4. Conclusions

In summary, 33 *N*-alkyl amide derivatives (**2a**–**k**, **3a**–**k**, and **4a**–**k**) were synthesized from amines and saturated and unsaturated fatty acids through acylation and condensation via the scaffold hopping approach. The chemical structures of all target compounds were well confirmed by ^1^H-NMR, ^13^C-NMR, and HRMS. A primary test by the agar method demonstrated that most of the target compounds could protect rice seedlings from injury caused by the pesticide metolachlor. Notably, compounds **2k** and **4k** displayed excellent herbicide safener activities on plant height and showed relatively similar activities to the commercialized compound dichlormid. Further, we showed that compounds **2k** and **4k** had higher GST, SOD, CAT, POD, and PPO enzyme activities in treated rice seedlings relative to the metolachlor treatment. Results from the current work revealed that compounds **2k** and **4k** had good safener activities toward rice and are, thus, promising herbicide safener candidates for further structural optimization to identify novel herbicide safeners for chloroacetanilide herbicides that are eco-friendly to the environment.

## Figures and Tables

**Table 1:** Phytotoxicity of 7-day-old rice seedlings treated with 8 mg∙L^−1^ compounds **2a**–**k**, **3a**–**k, 4a**–**k**, and dichlormid (**D**). The plant height, root length, and fresh weight were measured as phytotoxicity parameters.

Compound	Safening Effect (% of Non-Treated Control)		
	Plant Height	Root Length	Fresh Weight
**D**	93.1 ± 1.0	89.5 ± 2.0	93.4 ± 0.6
**2a**	90.3 ± 0.5	97.8 ± 0.5	91.3 ± 0.5
**2b**	95.9 ± 0.8	90.7 ± 0.6	95.1 ± 0.6
**2c**	88.3 ± 0.7	79.9 ± 0.4	89.2 ± 0.8
**2d**	95.8 ± 0.5	81.1 ± 0.4	88.7 ± 1.1
**2e**	95.6 ± 0.2	83.8 ± 0.2	90.8 ± 0.7
**2f**	96.9 ± 1.2	81.9 ± 0.6	92.9 ± 0.2
**2g**	97.5 ± 0.6	88.4 ± 0.2	91.2 ± 0.6
**2h**	95.2 ± 0.4	86.3 ± 1.0	84.0 ± 0.7
**2i**	99.4 ± 0.3	87.5 ± 2.4	96.5 ± 0.3
**2j**	97.0 ± 0.3	84.6 ± 0.5	84.4 ± 0.8
**2k**	97.6 ± 0.8	85.2 ± 0.8	93.6 ± 1.1
**3a**	98.3 ± 0.7	92.8 ± 0.3	97.4 ± 0.7
**3b**	91.6 ± 0.8	98.8 ± 0.2	90.4 ± 0.2
**3c**	98.9 ± 0.2	78.2 ± 0.9	96.5 ± 0.3
**3d**	83.2 ± 0.5	96.0 ± 0.5	93.1 ± 0.6
**3e**	92.8 ± 1.0	86.5 ± 0.8	87.4 ± 0.8
**3f**	89.3 ± 0.6	91.2 ± 0.7	86.4 ± 0.6
**3g**	98.9± 0.9	98.5 ± 1.0	96.7 ± 0.6
**3h**	94.7 ± 0.8	95.6 ± 0.4	96.7 ± 1.1
**3i**	81.7 ± 0.2	91.5 ± 0.9	84.2 ± 0.9
**3j**	97.2 ± 0.4	91.1 ± 0.6	96.6 ± 0.3
**3k**	91.7 ± 0.5	87.1 ± 0.4	88.5 ± 0.7
**4a**	90.3 ± 0.1	94.8 ± 0.7	95.3 ± 1.1
**4b**	90.6 ± 0.3	90.0 ± 0.8	91.2 ± 0.8
**4c**	97.4 ± 0.8	67.4 ± 0.9	87.4 ± 1.1
**4d**	82.4 ± 0.4	81.7 ± 0.9	87.4 ± 0.5
**4e**	98.1 ± 1.1	90.2 ± 1.0	96.6 ± 0.2
**4f**	90.5 ± 0.3	92.4 ± 1.1	95.0 ± 0.7
**4g**	96.8 ± 0.5	89.3 ± 0.8	87.7 ± 0.7
**4h**	95.9 ± 0.7	97.4 ± 0.7	93.5 ± 1.2
**4i**	91.2 ± 0.3	91.7 ± 0.2	89.0 ± 0.7
**4j**	98.5 ± 0.4	87.5 ± 0.3	87.8 ± 0.5
**4k**	96.9 ± 0.2	99.2 ± 0.7	94.1 ± 0.4

The values represent the means of three replicates ± SE of each mean.

**Table 2:** Herbicide safening effect of 7-day-old rice seedlings treated with metolachlor. The combined formulations were 8 mg∙L^−1^ compounds **2a**–**k**, **3a**–**k,** and **4a**–**k**/0.25 μM metolachlor (**C** + **Mcl**) and 8 mg∙L^−1^ dichlormid/0.25 μM metolachlor (**D** + **Mcl**). The plant height, root length, and fresh weight were measured as parameters.

Compound	Compared to the Blank Control (%)		
	Relative Plant Height	Relative Root Length	Relative Fresh Weight
**Mcl**	43.6 ± 0.4	56.1 ± 2.0	75.0 ± 2.2
**D + Mcl**	92.6 ± 0.9	83.2 ± 2.5	92.7 ± 1.3
**2a + Mcl**	53.8 ± 2.3	65.9 ± 2.0	85.6 ± 3.1
**2b + Mcl**	62.1 ± 2.3	59.8 ± 3.0	81.6 ± 1.0
**2c + Mcl**	56.3 ± 1.9	64.7 ± 1.5	79.6 ± 2.8
**2d + Mcl**	54.8 ± 2.8	67.1 ± 2.7	83.3 ± 3.3
**2e + Mcl**	52.7 ± 0.8	63.9 ± 2.1	82.7 ± 1.6
**2f + Mcl**	58.5 ± 2.9	74.6 ± 0.7	79.0 ± 0.2
**2g + Mcl**	62.2 ± 1.2	68.1 ± 1.7	83.1 ± 1.8
**2h + Mcl**	64.4 ± 2.2	73.4 ± 2.5	85.8 ± 2.3
**2i + Mcl**	54.8 ± 2.8	67.1 ± 2.7	83.3 ± 3.3
**2j + Mcl**	70.1 ± 1.7	71.0 ± 0.7	81.4 ± 0.7
**2k + Mcl**	84.3 ± 2.0	81.1 ± 1.1	81.3 ± 1.3
**3a + Mcl**	52.0 ± 0.9	64.4 ± 1.9	82.7 ± 2.1
**3b + Mcl**	50.0 ± 1.2	76.2 ± 0.6	85.3 ± 3.1
**3c + Mcl**	56.3 ± 1.9	64.7 ± 1.5	79.6 ± 2.8
**3d + Mcl**	50.1 ± 2.2	60.8 ± 4.4	85.2 ± 2.8
**3e + Mcl**	53.9 ± 0.9	65.8 ± 2.6	83.1 ± 2.3
**3f + Mcl**	47.2 ± 1.5	64.6 ± 3.0	79.8 ± 3.2
**3g + Mcl**	53.2 ± 0.7	71.0 ± 1.5	86.3 ± 2.4
**3h + Mcl**	43.2 ± 1.5	83.0 ± 3.9	87.9 ± 0.8
**3i + Mcl**	48.0 ± 0.9	60.2 ± 2.4	82.6 ± 2.6
**3j + Mcl**	50.5 ± 0.8	70.5 ± 1.1	84.3 ± 3.1
**3k + Mcl**	50.2 ± 1.7	59.4 ± 1.0	81.8 ± 2.9
**4a + Mcl**	45.0 ± 2.9	57.3 ± 3.1	79.5 ± 2.7
**4b + Mcl**	49.8 ± 1.2	66.5 ± 3.1	83.5 ± 2.3
**4c + Mcl**	44.3 ± 1.6	56.4 ± 1.0	82.2 ± 3.1
**4d + Mcl**	56.3 ± 2.3	63.6 ± 2.2	81.1 ± 2.6
**4e + Mcl**	42.8 ± 0.3	56.8 ± 1.3	78.9 ± 1.4
**4f + Mcl**	53.7 ± 1.6	67.4 ± 1.3	78.3 ± 1.7
**4g + Mcl**	49.8 ± 0.9	60.4 ± 1.2	77.5 ± 3.3
**4h + Mcl**	64.4 ± 1.7	64.8 ± 4.0	84.7 ± 3.5
**4i + Mcl**	49.2 ± 1.1	63.1 ± 1.2	84.8 ± 2.9
**4j + Mcl**	63.5 ± 0.4	60.5 ± 0.4	76.8 ± 1.9
**4k + Mcl**	81.0 ± 0.4	82.2 ± 1.0	84.9 ± 0.6

The values present the means of three replicates ± SE of each mean.

**Table 3:** Acute toxicities of compounds **2k**, **4k**, and dichlormid to zebrafish embryos at 96 h after treatment.

Compound	LC_50_ (mg/L)	Regression Equation	*R* ^2^
**2k**	38.29	y = 1.7630 + 4.2719x	0.9451
**4k**	39.28	y = 1.7557 + 4.2376x	0.9456
dichlormid	7.86	y = 1.9248 + 3.4345x	0.8849

## Supplementary Materials

^1^H NMR and ^13^C NMR spectrums of target compounds **2a**–**k**, **3a**–**k**, and **4a**–**k**.
