# Supplementary material for: New Lead Discovery of Herbicide Safener for Metolachlor Based on a Scaffold-Hopping Strategy
Source: Molecules. 2020 Oct 28;25(21):4986. doi: 10.3390/molecules25214986 (PMC7663620; doi:10.3390/molecules25214986)

Supplementary Materials

New Lead Discovery of Herbicide Safener for metolachlor Based on a Scaffold-hopping Strategy

Xile Deng, Wenna Zheng, Qingcai Zhan, Yanan Deng, Yong Zhou, Lianyang Bai *

Biotechnology Research Institute, Hunan Academy of Agricultural Sciences, Changsha 410125, China; dengxile@hunaas.cn (X.D.); zhengwenna1994@163.com (W.Z.); zhanqc@hotmail.com (Q.Z.); dengyanan007@hunaas.cn (Y.D.); qimiaobuchugan@126.com (Y.Z.); lybai@hunaas.cn (L.B.)

* Correspondence: lybai@hunaas.cn (L.B.); Tel.: +86-0731-8469-2799 (L.B.)

N-isobutylacetamide (**2a**)

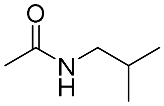

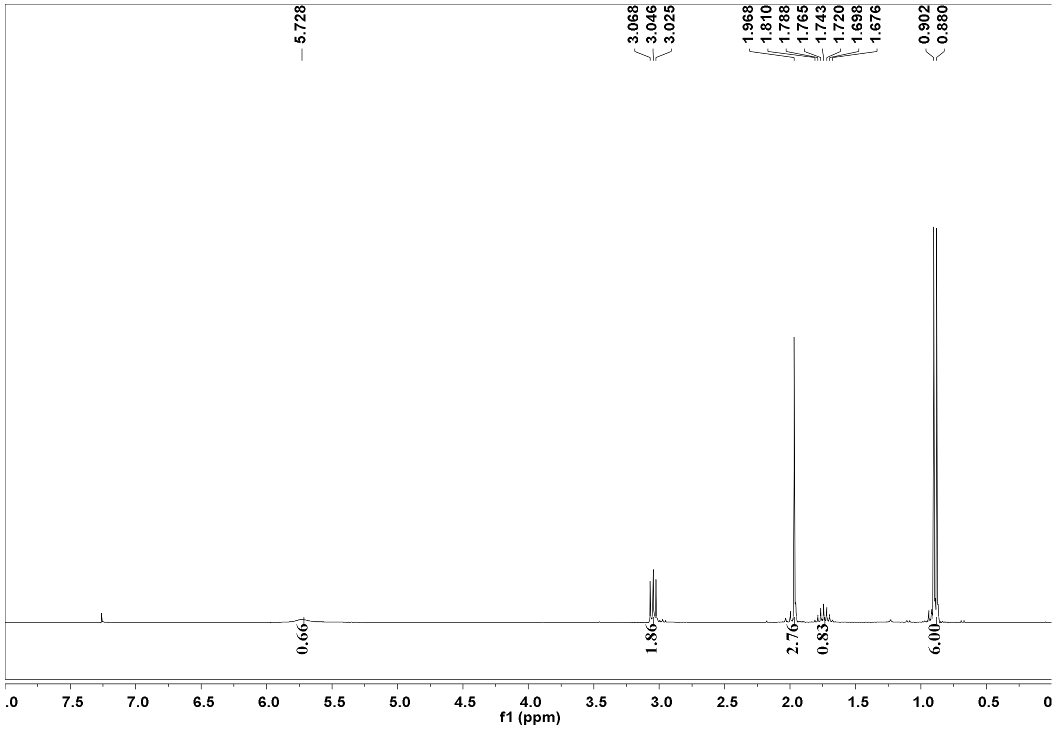

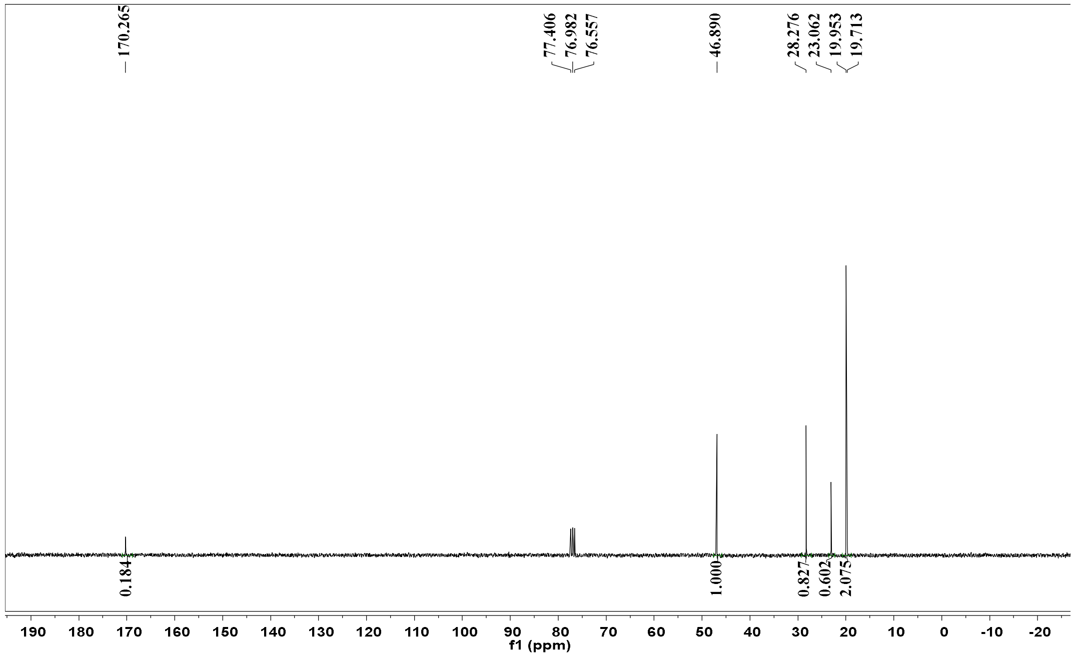

N-isobutylacrylamide (**2b**)

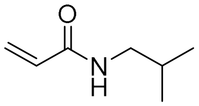

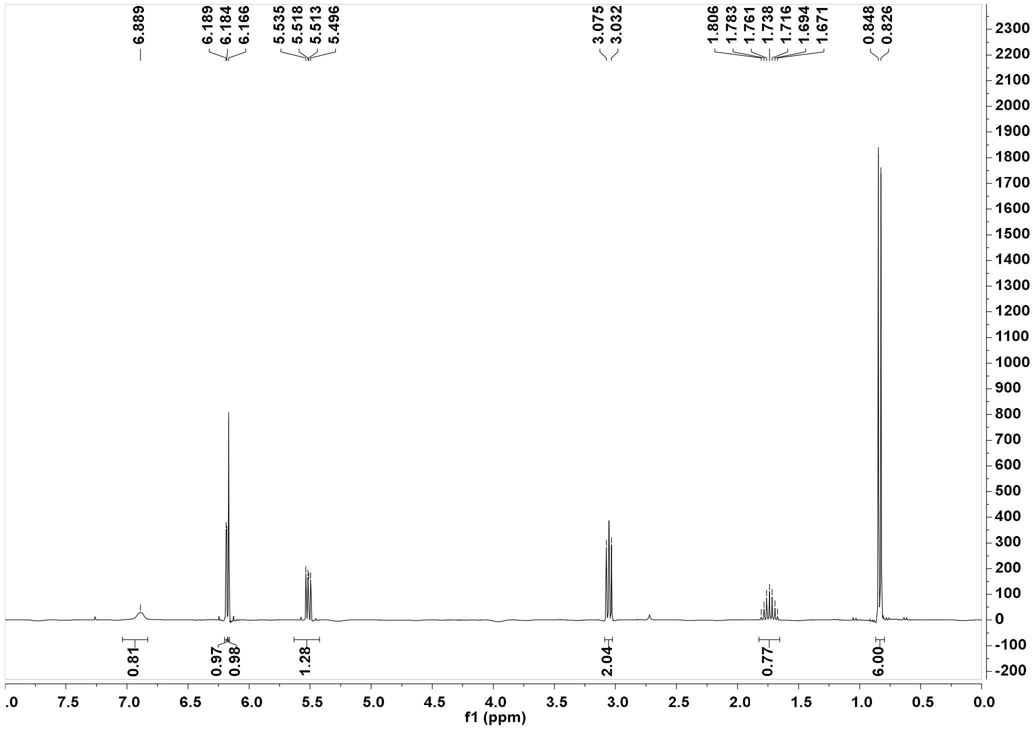

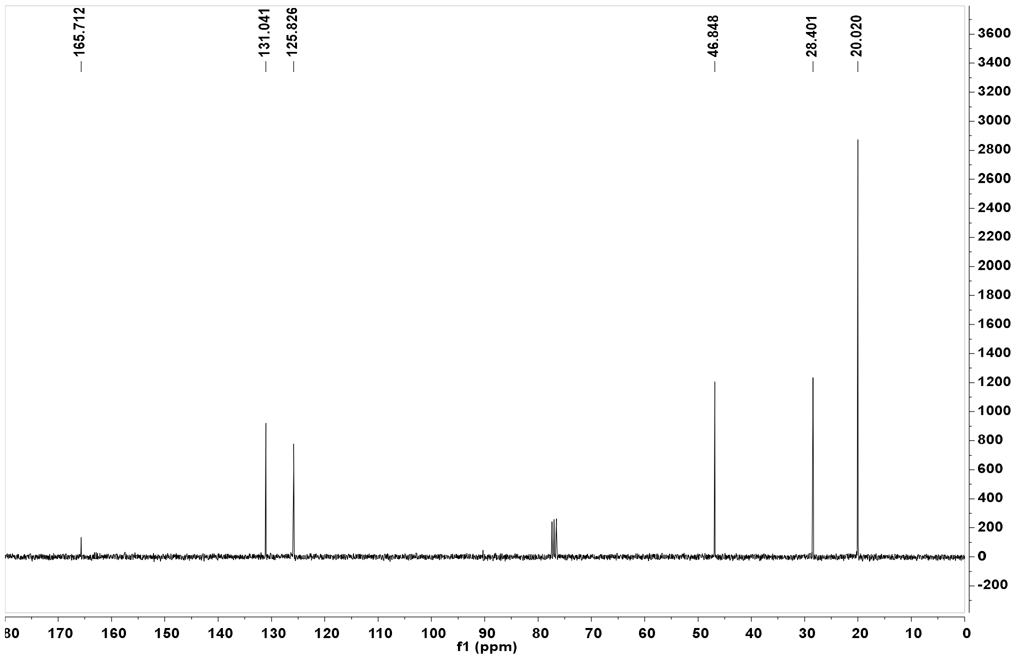

N-isobutylpropionamide (**2c**)

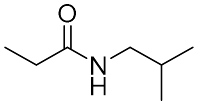

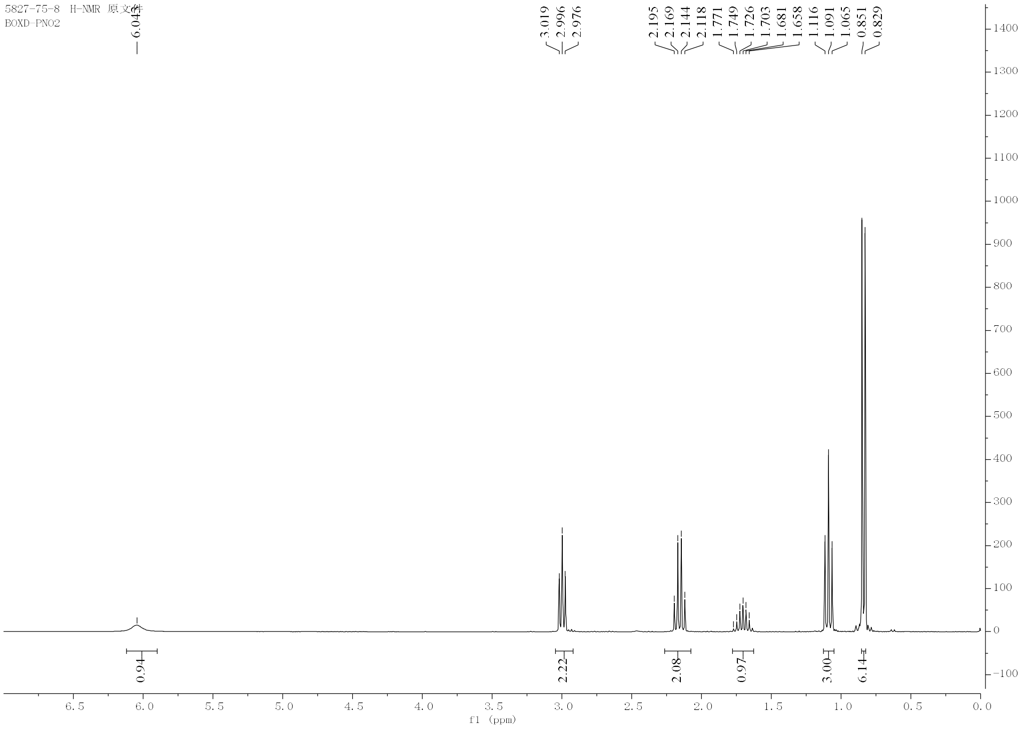

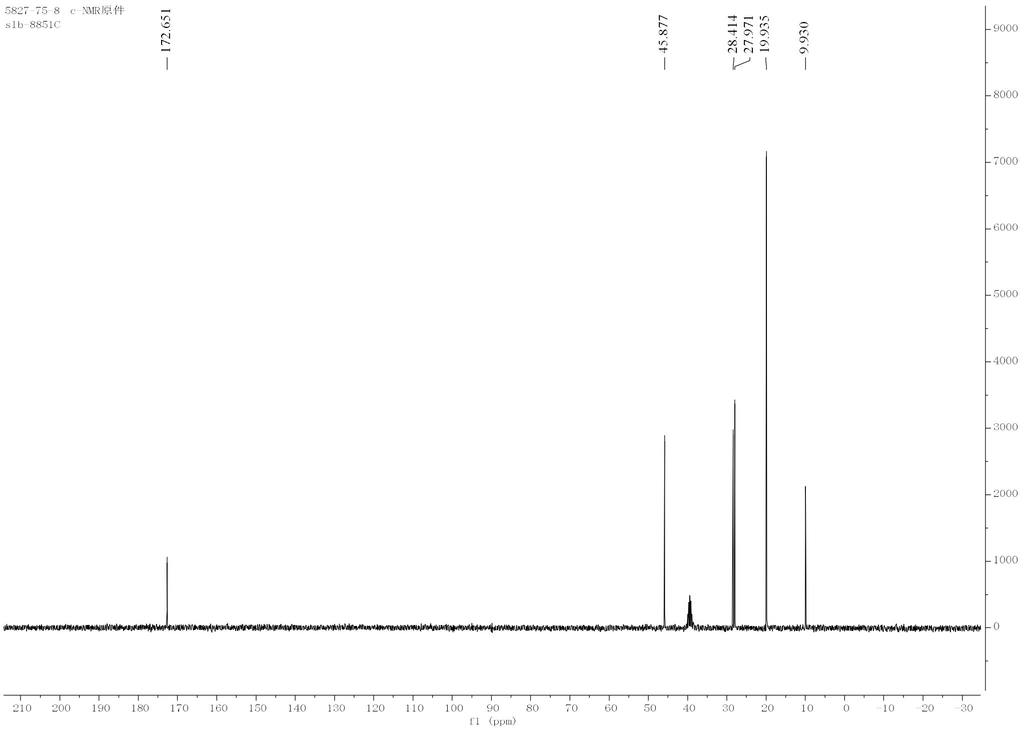

(*E*)-*N*-isobutylbut-2-enamide (**2d**)

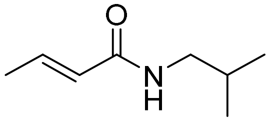

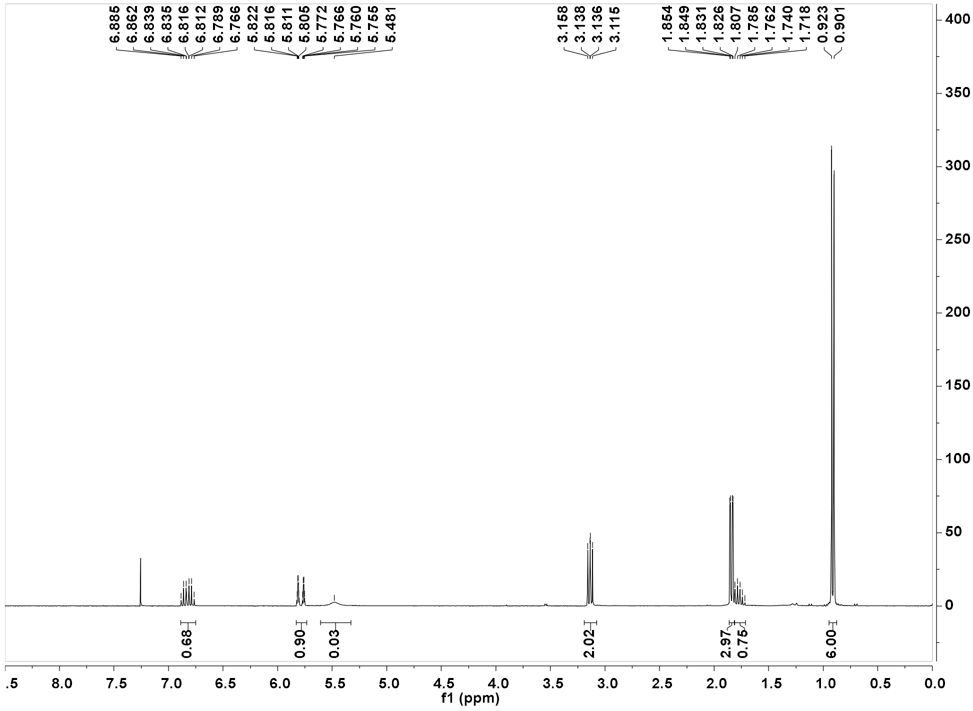

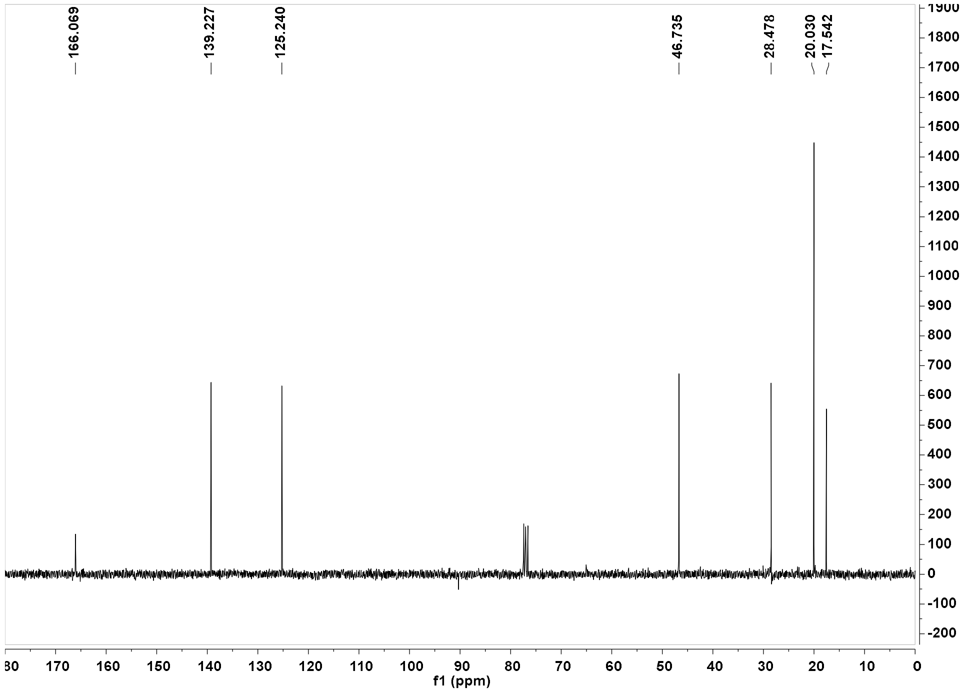

N-isobutylbutyramide (**2e**)

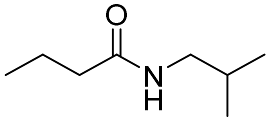

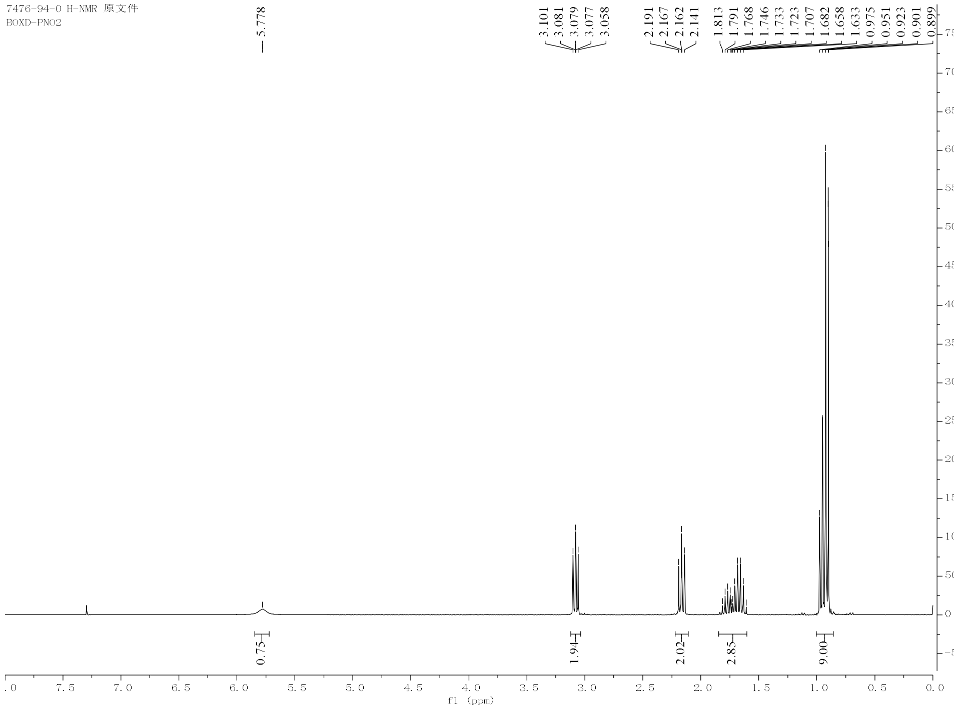

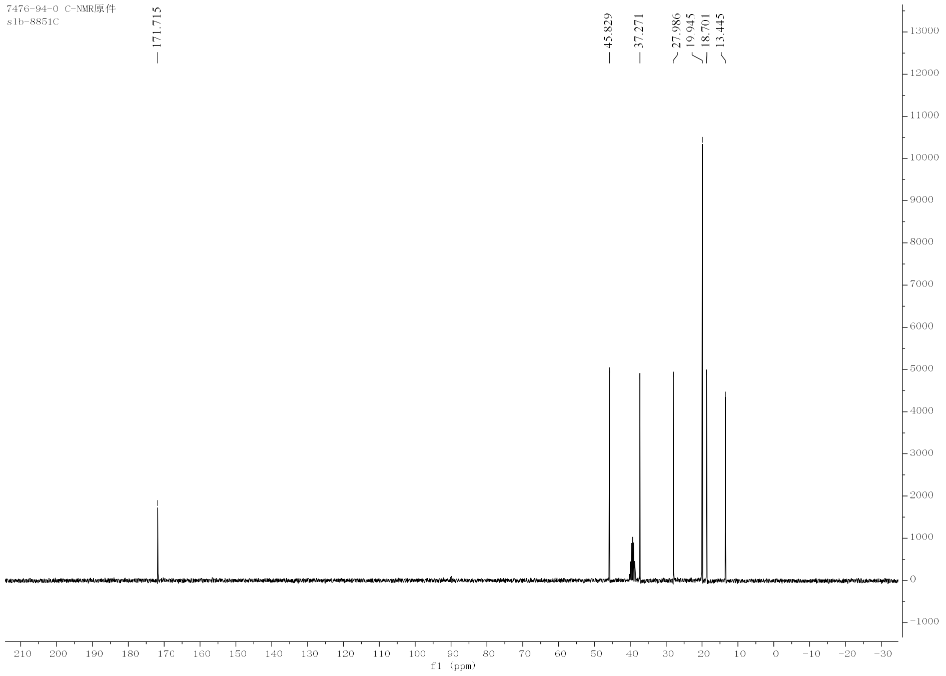

(*E*)-*N*-isobutylpent-2-enamide (**2f**)

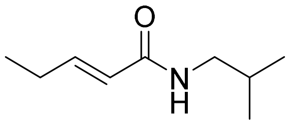
O
N
H

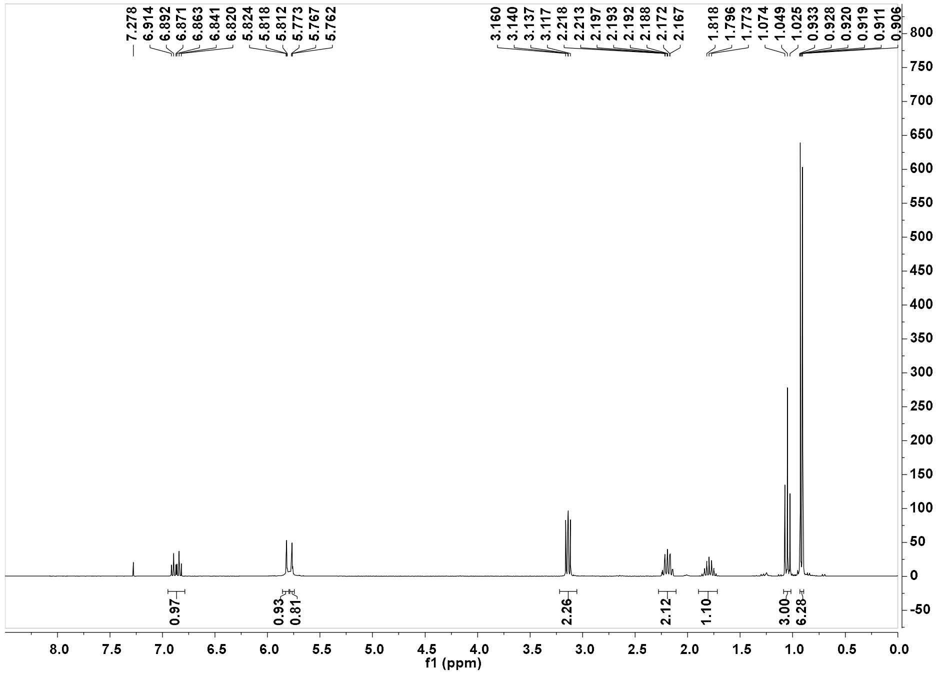
7.278
6.914
6.892
6.871
6.863
6.841
6.820
5.824
5.818
5.812
5.773
5.767
5.762
3.160
3.140
3.137
3.117
2.218
2.213
2.197
2.193
2.192
2.188
2.172
2.167
1.818
1.796
1.773
1.074
1.049
1.025
0.933
0.928
0.920
0.919
0.911
0.906
0.97
0.93
0.81
2.26
2.12
1.10
3.00
6.28
f1 (ppm)

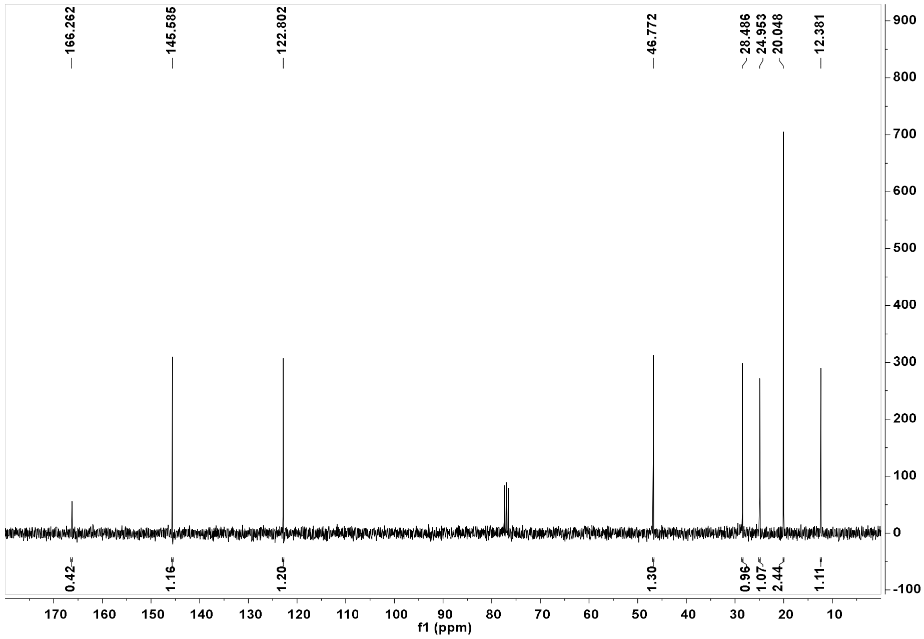
166.262
145.585
122.802
46.772
28.486
24.953
20.048
12.381
0.42
1.16
1.20
1.30
0.96
1.07
2.44
1.11
f1 (ppm)

N-isobutylpentanamide (**2g**)

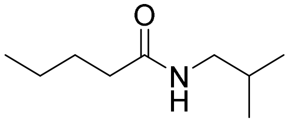

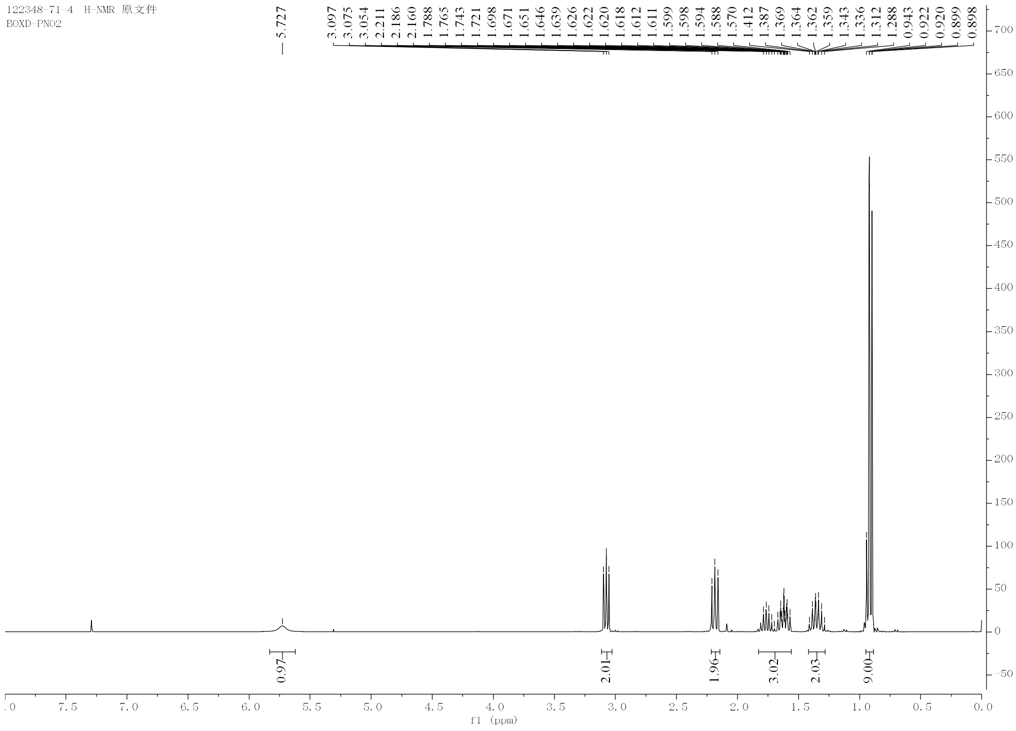

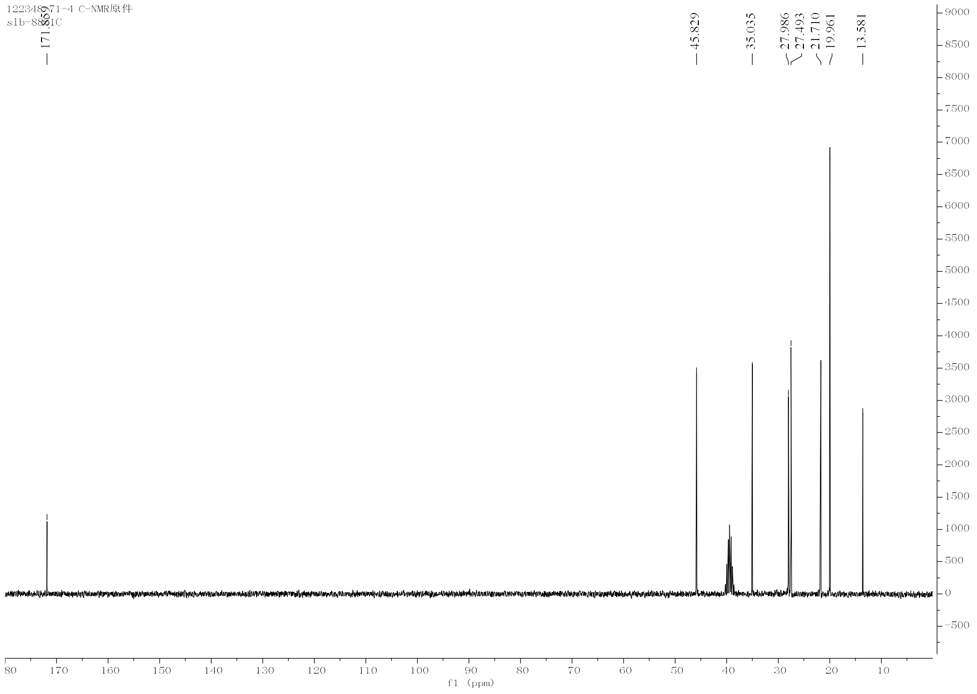

(*E*)-*N*-isobutylhex-2-enamide (**2h**)

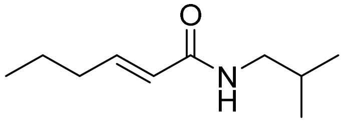

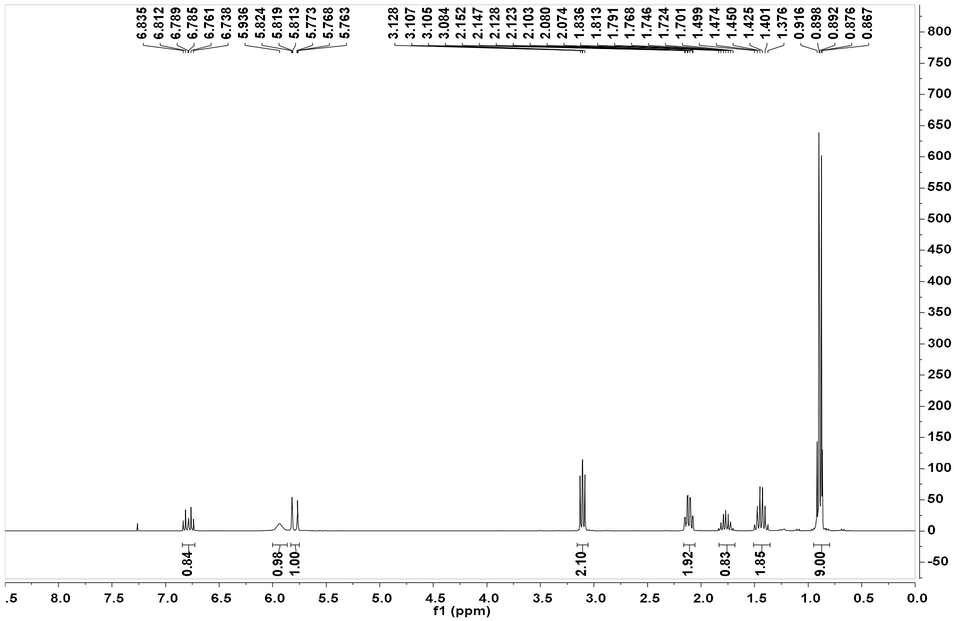

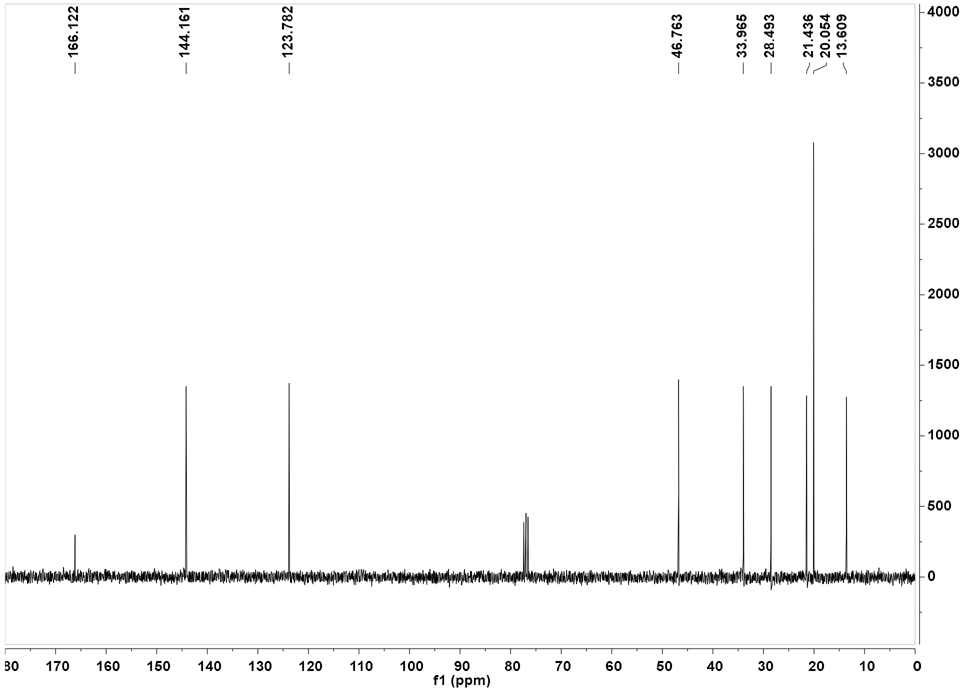

(2*E*, 4*E*)-*N*-isobutylhexa-2,4-dienamide (**2i**)

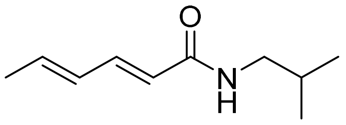

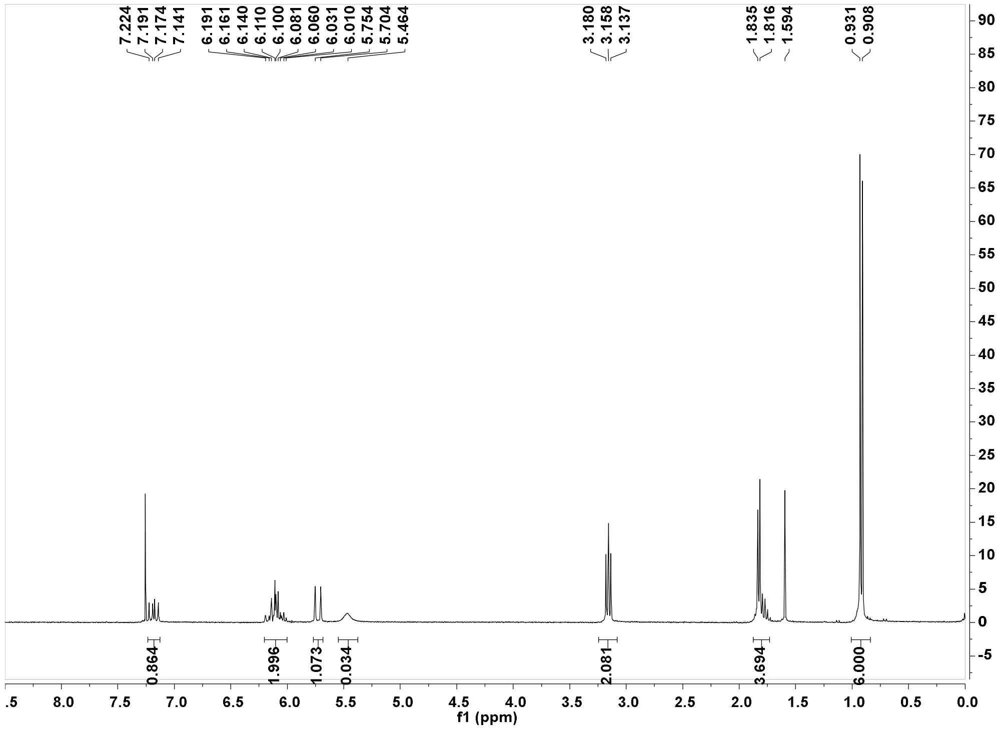
7.224
7.191
7.174
7.141
6.191
6.161
6.140
6.110
6.100
6.081
6.060
6.031
6.010
5.754
5.704
5.464
3.180
3.158
3.137
1.835
1.816
1.594
0.931
0.908
0.864
1.996
1.073
0.034
2.081
3.694
6.000
f1 (ppm)

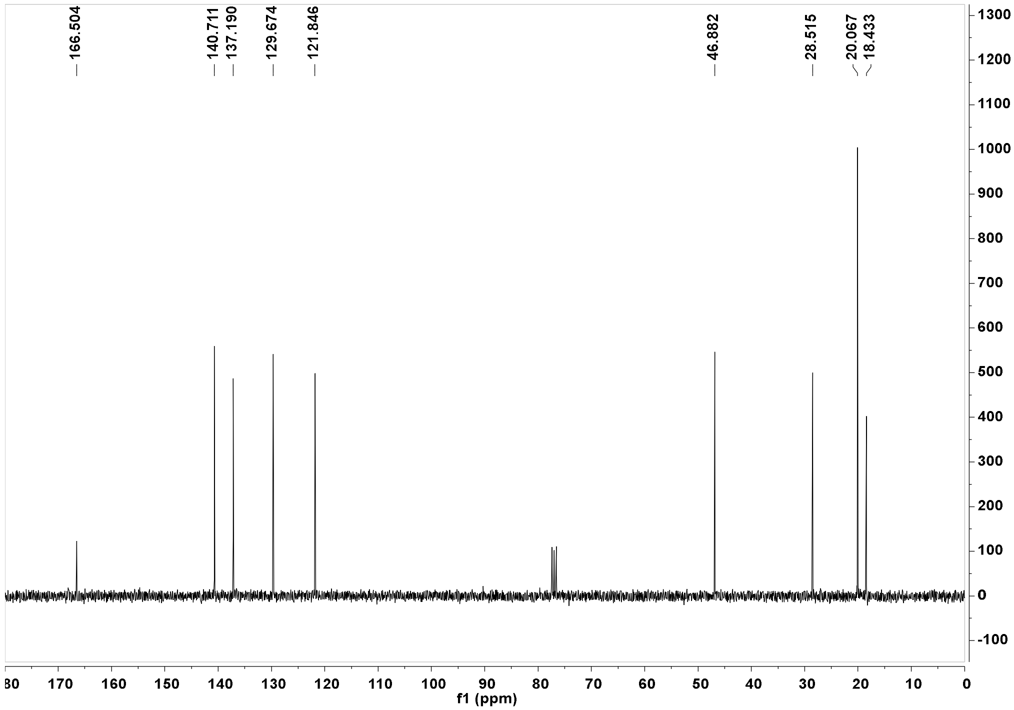
166.504
140.711
137.190
129.674
121.846
46.882
28.515
20.067
18.433
f1 (ppm)

N-isobutylhexanamide (**2j**)

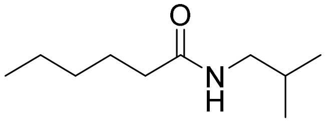

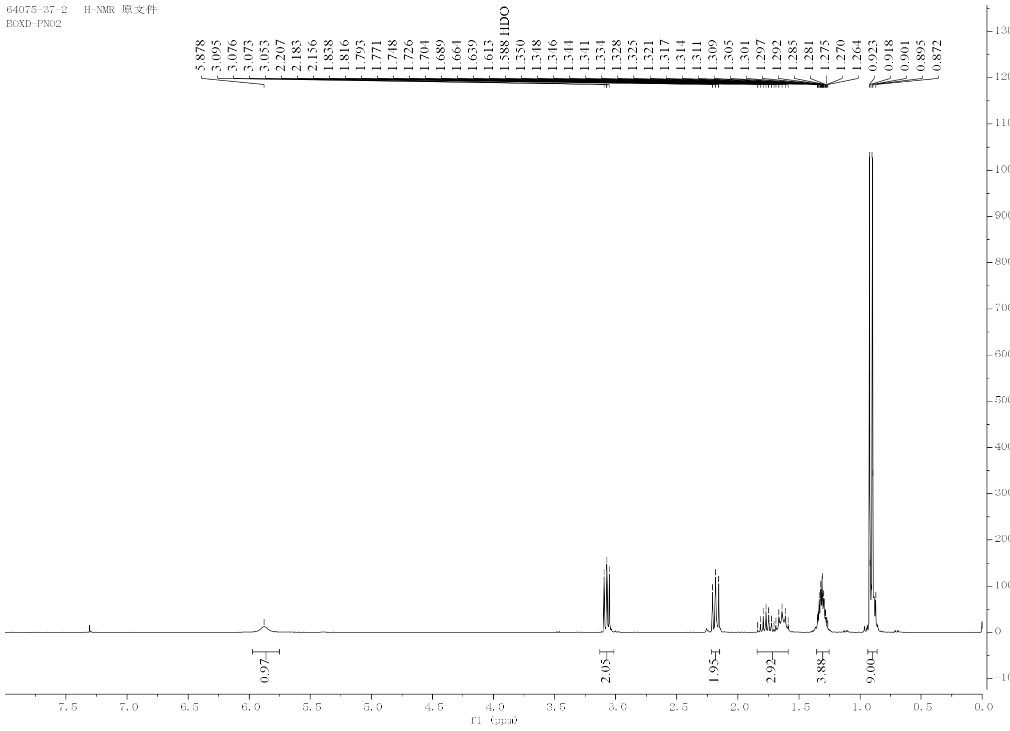

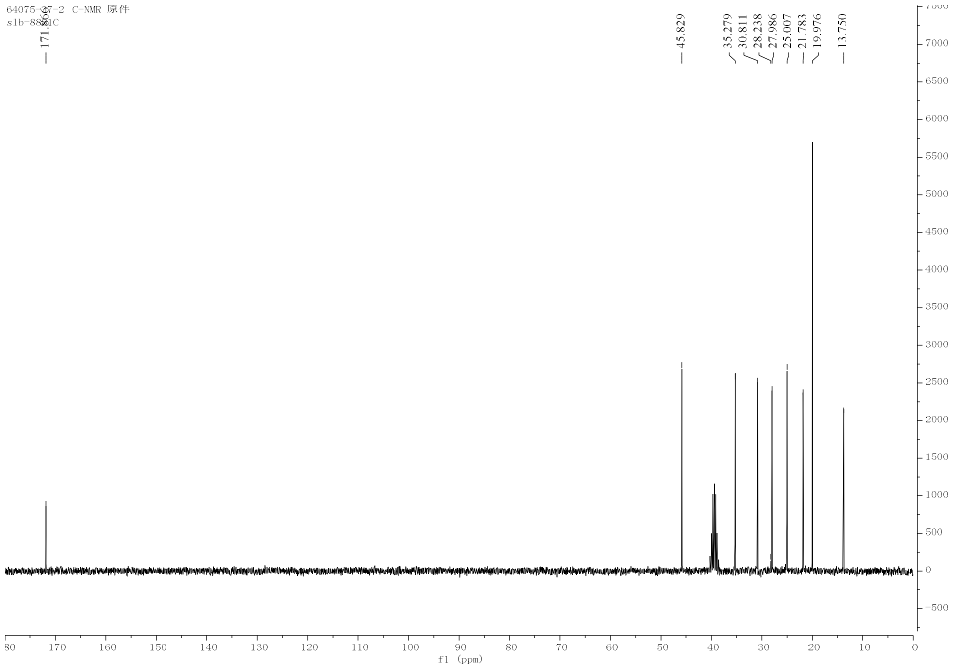

N-isobutylheptanamide (**2k**)

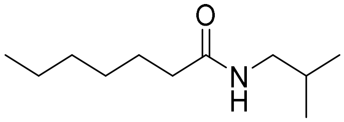

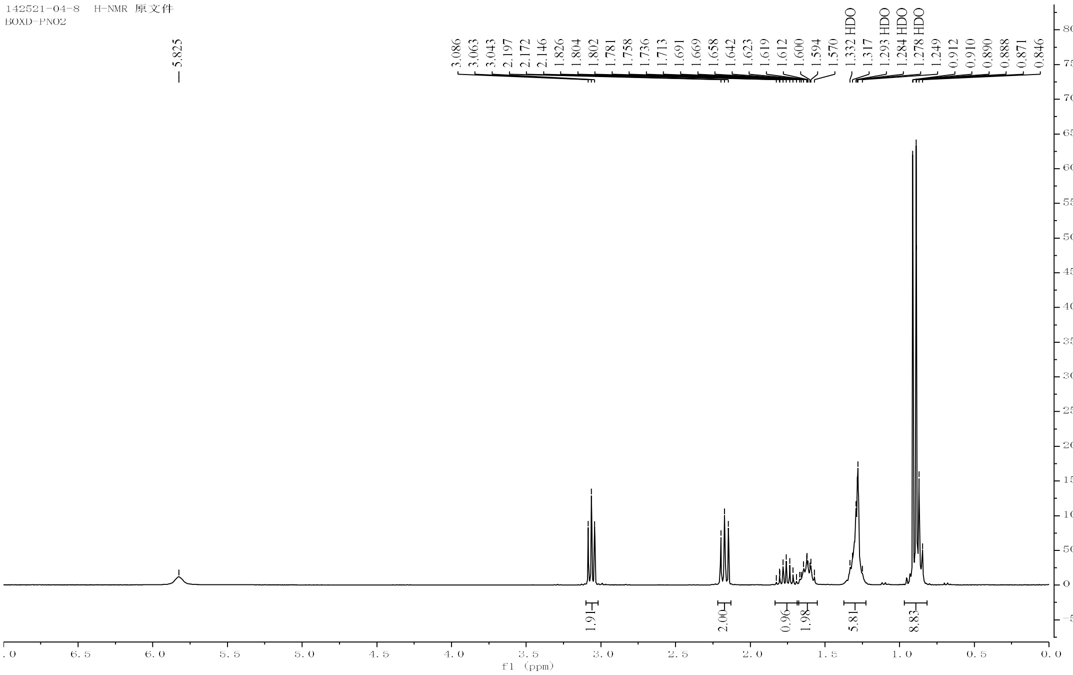

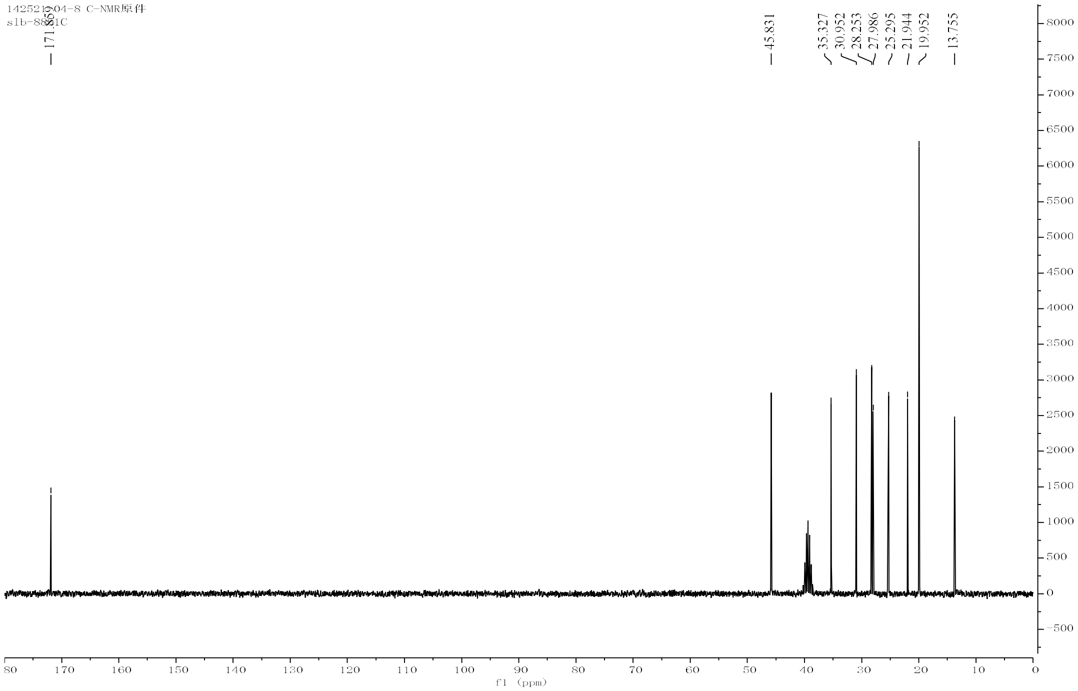

N-(2-hydroxy-2-methylpropyl)acetamide (**3a**)

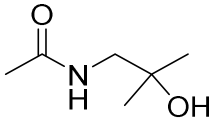

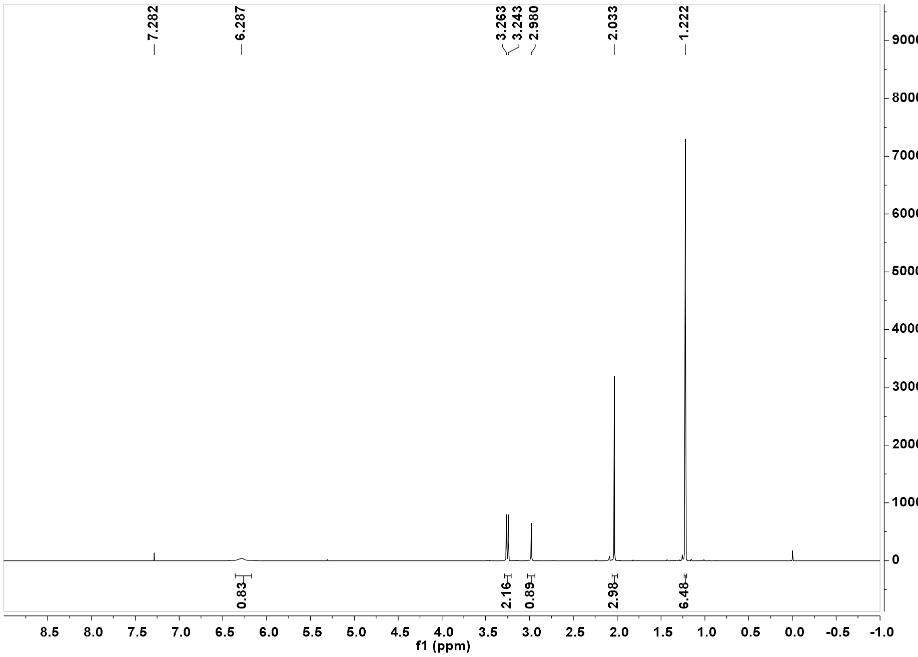

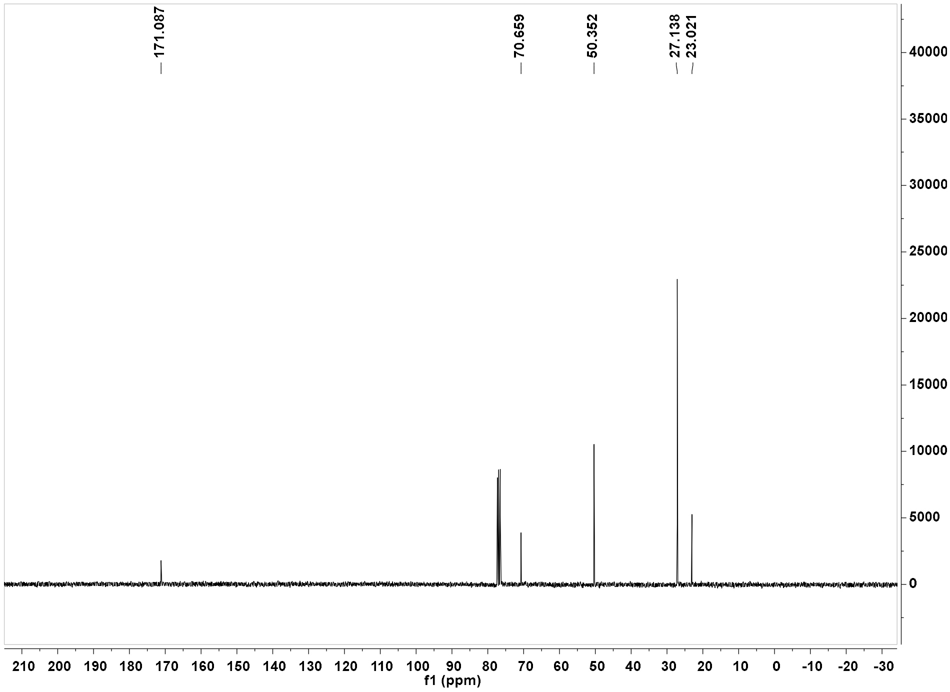

N-(2-hydroxy-2-methylpropyl)acrylamide (**3b**)

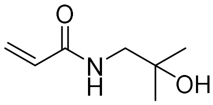

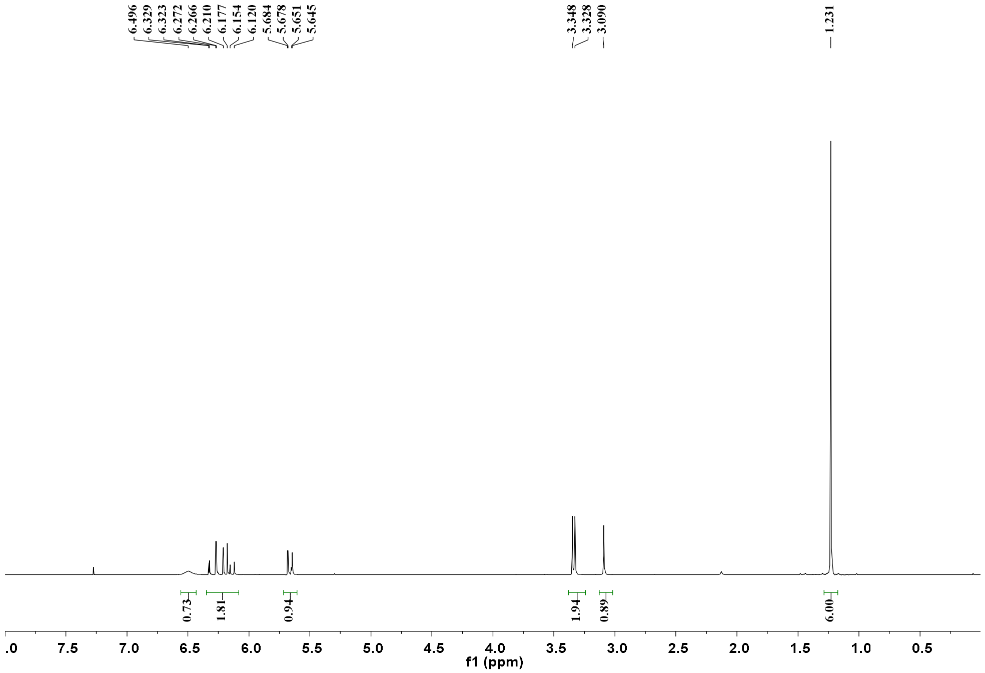

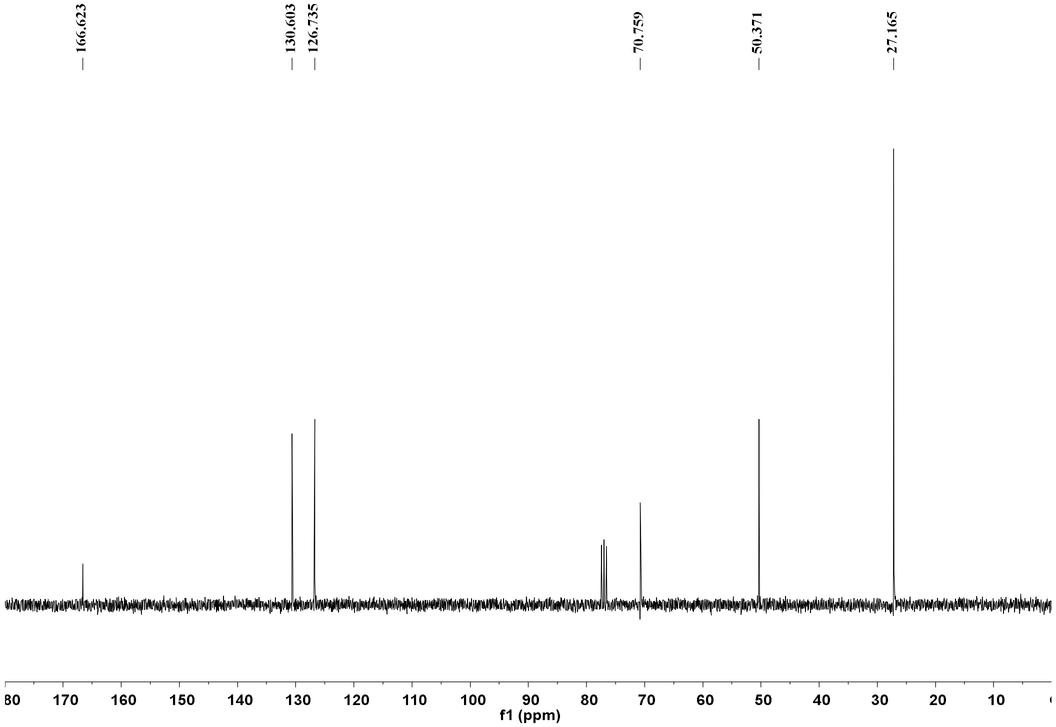

N-(2-hydroxy-2-methylpropyl)propionamide (**3c**)

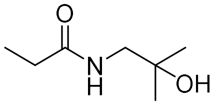
O
N
H
OH

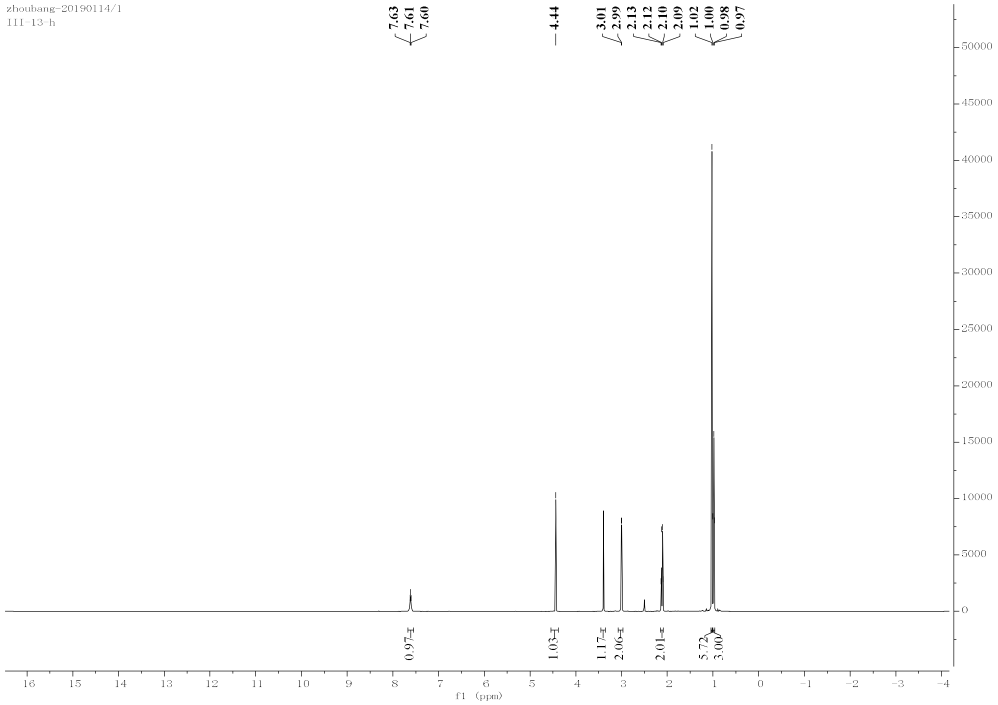
zhoubang-20190114/1
III-13-h
7.63
7.61
7.60
4.44
3.01
2.99
2.13
2.12
2.10
2.09
1.02
1.00
0.98
0.97
0.97
1.03
1.17
2.06
2.01
5.72
3.00
f1 (ppm)

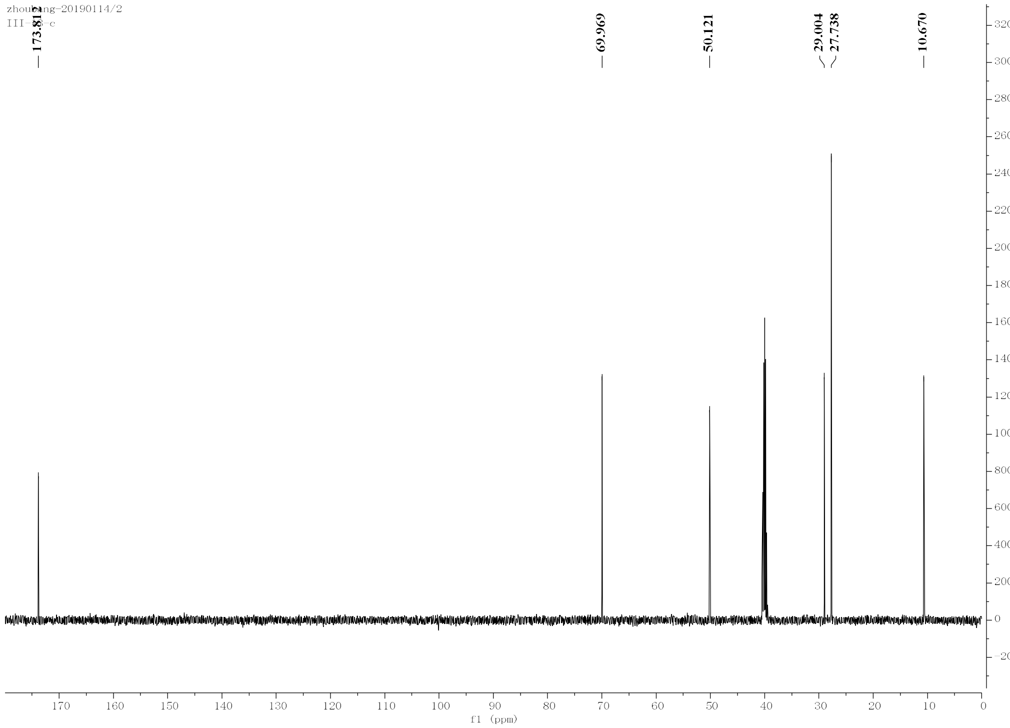
zhoubang-20190114/2
69.969
50.121
29.004
27.738
10.670
f1 (ppm)

(*E*)-*N*-(2-hydroxy-2-methylpropyl)but-2-enamide (**3d**)

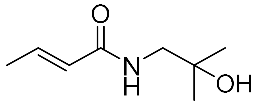

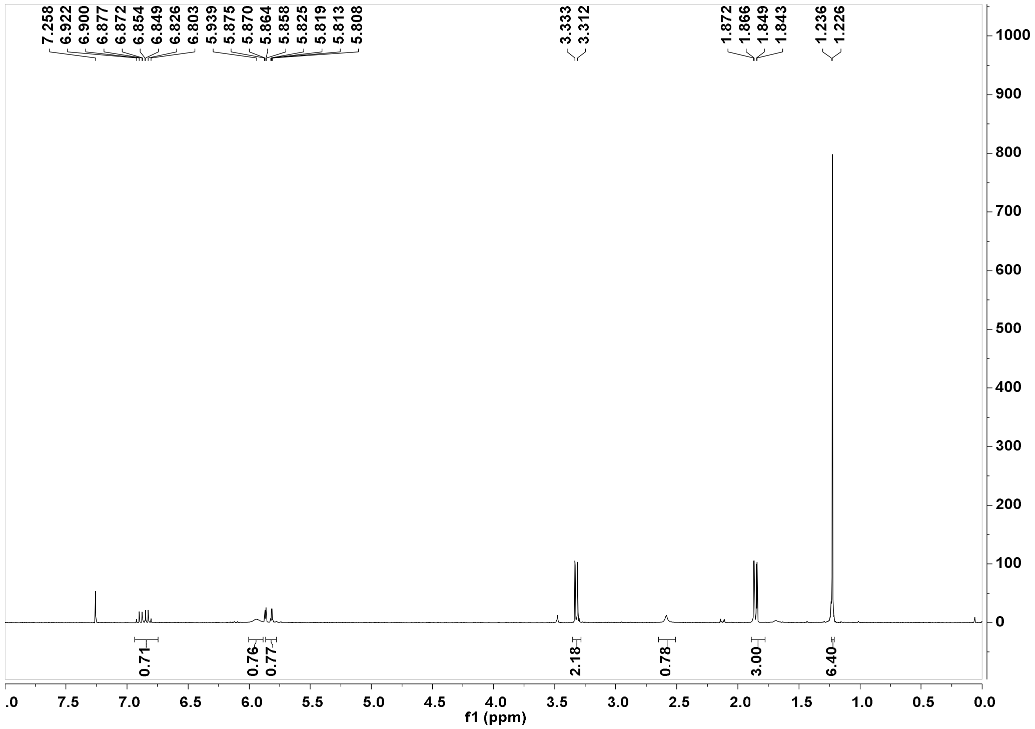

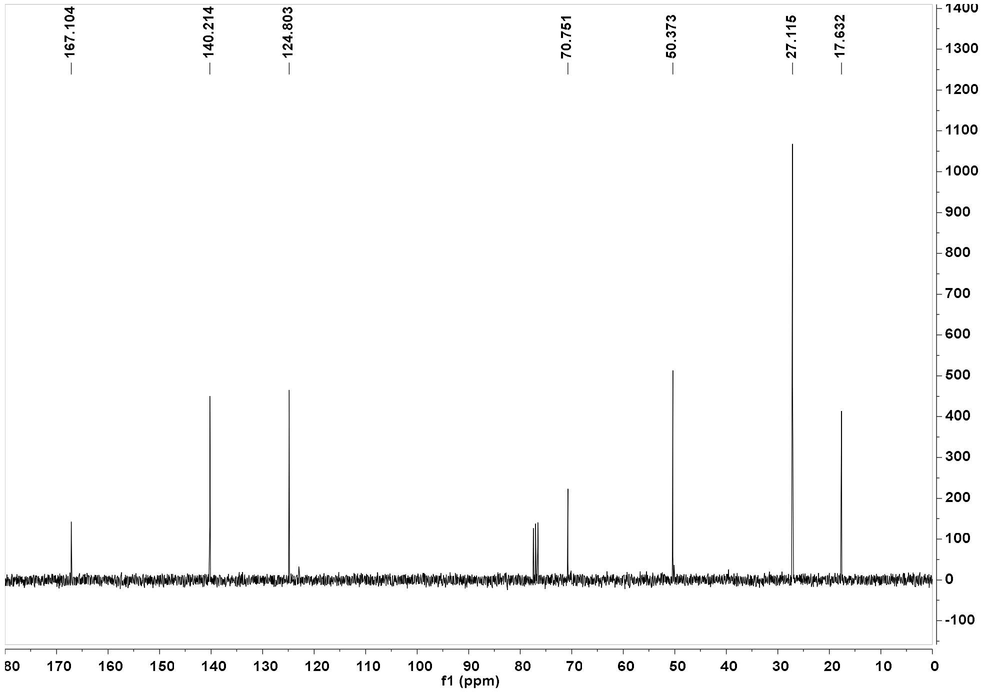

N-(2-hydroxy-2-methylpropyl)butyramide (**3e**)

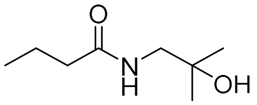

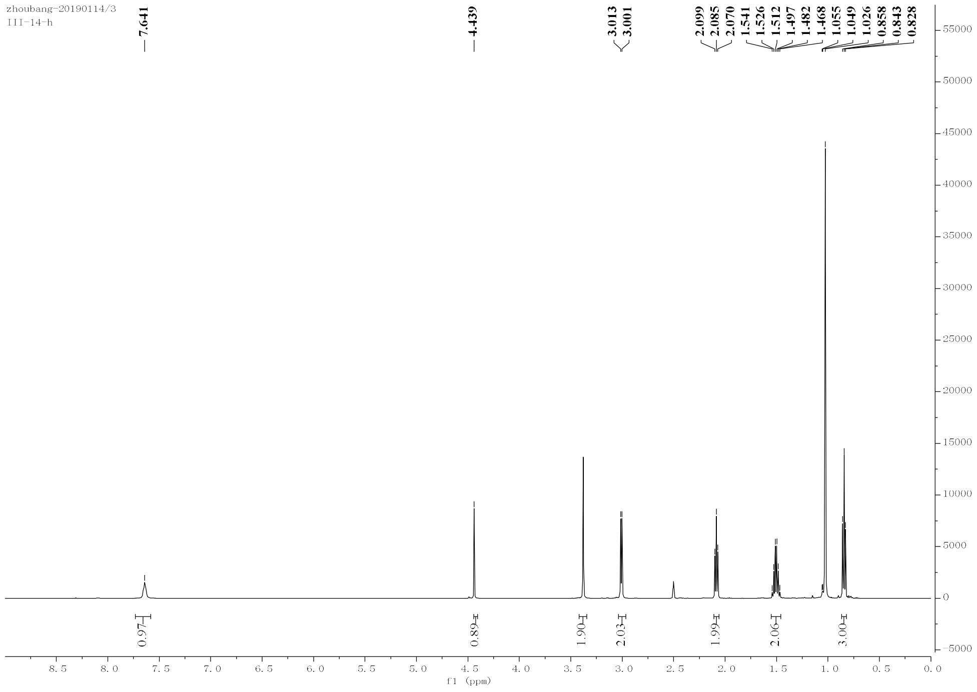

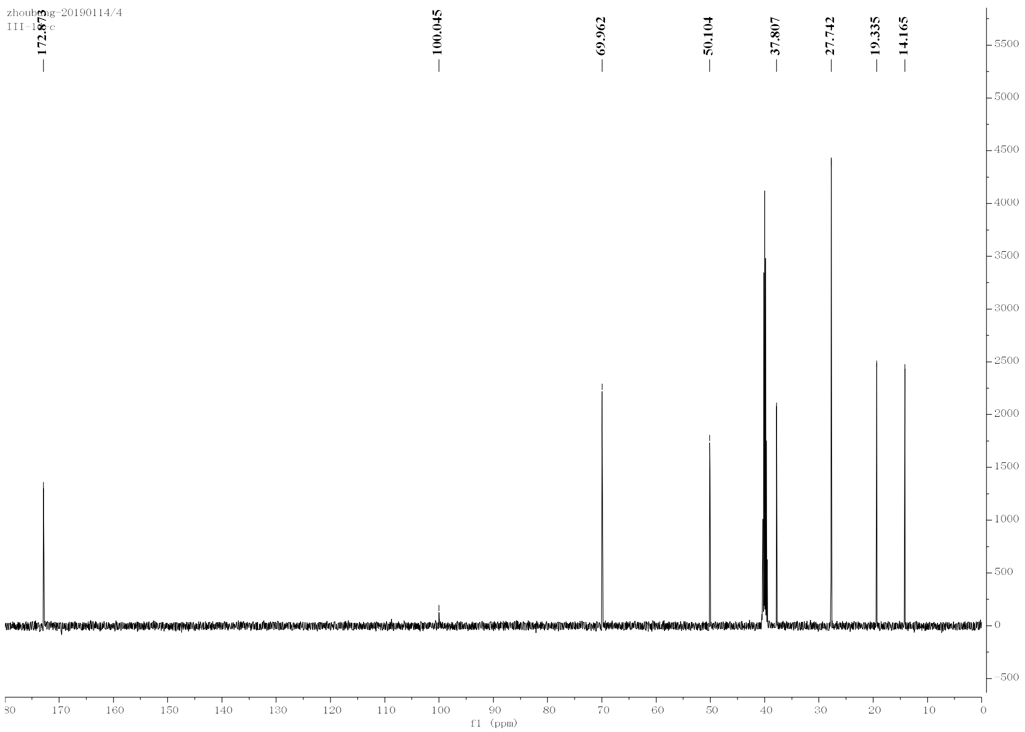

(*E*)-*N*-(2-hydroxy-2-methylpropyl)pent-2-enamide (**3f**)

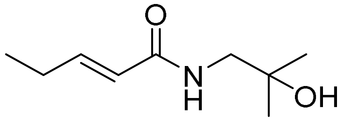

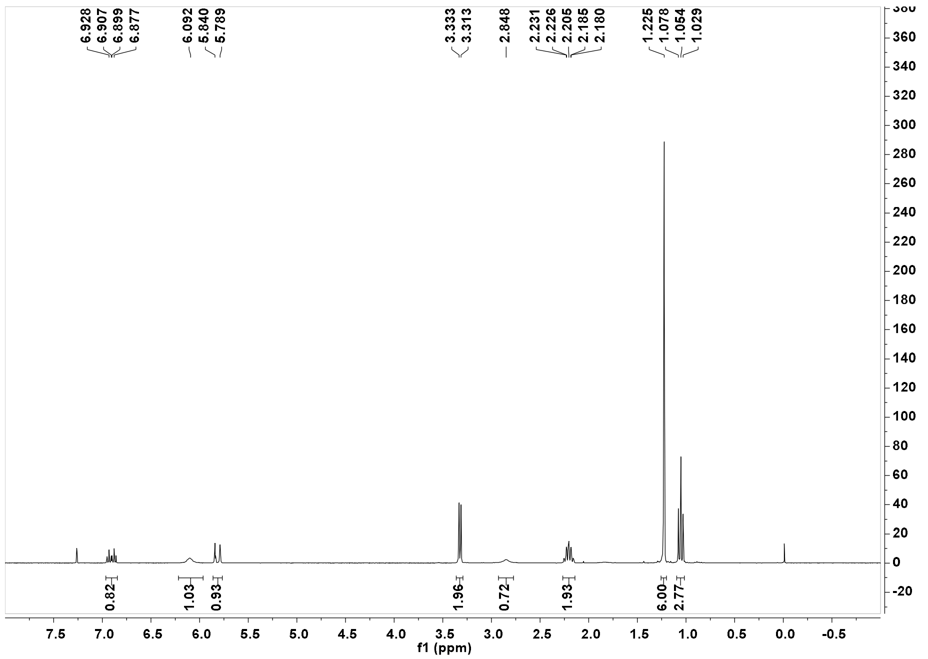

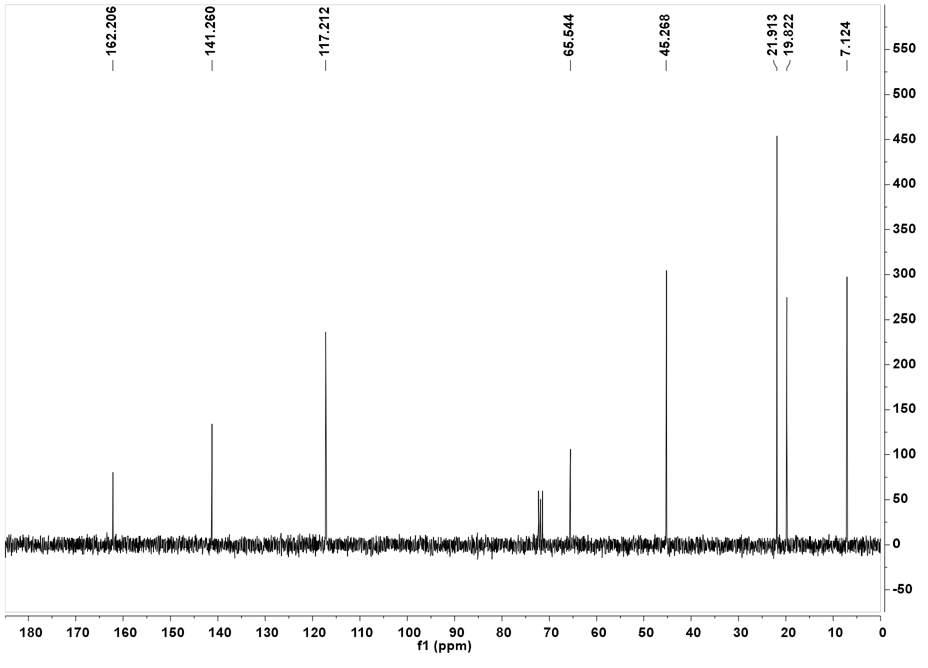

N-(2-hydroxy-2-methylpropyl)pentanamide (**3g**)

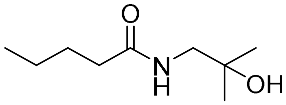

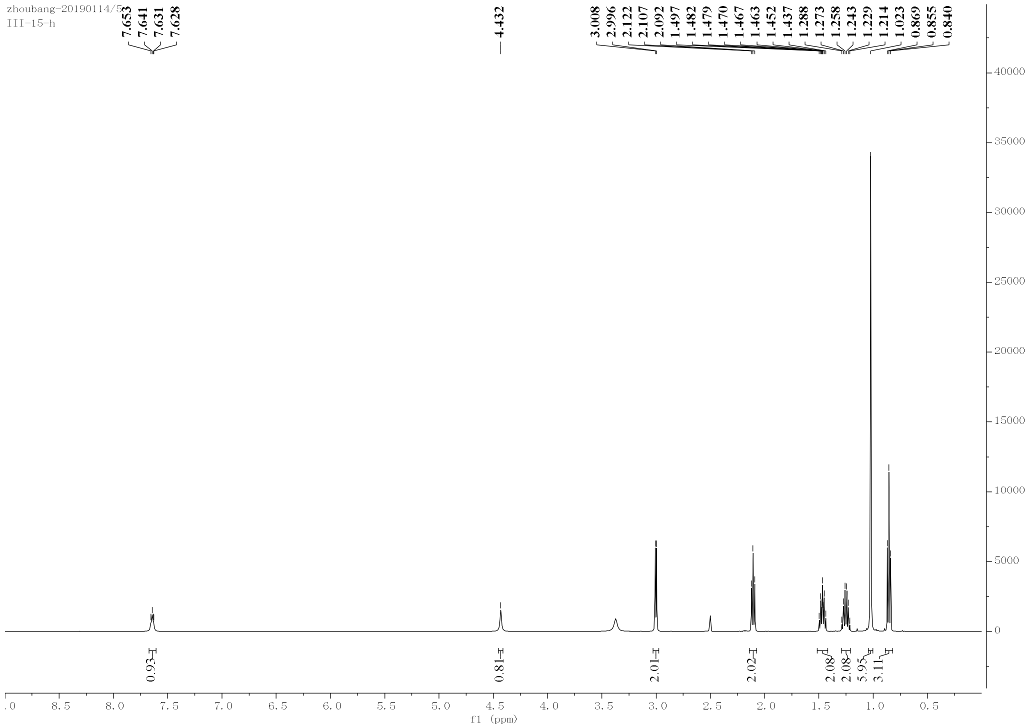

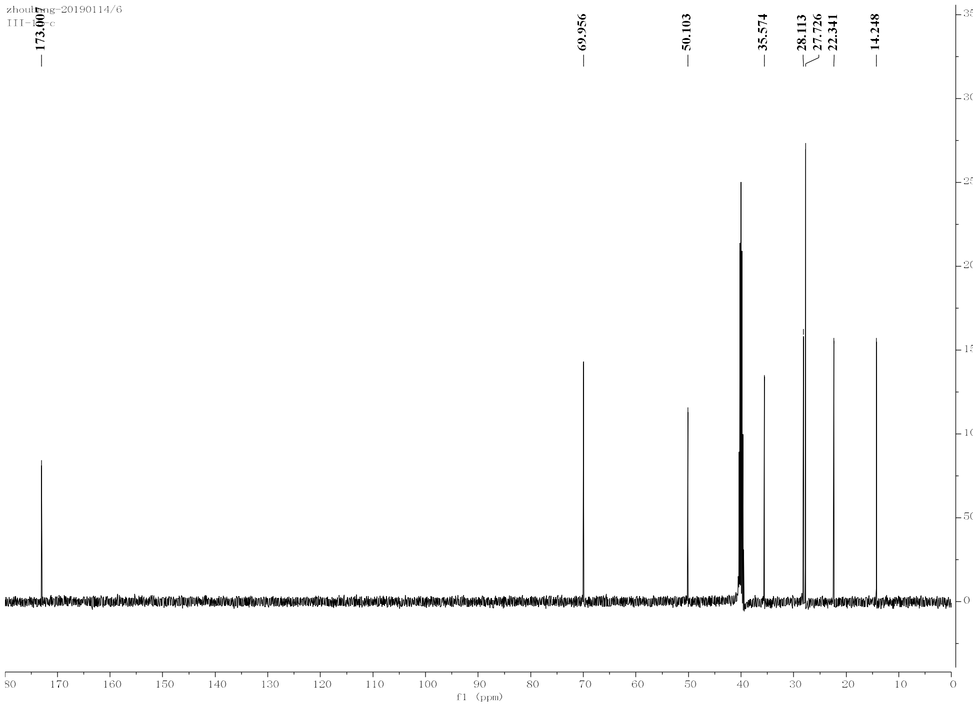

(***E***)-*N*-(2-hydroxy-2-methylpropyl)hex-2-enamide (**3h**)

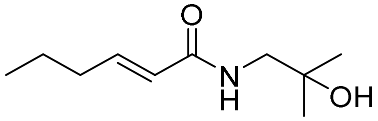

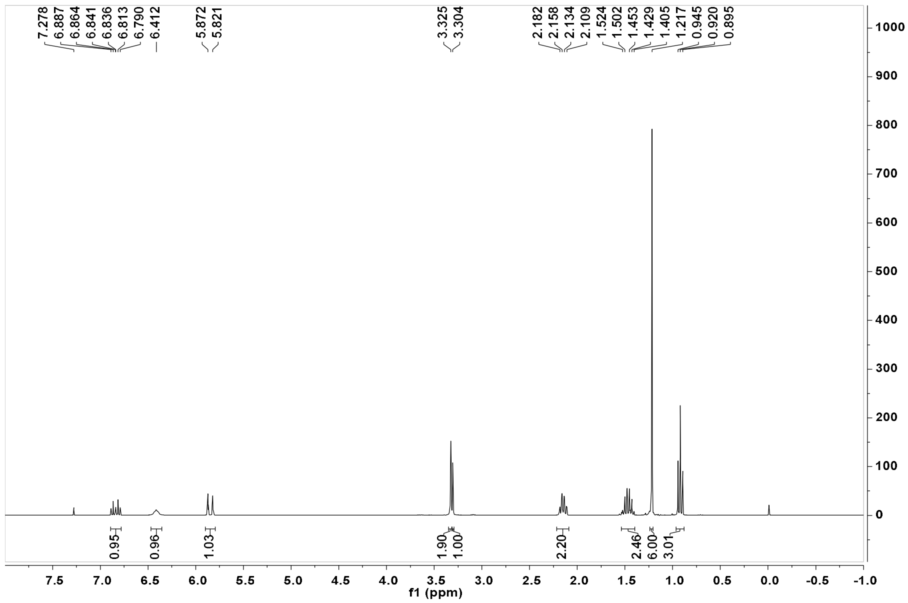

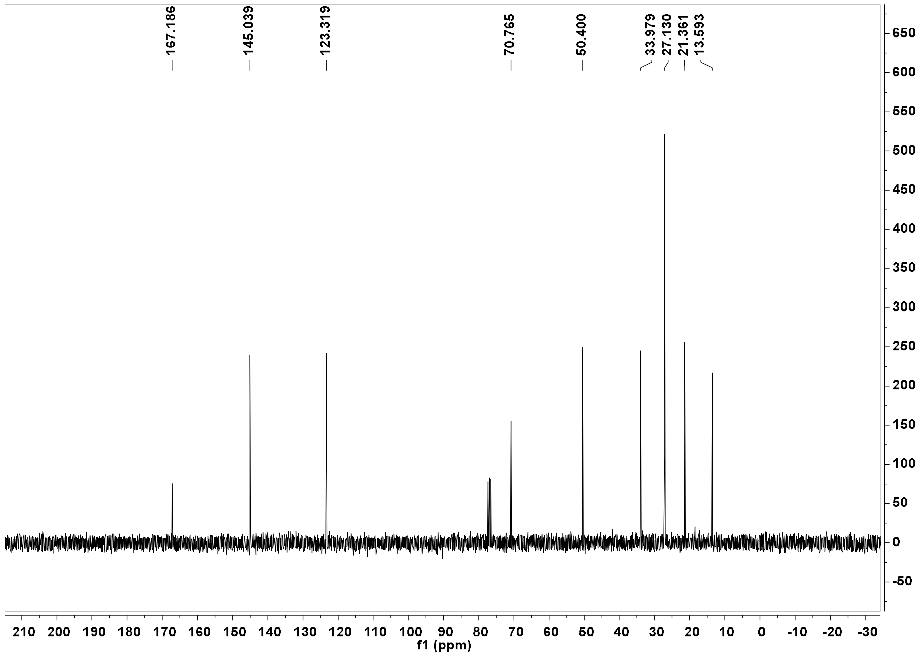

(2*E*,4*E*)-*N*-(2-hydroxy-2-methylpropyl)hexa-2,4-dienamide (**3i**)

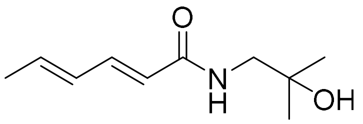

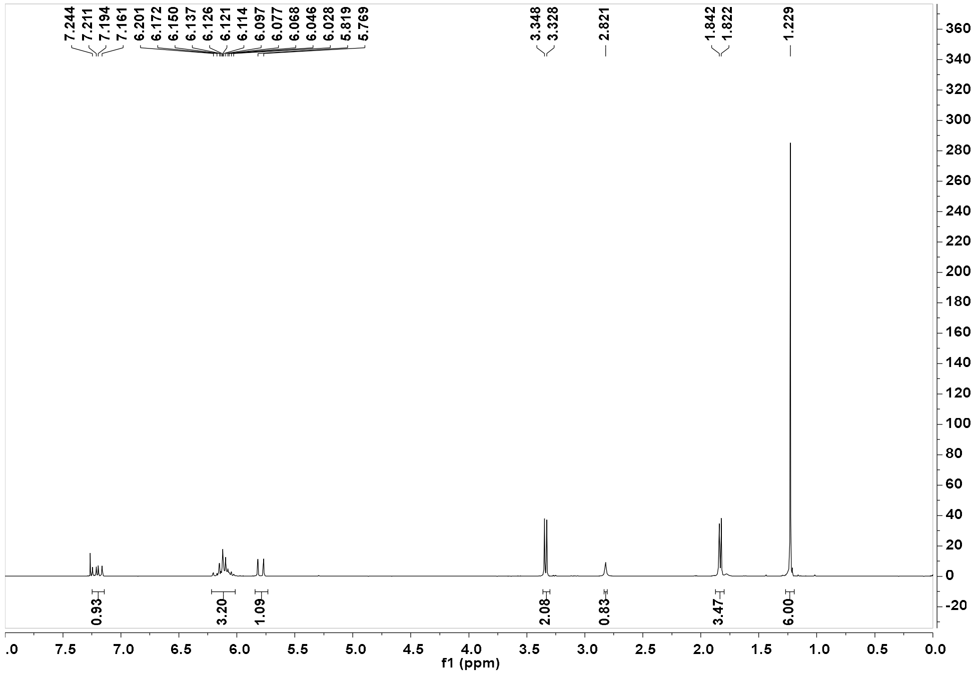

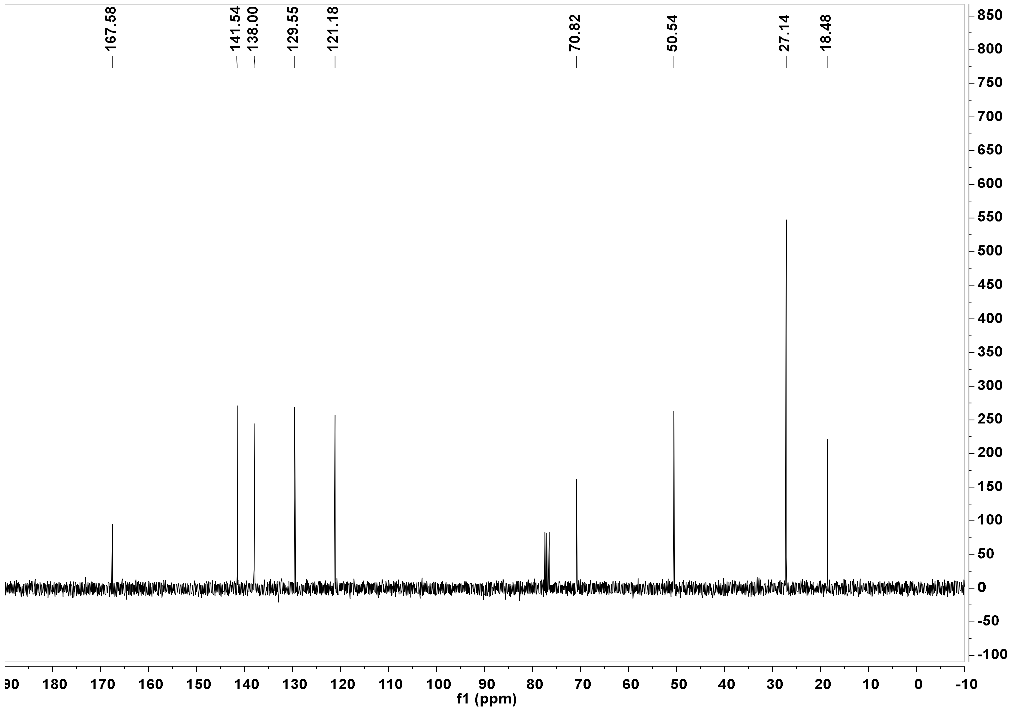

N-(2-hydroxy-2-methylpropyl)hexanamide (**3j**)

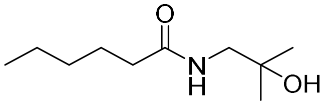
O
N
H
OH

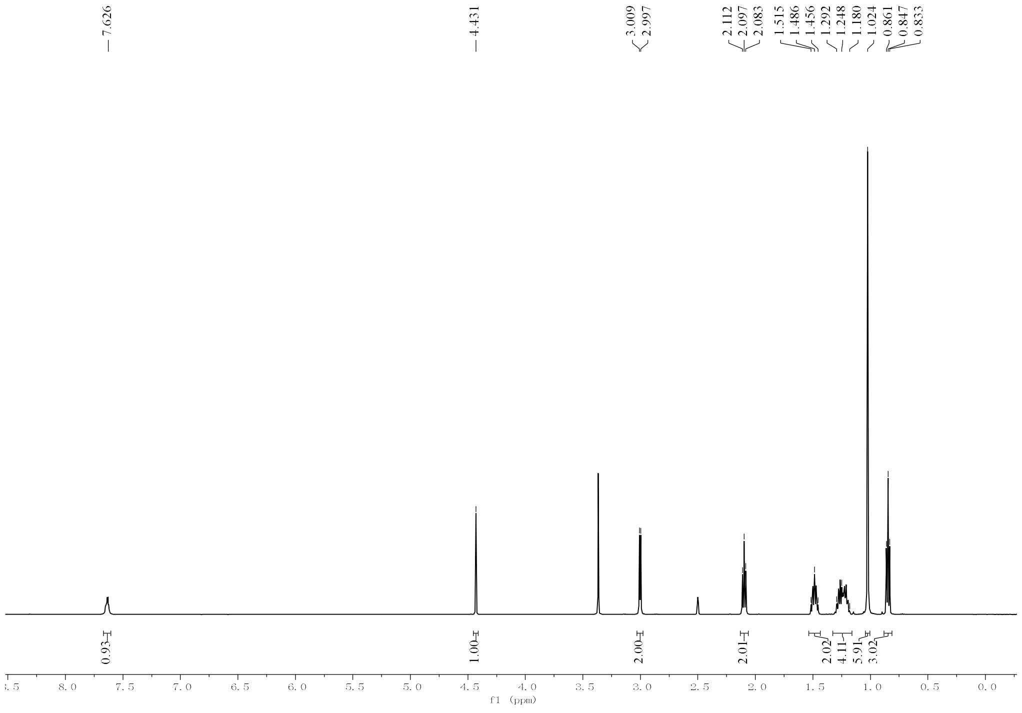
7.626
4.431
3.009
2.997
2.112
2.097
2.083
1.515
1.486
1.456
1.292
1.248
1.180
1.024
0.861
0.847
0.833
0.93
1.00
2.00
2.01
2.02
4.11
5.91
3.02
8.0
7.5
7.0
6.5
6.0
5.5
5.0
4.5
4.0
3.5
3.0
2.5
2.0
1.5
1.0
0.5
0.0
f1 (ppm)

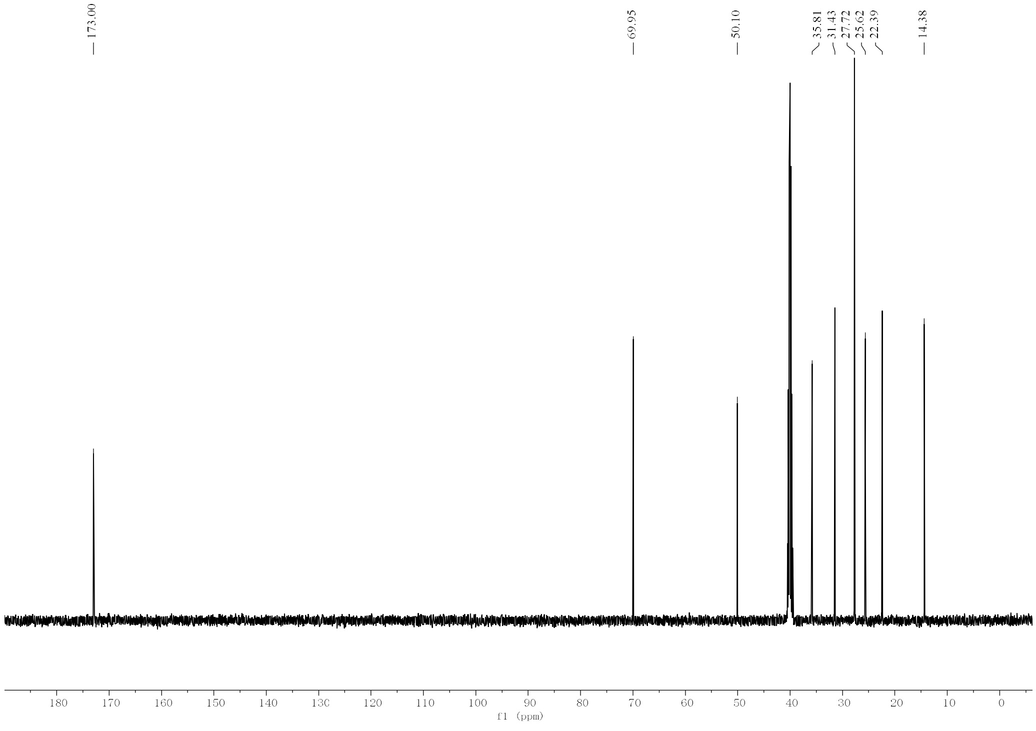
173.00
69.95
50.10
35.81
31.43
27.72
25.62
22.39
14.38
180
170
160
150
140
130
120
110
100
90
80
70
60
50
40
30
20
10
0
f1 (ppm)

N-(2-hydroxy-2-methylpropyl)heptanamide (**3k**)

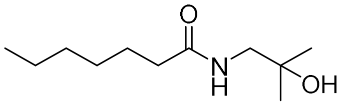

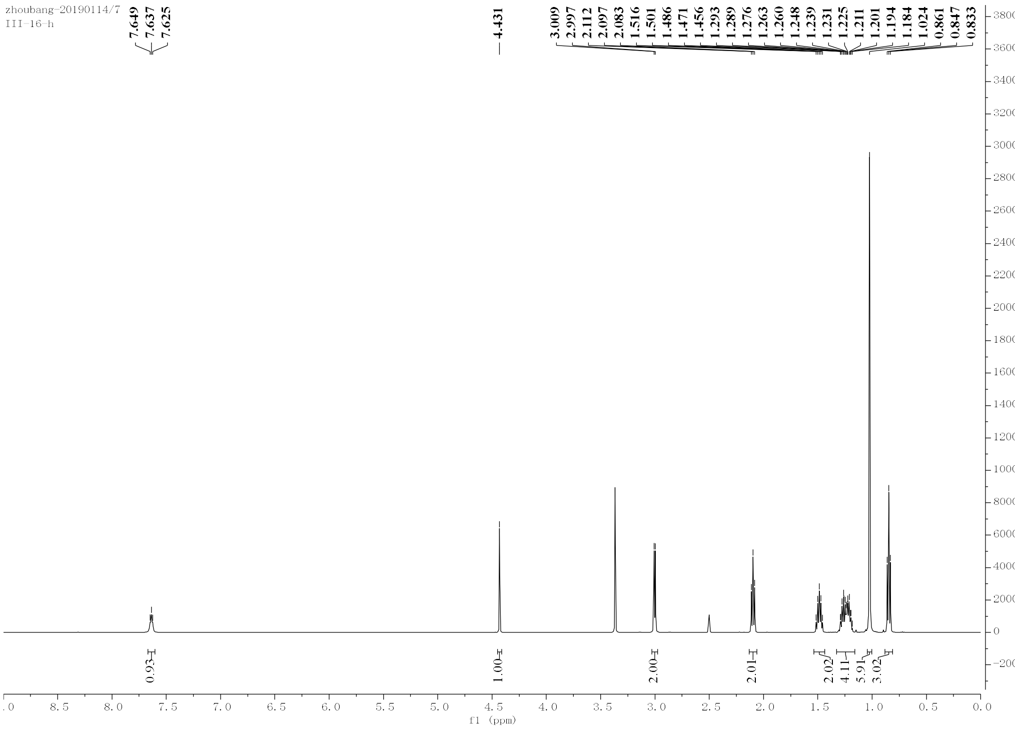

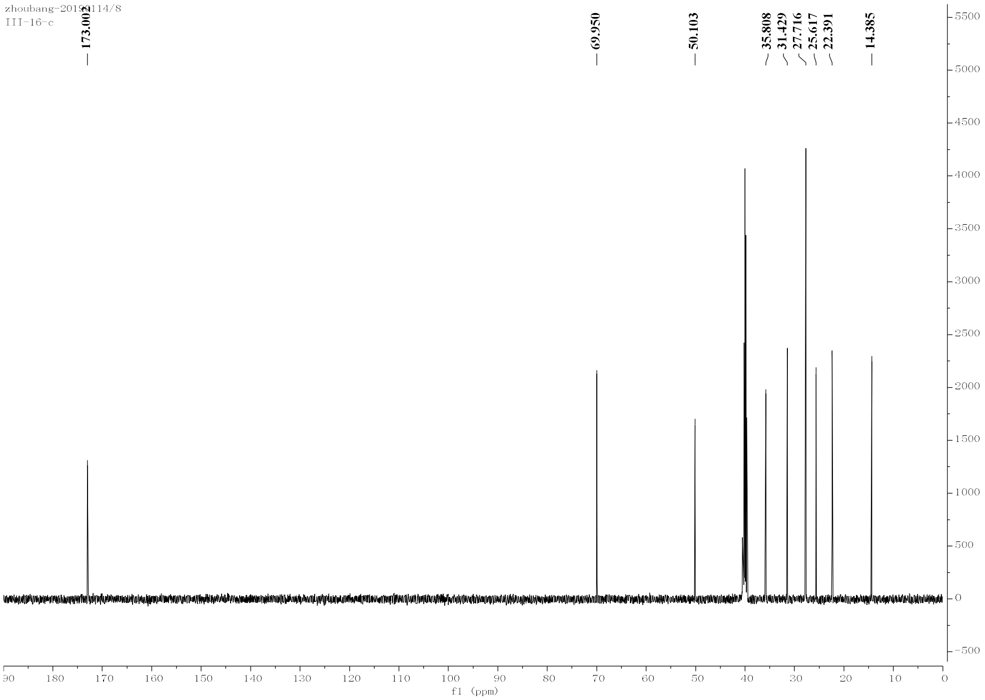

N-(2-methylallyl)acetamide (**4a**)

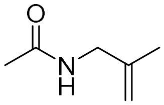

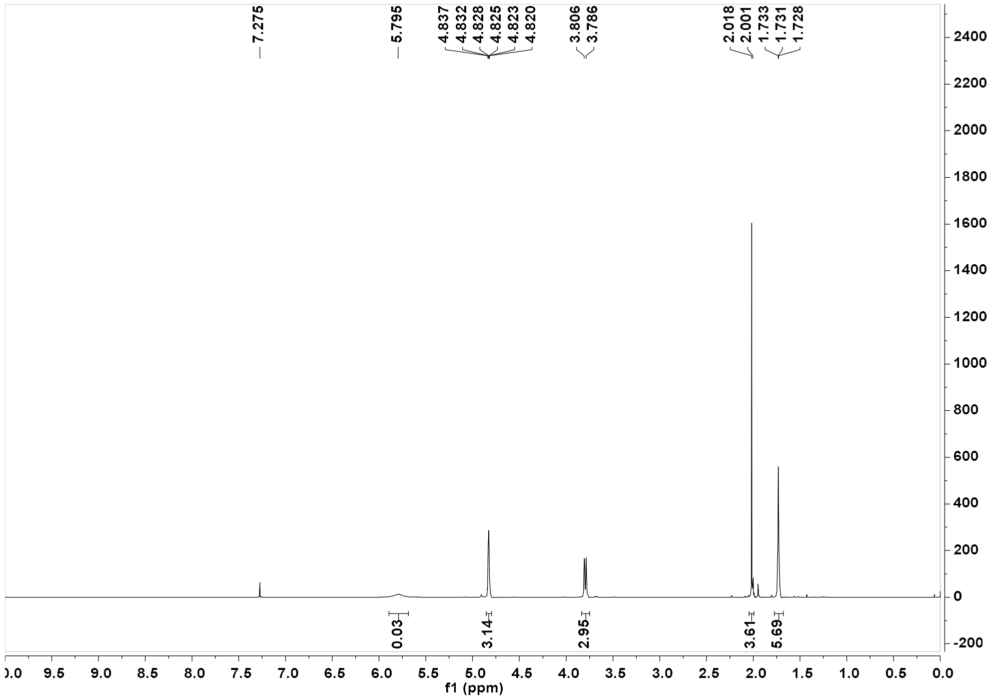

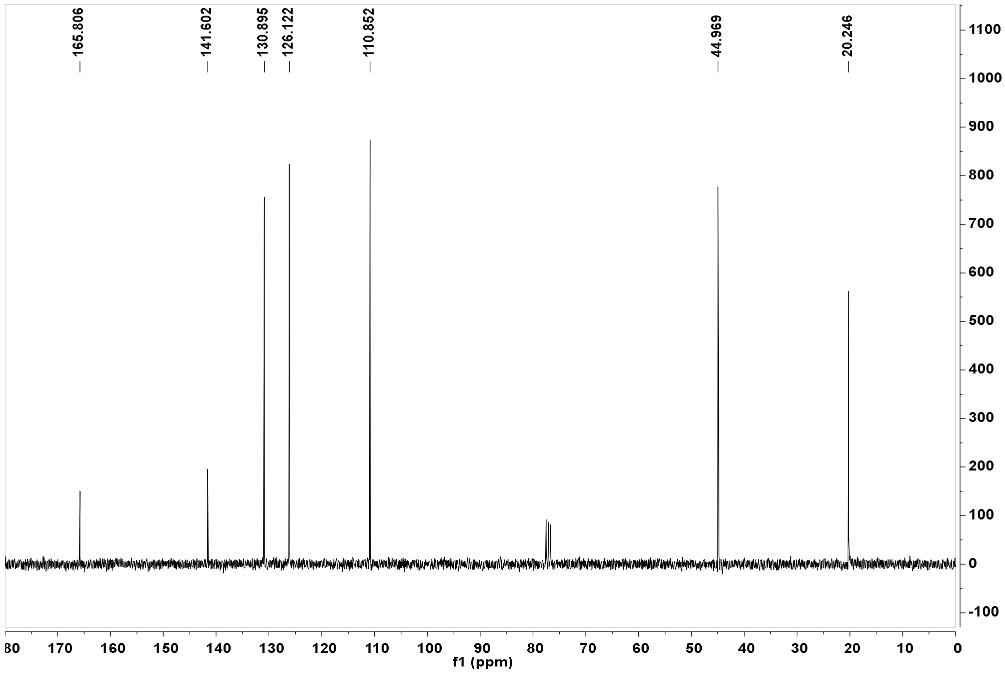

N-(2-methylallyl)acrylamide (**4b**)

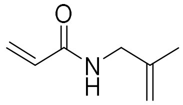
O
N
H

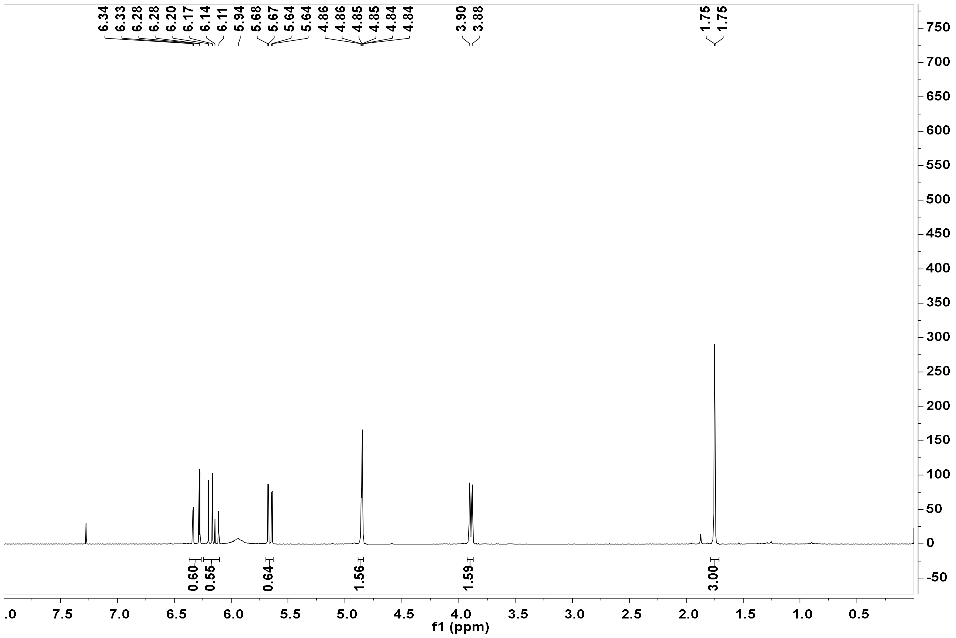
6.34
6.33
6.28
6.28
6.20
6.17
6.14
6.11
5.94
5.68
5.67
5.64
5.64
4.86
4.86
4.85
4.85
4.84
4.84
3.90
3.88
1.75
1.75
0.60
0.55
0.64
1.56
1.59
3.00
f1 (ppm)

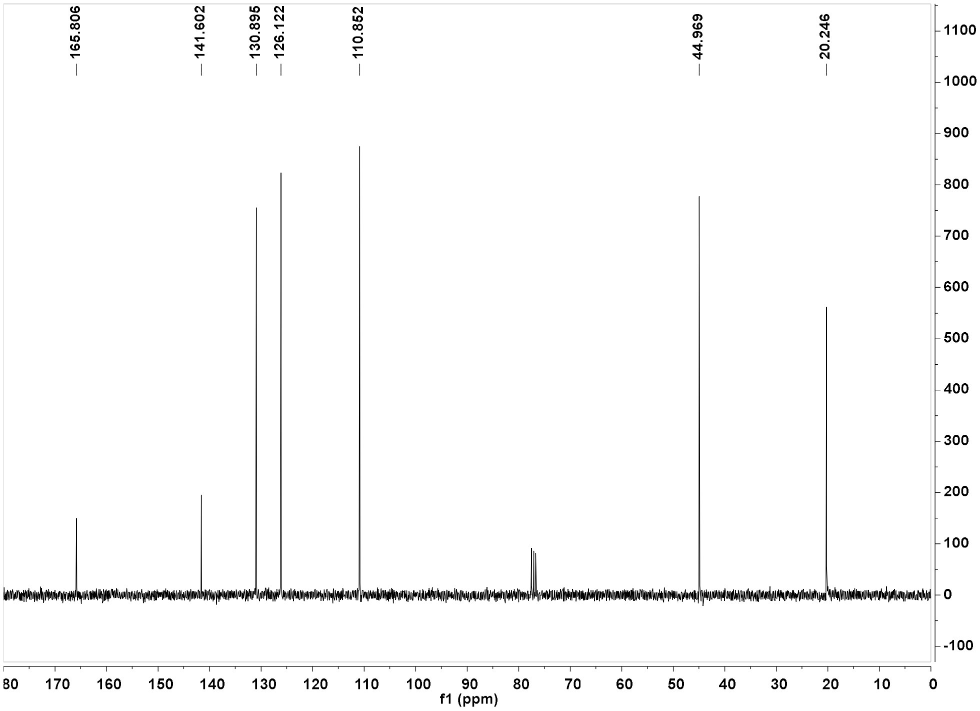
165.806
141.602
130.895
126.122
110.852
44.969
20.246
f1 (ppm)

N-(2-methylallyl)propionamide (**4c**)

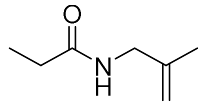

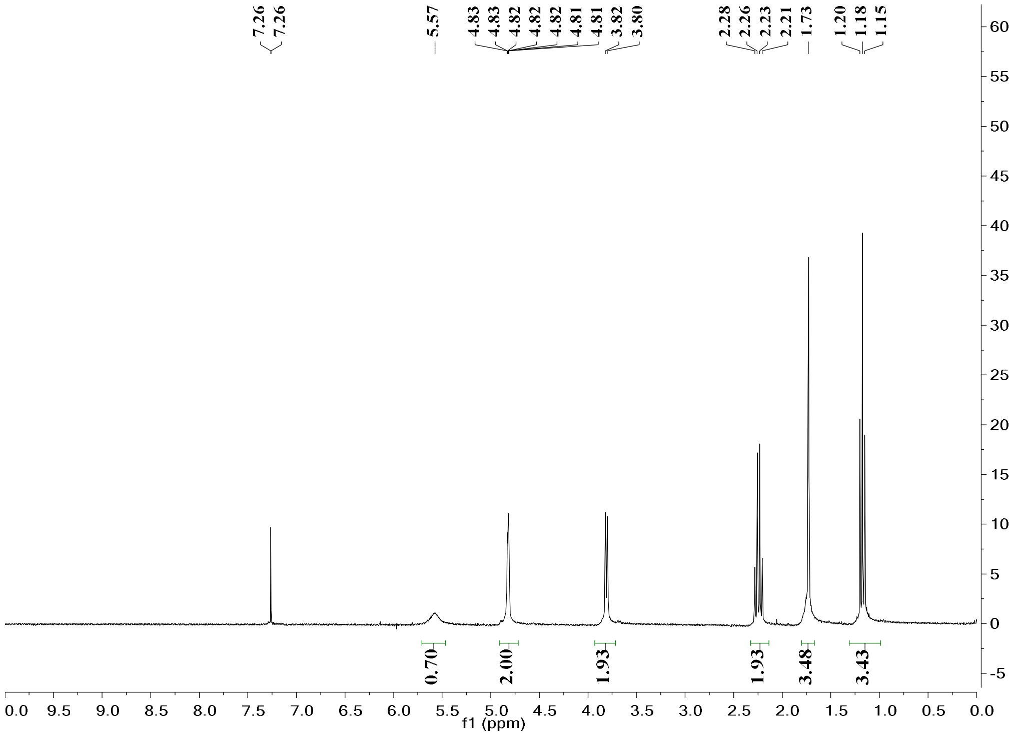

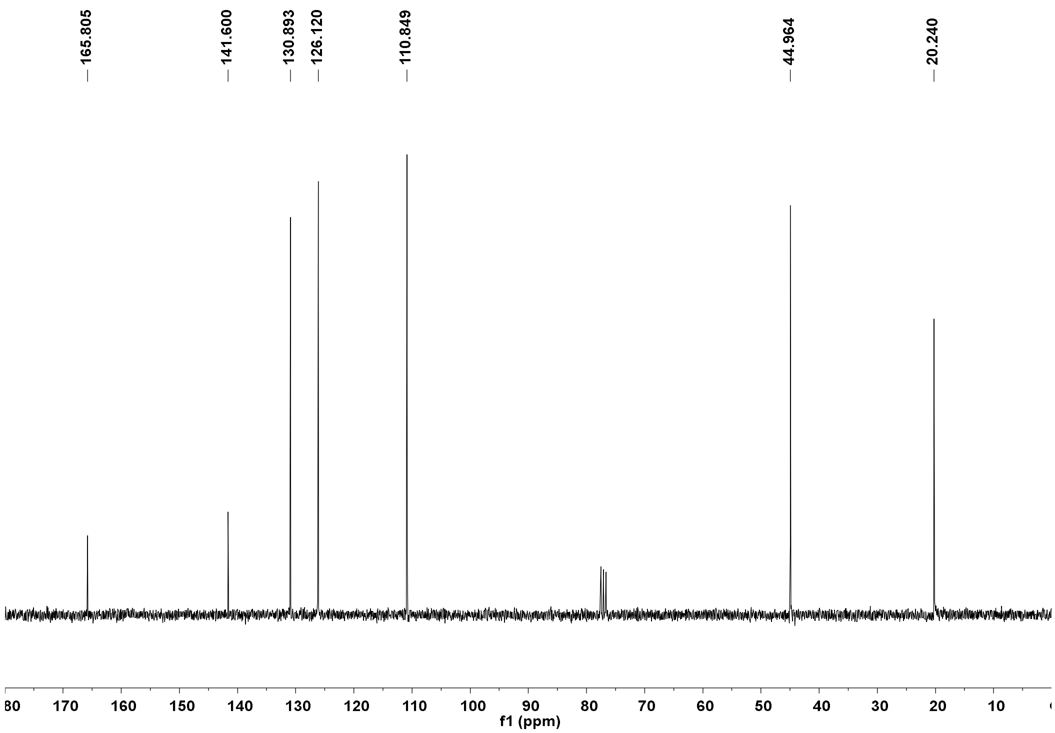

N-(2-methylallyl)butyramide (**4d**)

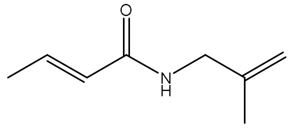

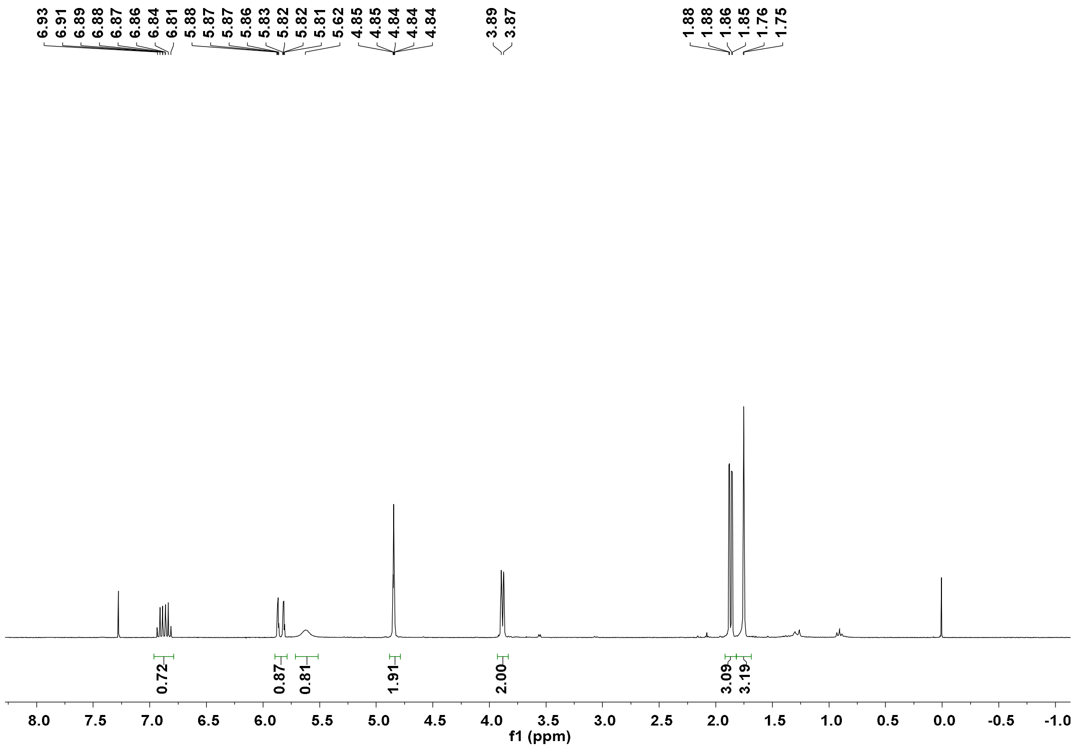

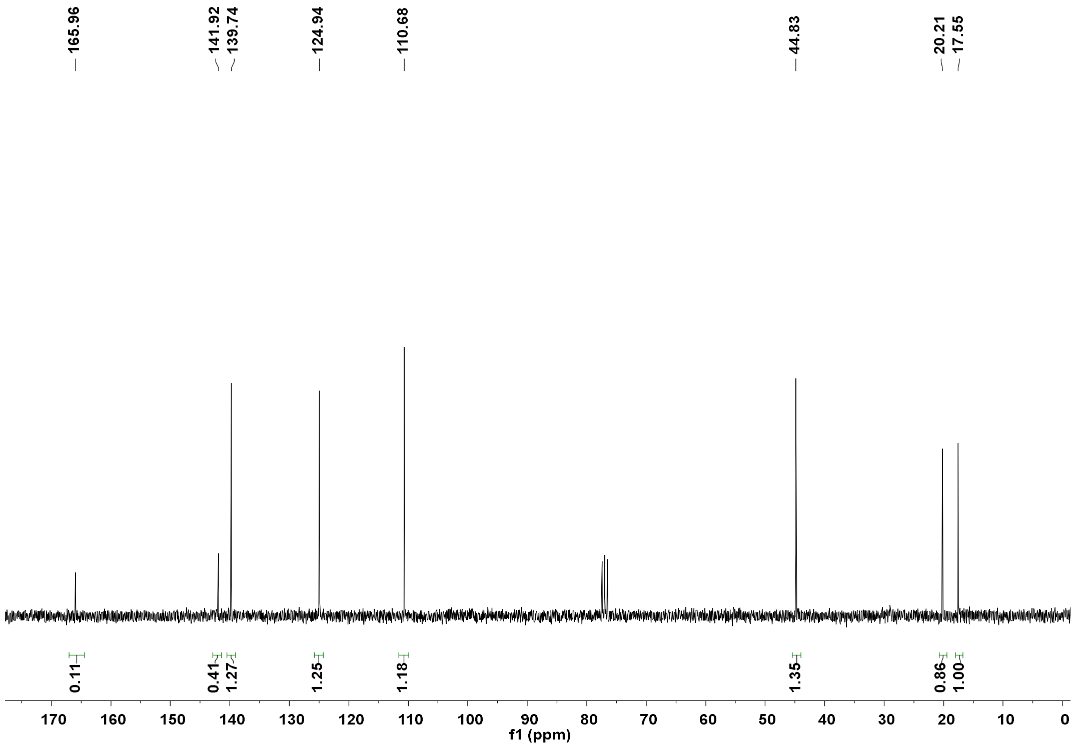

N-(2-methylallyl)butyramide (**4e**)

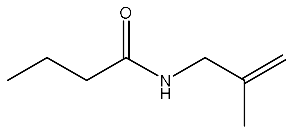

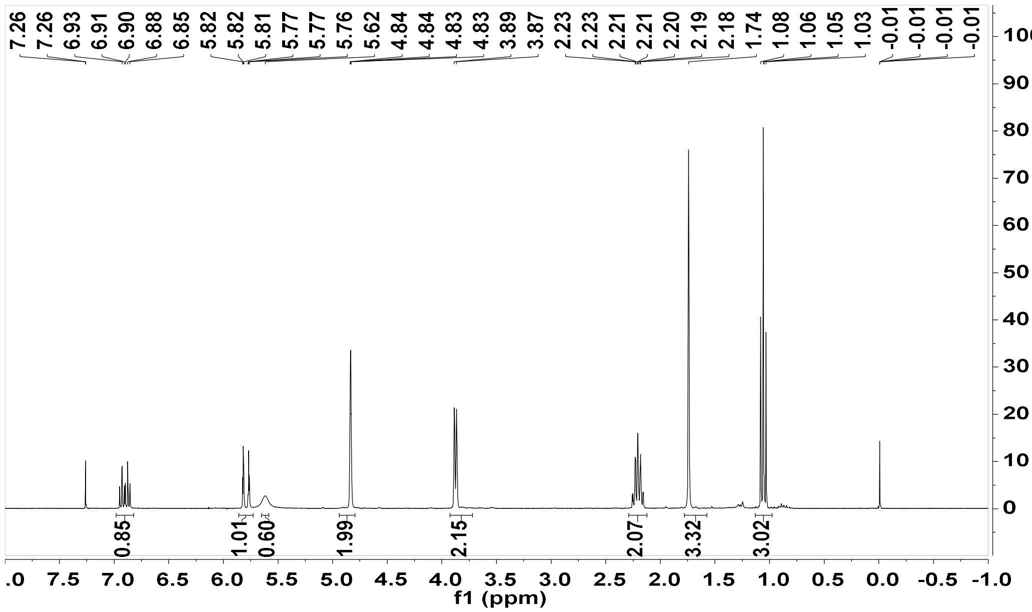

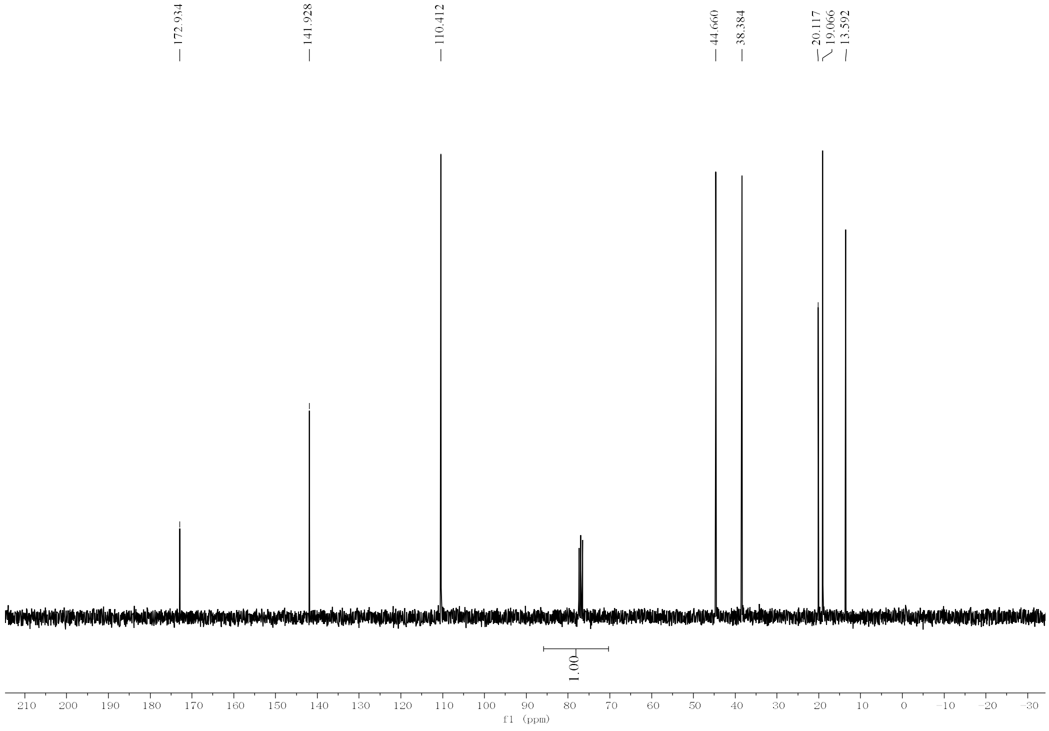

(*E*)-*N*-(2-methylallyl)pent-2-enamide (**4f**)

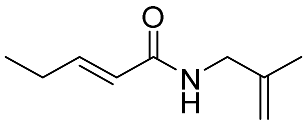
O
N
H

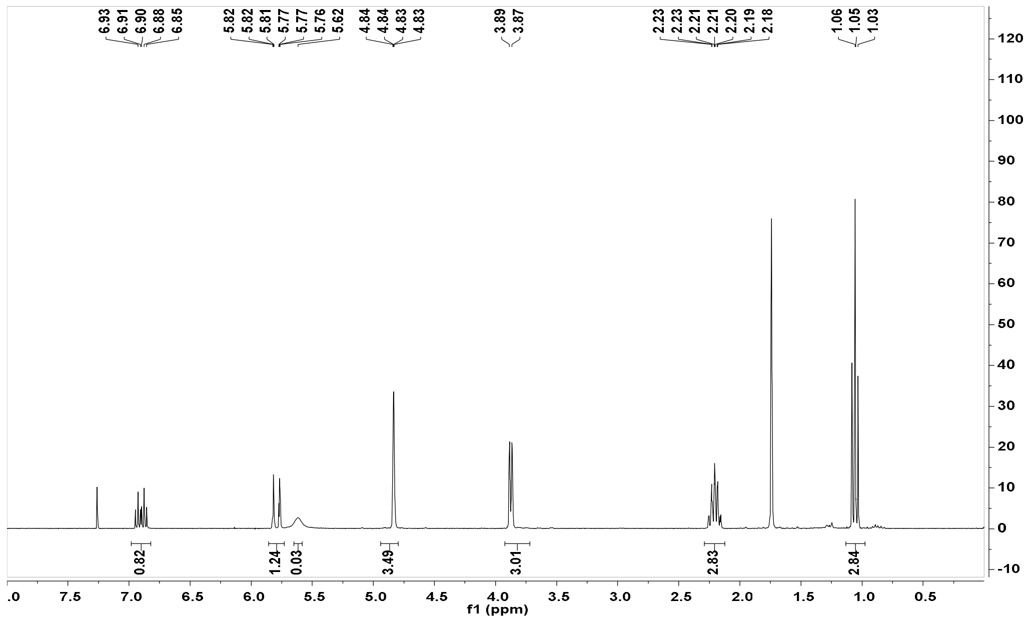
6.93
6.91
6.90
6.88
6.85
5.82
5.82
5.81
5.77
5.77
5.76
5.62
4.84
4.84
4.83
4.83
3.89
3.87
2.23
2.23
2.21
2.21
2.20
2.19
2.18
1.06
1.05
1.03
0.82
1.24
0.03
3.49
3.01
2.83
2.84
f1 (ppm)

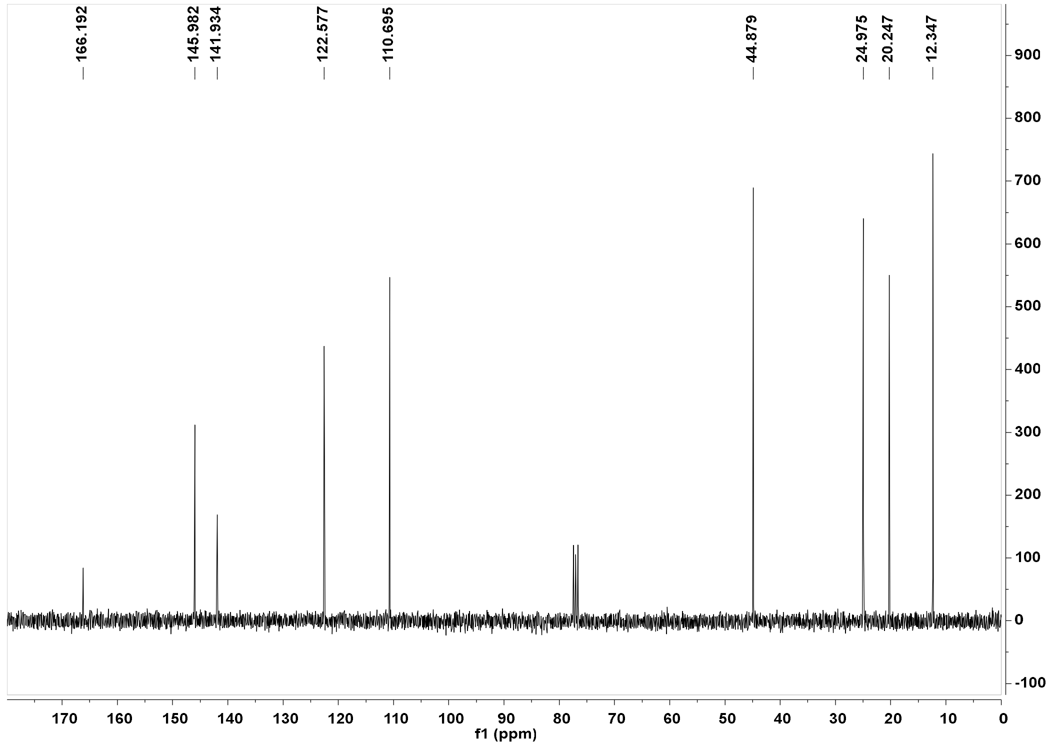
166.192
145.982
141.934
122.577
110.695
44.879
24.975
20.247
12.347
f1 (ppm)

N-(2-methylallyl)pentanamide (**4g**)

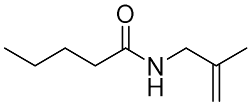

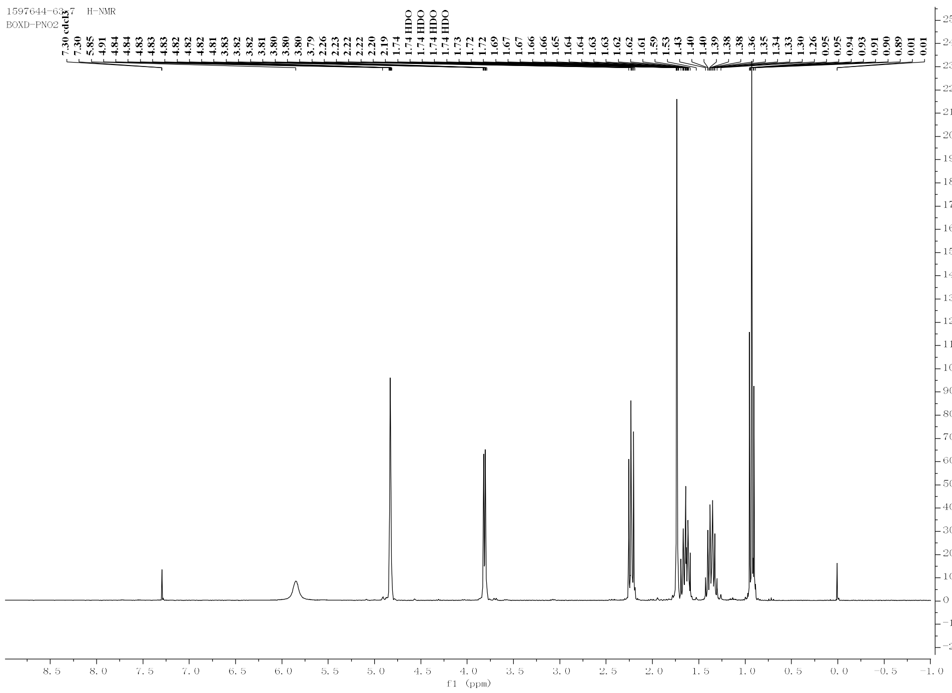

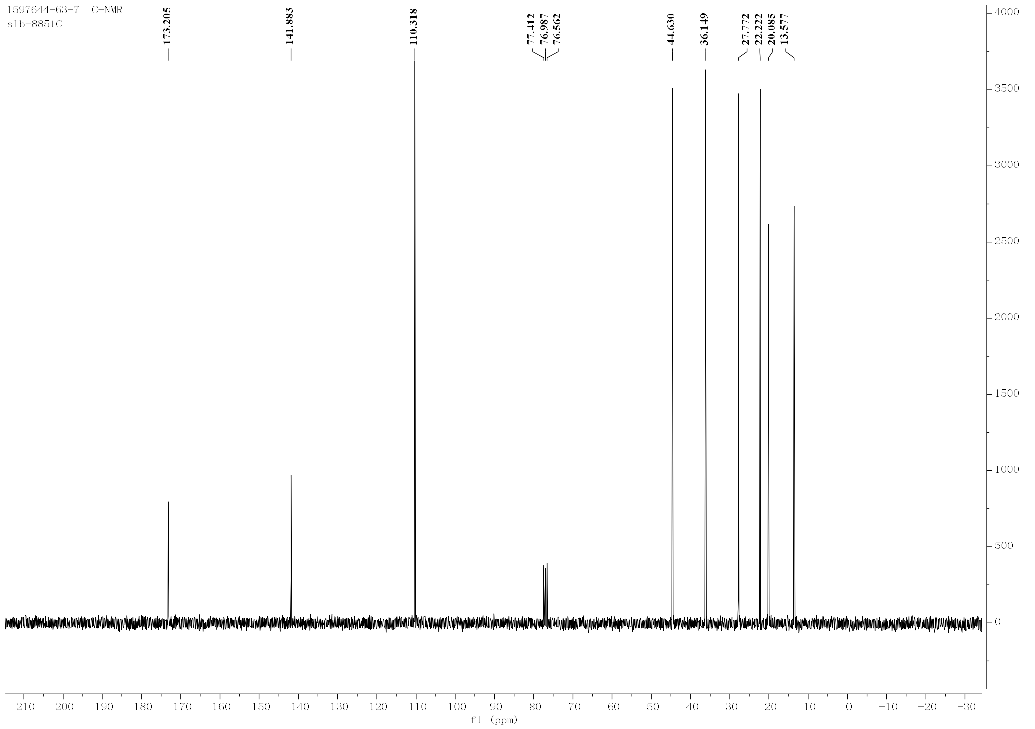

(*E*)-*N*-(2-methylallyl)hex-2-enamide (**4h**)

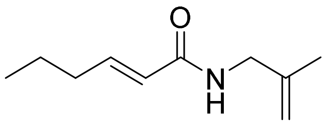

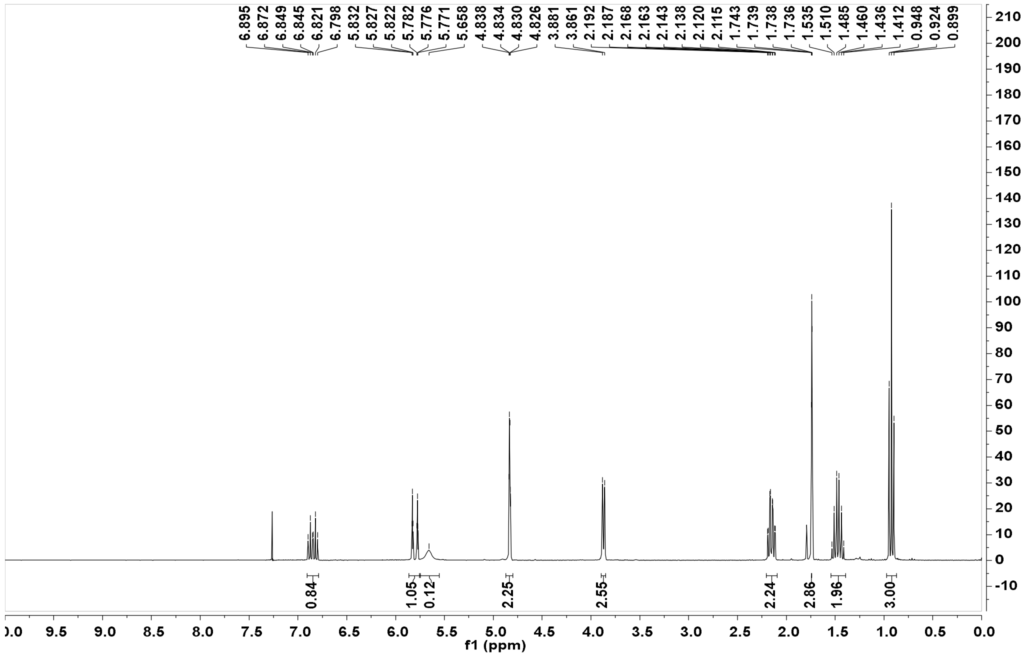

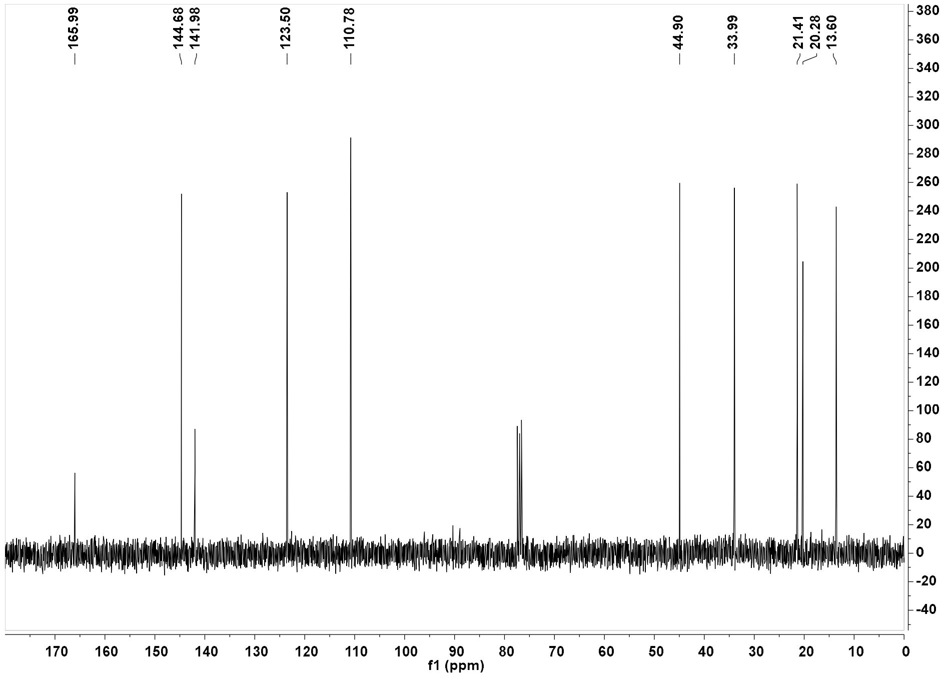

(2*E*,4*E*)-*N*-(2-methylallyl)hexa-2,4-dienamide (**4i**)

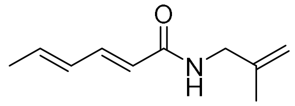

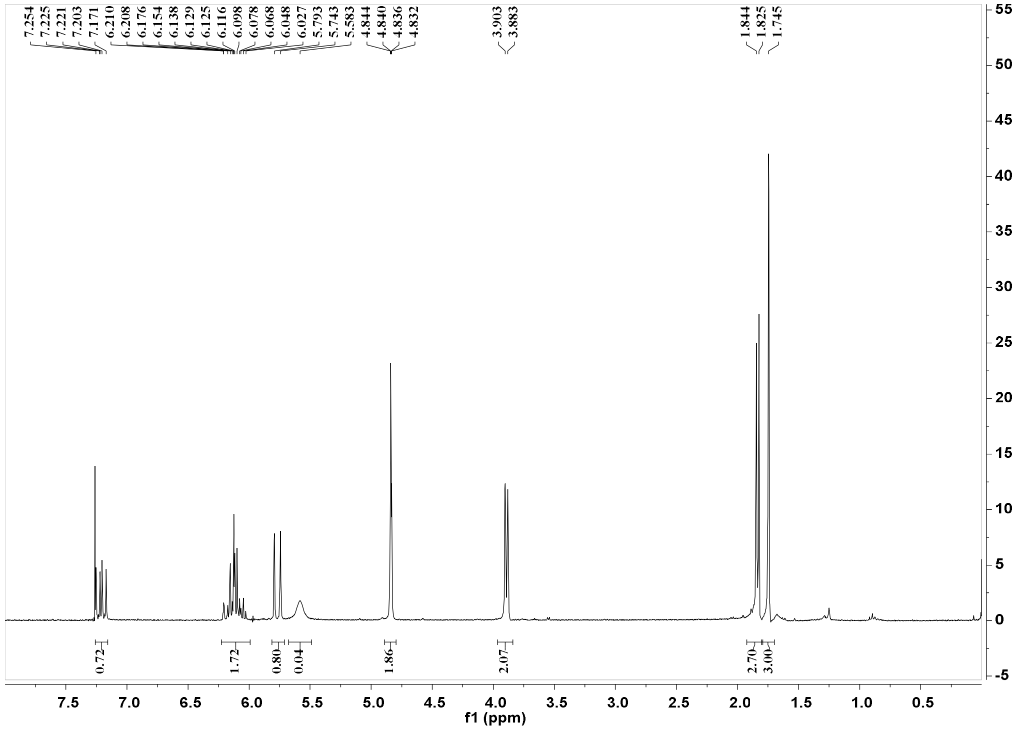

7.254
7.225
7.221
7.203
7.171
6.210
6.208
6.176
6.154
6.138
6.129
6.125
6.116
6.098
6.078
6.068
6.048
6.027
5.793
5.743
5.583
4.844
4.840
4.836
4.832
3.903
3.883
1.844
1.825
1.745
0.72
1.72
0.80
0.04
1.86
2.07
2.70
3.00
f1 (ppm)

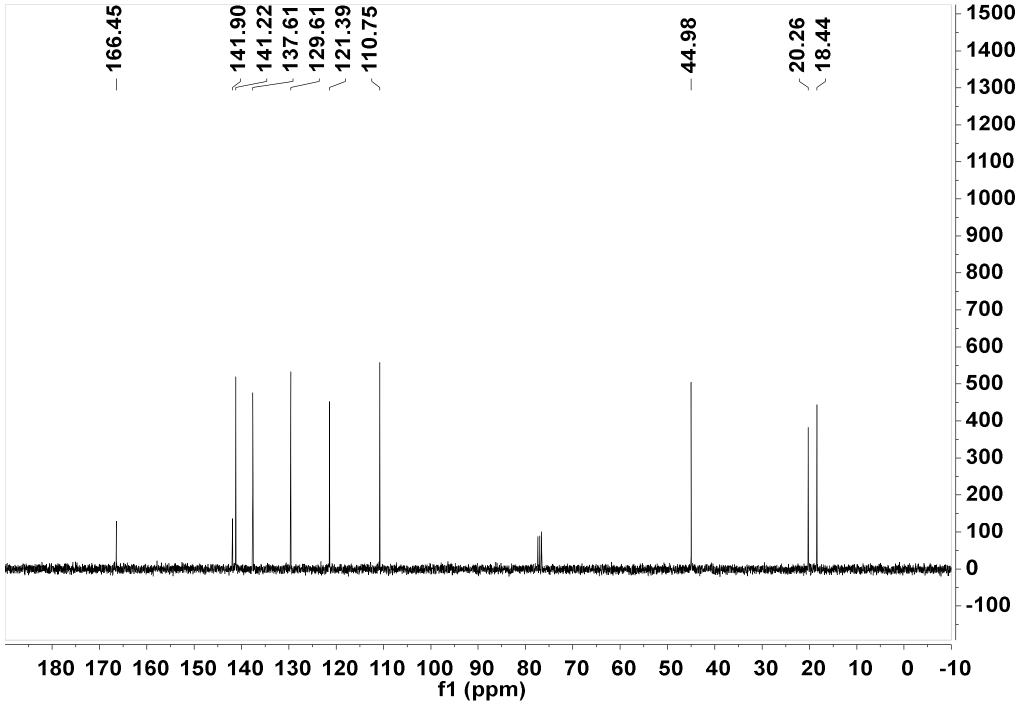

166.45
141.90
141.22
137.61
129.61
121.39
110.75
44.98
20.26
18.44
f1 (ppm)

N-(2-methylallyl)hexanamide (**4j**)

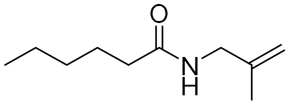

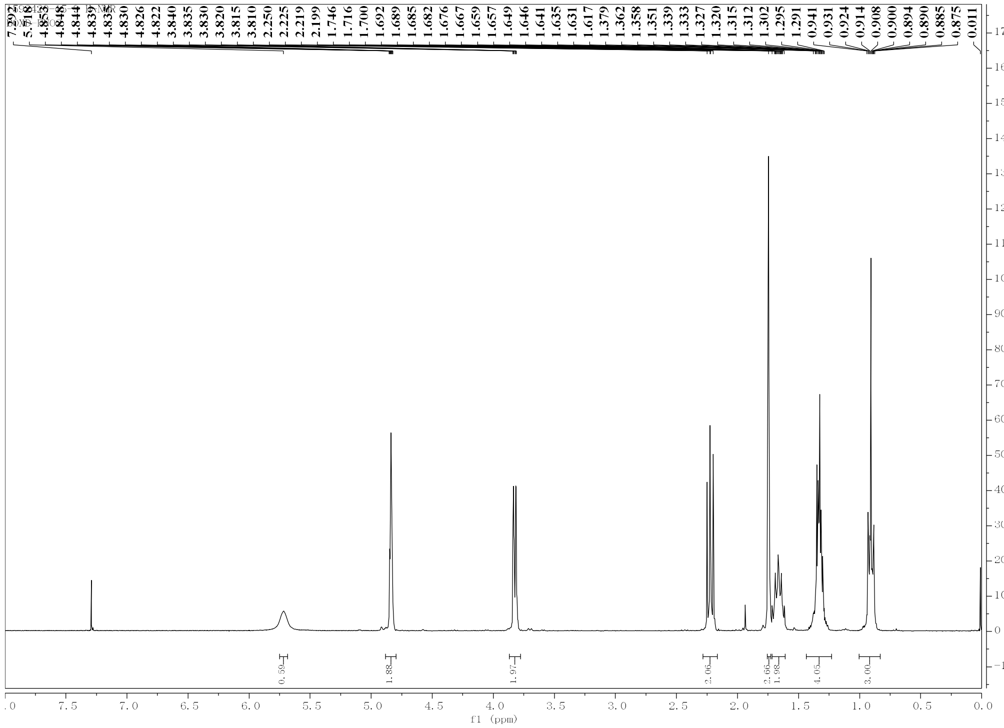

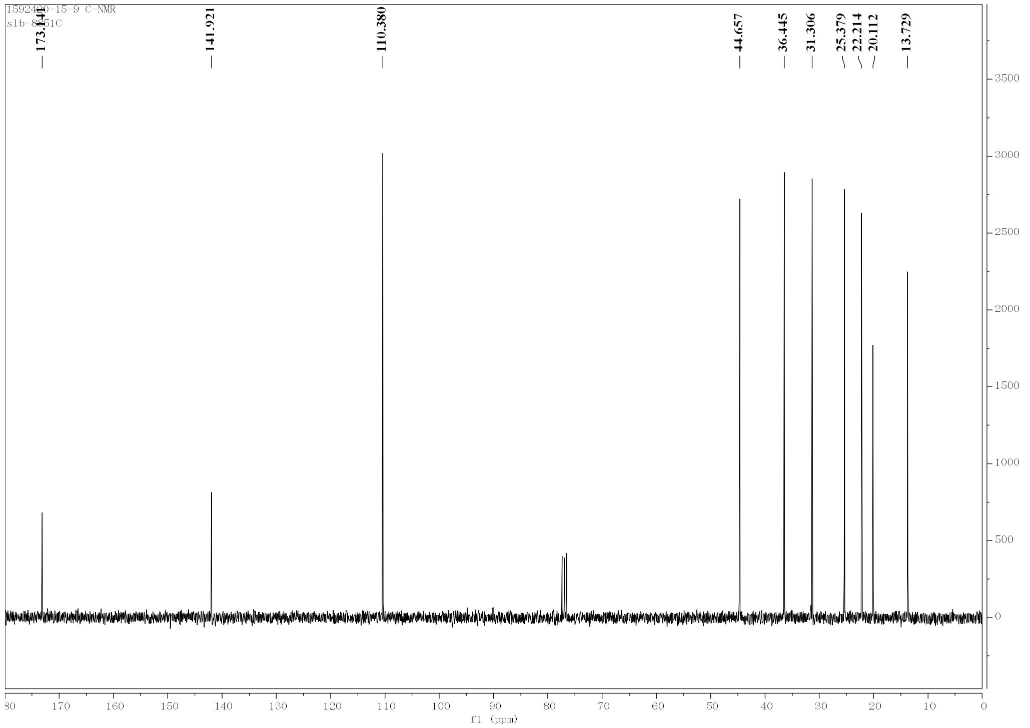

N-(2-methylallyl)heptanamide (**4k**)

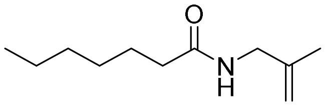

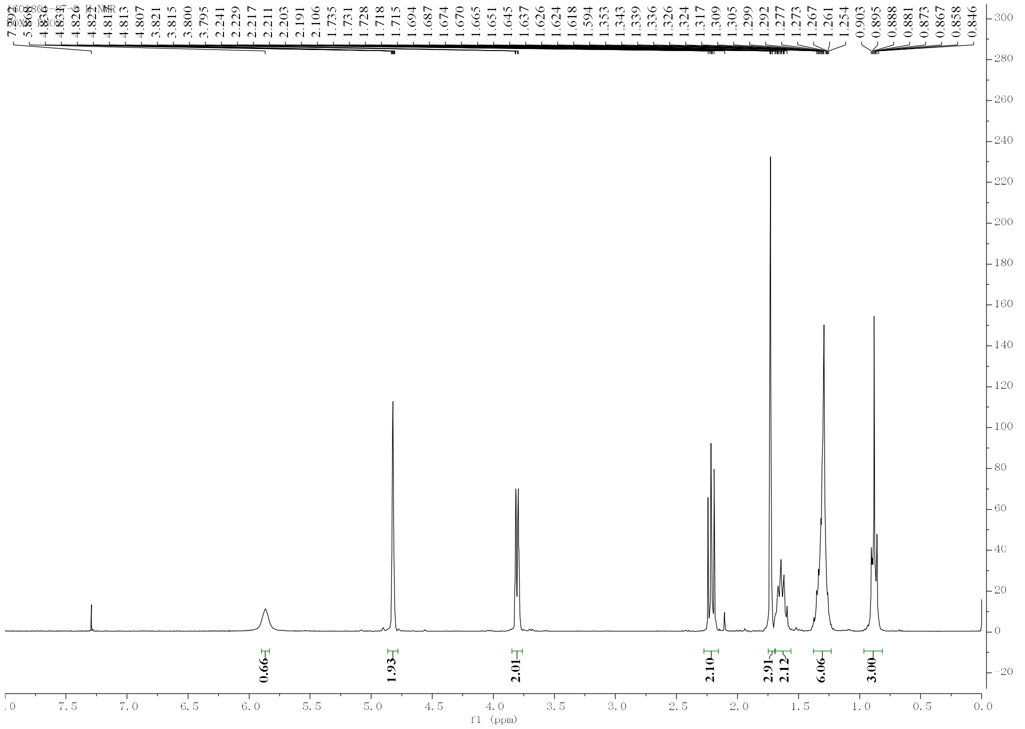

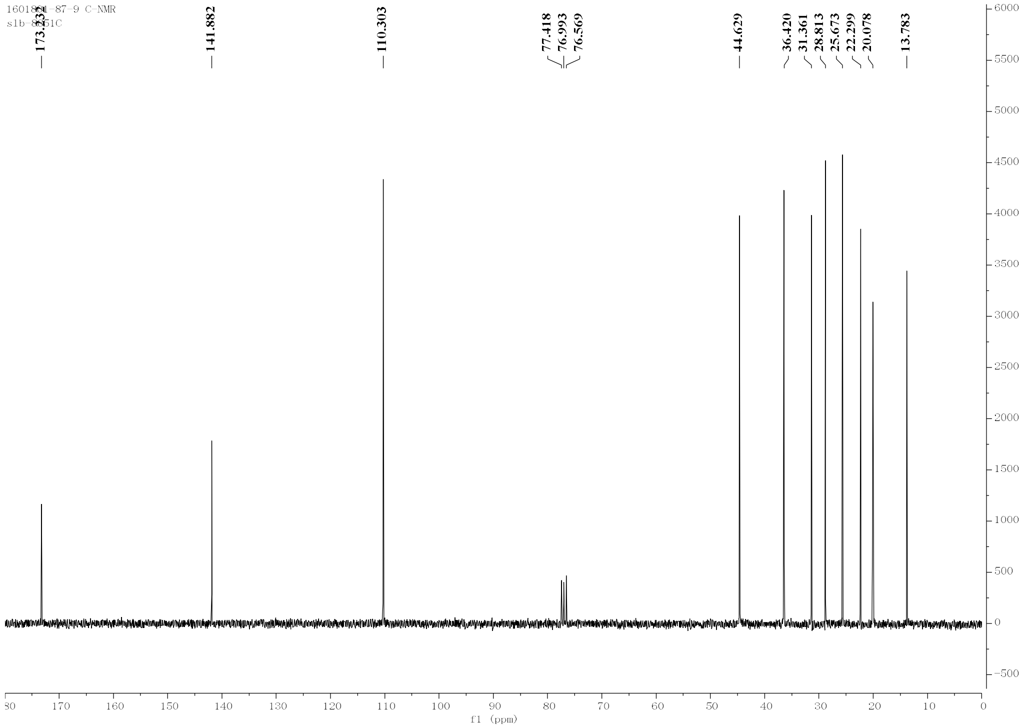

Supplement: Supplementary file 1 [file molecules-25-04986-s001.pdf]
